# Homologated amino acids with three vicinal fluorines positioned along the backbone: development of a stereoselective synthesis

**DOI:** 10.3762/bjoc.13.228

**Published:** 2017-11-01

**Authors:** Raju Cheerlavancha, Ahmed Ahmed, Yun Cheuk Leung, Aggie Lawer, Qing-Quan Liu, Marina Cagnes, Hee-Chan Jang, Xiang-Guo Hu, Luke Hunter

**Affiliations:** 1 School of Chemistry, The University of New South Wales, Sydney NSW 2052, Australia; 2 National Engineering Research Center for Carbohydrate Synthesis, Jiangxi Normal University, Nanchang, China; 3 School of Chemistry, The University of Sydney, Sydney NSW 2006, Australia

**Keywords:** amino acids, conformation, deoxyfluorination, fluorine, stereochemistry

## Abstract

Backbone-extended amino acids have a variety of potential applications in peptide and protein science, particularly if the geometry of the amino acid is controllable. Here we describe the synthesis of δ-amino acids that contain three vicinal C–F bonds positioned along the backbone. The ultimately successful synthetic approach emerged through the investigation of several methods based on both electrophilic and nucleophilic fluorination chemistry. We show that different diastereoisomers of this fluorinated δ-amino acid adopt distinct conformations in solution, suggesting that these molecules might have value as shape-controlled building blocks for future applications in peptide science.

## Introduction

**Figure 1:**
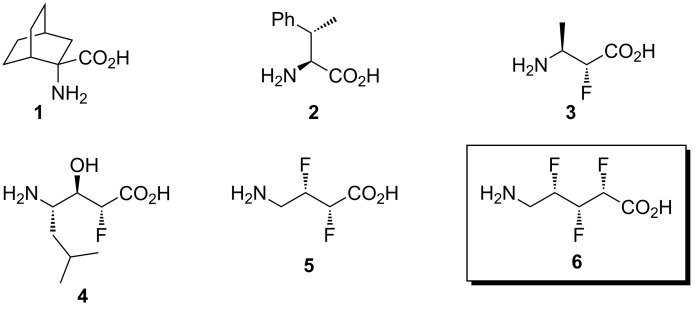
Examples of conformationally biased amino acids [1–10]. Compound **6** is a target of this work.

The incorporation of unnatural amino acids into a peptide structure can potentially reduce conformational disorder and hence improve the binding affinity of the peptide for its biological target. For example, conformationally rigid amino acids such as **1** (Figure 1) have been shown to dramatically affect the secondary structure of peptides within which they are contained, with consequent implications for the peptides’ biological potency and selectivity [1]. A more subtle example of this concept is provided by the amino acid β-methylphenylalanine (**2**), which exerts conformational bias through acyclic means; steric interactions associated with the β-methyl group can affect the topography of peptides which once again affects the biological affinity and selectivity [2].

Extending the idea of acyclic shape control, amino acids with homologated backbones (e.g., **3**–**5**, Figure 1) [3–10] provide opportunities for functionalisation in ways not possible in natural α-amino acids. There is the ability to place heteroatoms along the amino acid backbone, or to incorporate two or more functionalised side chains per amino acid residue, and this results in a variety of stereochemical configurations that can affect the conformation. Organofluorine chemistry offers a particular attraction here, since fluorinated molecules (e.g., **3**–**5**) tend to adopt predictable conformations due to hyperconjugative and/or dipole–dipole interactions associated with the C–F bond [11–15].

Such a progression in the study of fluorinated amino acids develops into the concept of α,β,γ-trifluoro-δ-amino acids (e.g., **6**, Figure 1). δ-Amino acids such as **6** are of special interest because they have the same backbone length as a dipeptide of α-amino acids, and thus may potentially be substituted for a two amino acid unit in a natural peptide without changing the overall length of the peptide [16]. The presence of three vicinal fluorine atoms on the amino acid backbone of **6** gives rise to eight possible stereoisomeric forms, which presents a synthetic challenge of stereocontrol. As an initial contribution towards the study of such compounds, we recently published a synthesis of two diastereoisomers of **6** (in protected form) [17]. We now disclose full details of the various synthetic approaches that were investigated towards the target **6**, and the extensive troubleshooting that was required even within the approach that was ultimately successful. We also present here, for the first time, a qualitative NMR *J*-based conformational analysis of the free amino acids including **6**.

## Results and Discussion

**Scheme 1:**
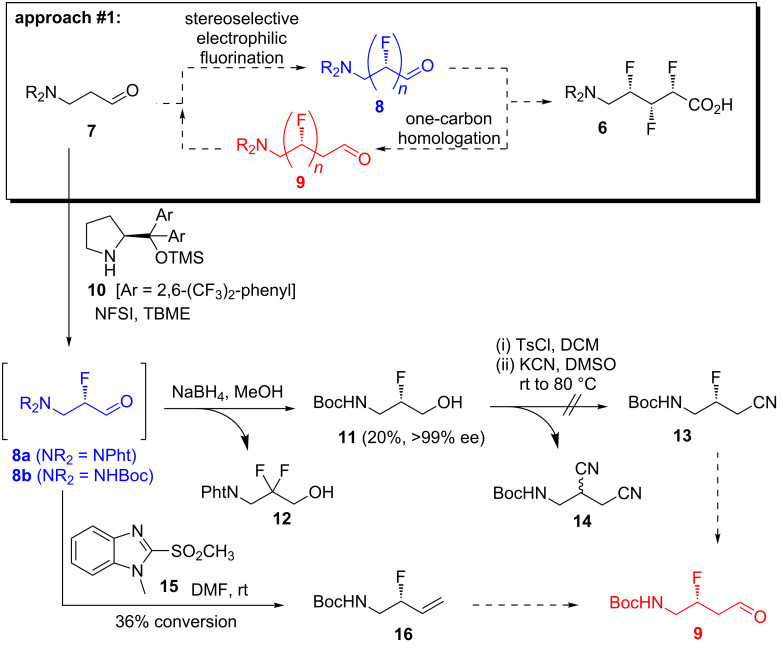
The first synthetic approach.

Early in our efforts to develop a successful synthesis of **6**, we realized that it might be possible to construct the repeating (CHF)*_n_* motif within the target molecule via an iterative synthetic approach (Scheme 1, boxed). We reasoned that an aldehyde such as **7** could undergo electrophilic fluorination, mediated by a chiral organocatalyst [18–20], to generate the fluorinated aldehyde **8** as a single stereoisomer. Then, if the carbon chain of **8** could be extended by one atom to give the homologated aldehyde **9**, fluorination could be repeated and the cycle could continue until the desired number of fluorine atoms was installed. This hypothetical approach had several attractions, including (i) the flexibility of being able to generate amino acids of different backbone lengths (e.g., **5**, **6**, Figure 1) via a unified strategy; (ii) an ability to access any stereoisomer of the target molecules (provided that the stereoselectivity in each fluorination step was catalyst-controlled); (iii) the lower toxicity of the electrophilic fluorination reagent NFSI (compared with nucleophilic fluorination reagents such as DeoxoFluor).

Accordingly, two aldehyde substrates (**7a** and **7b**) were prepared [21–22], containing either a phthalimide or a Boc protecting group. Electrophilic fluorination was attempted according to the method developed by Jørgensen and co-workers (Scheme 1) [20]. Thus, the aldehyde **7a** (or **7b**) was treated with *N*-fluorobenzenesulfonimide in the presence of the chiral organocatalyst **10**, and after a certain period the fluorinated aldehyde product **8** was reduced in situ. Initial studies with substrate **7a** (containing the phthalimide protecting group) suggested that the undesired difluorinated compound **12** was formed as the major product. An additional complication was that the phthalimide protecting group of **12** seemed to be at least partially sensitive to sodium borohydride [23]. In contrast, the substrate **7b** (containing the Boc protecting group) was successfully converted into the desired fluorohydrin **11**, albeit in poor yield. The optical purity of **11** was established through Mosher ester analysis (see Supporting Information File 1).

With the fluorohydrin **11** in hand (Scheme 1), the next task was to extend the carbon chain by one atom. The alcohol **11** was first converted into the corresponding tosylate (Scheme 1), but when this tosylate was subsequently treated with cyanide the undesired disubstituted product **14** was formed in 40% yield. Unfortunately, despite varying the reaction stoichiometry it was not possible to isolate any of the desired product **13**. It is possible that varying the reaction solvent might alter the reactivity profile, but this was not investigated in this study. We did explore a triflate leaving group in this reaction (not shown), but this gave a complex mixture of products upon treatment with cyanide. As a further disappointment, the disubstituted product **14** appeared to be racemic, which implied that an elimination–addition sequence had taken place, which in turn suggested that intermediates such as **9** might be rather unstable.

An alternative strategy for extending the carbon backbone was needed. Grubbs and co-workers recently showed that β-fluoroaldehydes (e.g., **9**, Scheme 1) can be synthesized in one step from allylic fluorides (e.g., **16**) via Wacker-type oxidation [24]. Other methods for converting allylic fluorides into β-fluoroaldehydes are also known [25–26]. Therefore we turned our attention to converting the fluorinated aldehyde **8b** (Scheme 1) into the allylic fluoride **16**. The crude fluorinated aldehyde **8b** was treated with a variety of olefination reagents (e.g., Tebbe; Wittig; reagent **15** [27]). Unfortunately, however, the desired allylic fluoride **16** was either not formed or was very unstable, which meant that the subsequent Wacker-type oxidation [24] to **9** could not be attempted.

Concurrent with the homologation attempts described above (Scheme 1), some model studies were performed (Table 1) to ascertain the feasibility of performing α-fluorinations on other β-fluorinated carbonyl compounds besides **9**. Thus, β-fluoroaldehyde **17** which was synthesized by an independent method (see Supporting Information File 1) was treated with NFSI and catalyst **10** according to Jørgensen’s fluorination protocol [20] (Table 1, entry 1). However, this resulted in a complex mixture of products within which the desired α,β-difluorinated product could not be identified. The alternative model substrate **18** (see Supporting Information File 1) was next investigated (Table 1, entry 2). Unfortunately, however, compound **18** proved unstable to silica and so it was not possible to obtain sufficiently pure material for a meaningful α-fluorination test reaction to be performed. The low stability of β-fluoroaldehydes appeared to be a general phenomenon, and so an attempt was next made to generate such a substrate in situ via the oxidation of β-fluoroalcohol **19** (Table 1, entry 3), followed immediately by a fluorination reaction. However, this did not yield any of the desired vicinal difluorinated material. It is possible that alternative electrophilic fluorinating reagents such as Selectfluor [28] could give different results, but this was not investigated in this work.

**Table 1:** Attempted α-fluorination of β-fluorocarbonyl compounds.

Entry	Substrate	Conditions	Outcome

1	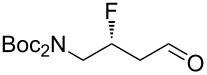 **17**	(i) **10**, NFSI, TBME, rt; (ii) NaBH_4_, MeOH	complex mixture
2	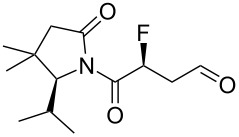 **18**	substrate **18** decomposed on silica, so no α-fluorination reactions could be attempted	N/A
3	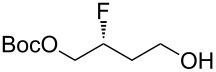 **19**	(i) PCC; (ii) **10**, NFSI, TBME, rt; (iii) NaBH_4_, MeOH	starting material **19** recovered
3	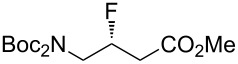 **20**	KO*t*-Bu, NFSI, THF, rt	starting material **20** recovered
4	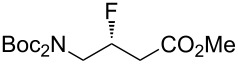 **20**	KHMDS, NFSI, THF, −78 °C	complex mixture

In a final attempt to develop an iterative fluorination/homologation strategy (Scheme 1, boxed), we considered whether an ester could be employed as the repeating unit, instead of an aldehyde. Accordingly, the model ester **20** (see Supporting Information File 1) was treated with an electrophilic fluorine source under basic conditions (Table 1, entries 3 and 4). Unfortunately, however, these attempts either returned unreacted starting material, or gave rise to a complex mixture of products, rather than the desired α,β-difluorinated ester.

Since major difficulties were encountered in both of the key steps of the proposed iterative fluorination/homologation approach (Scheme 1, boxed), we were forced to conclude that this was not a viable route to α,β,γ-trifluoro-δ-amino acids **6**.

**Scheme 2:**
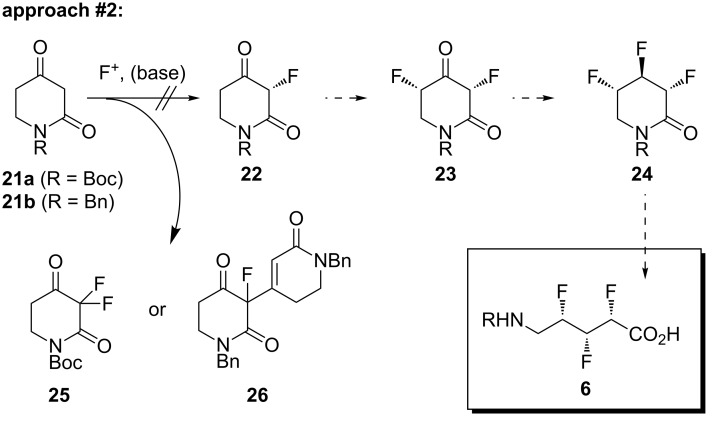
The second synthetic approach.

The next approach that was investigated is shown in Scheme 2. Having learned that homologation reactions involving fluorinated substrates were not facile, we decided to start the new approach with a full-length carbon chain in the form of piperidinedione **21**. We envisaged that a sequence of reactions – two electrophilic fluorinations [29–31] followed by reduction and deoxyfluorination – would deliver the target molecule **6**.

Accordingly, two piperidinedione substrates (**21a** and **21b**) were prepared [32–33], containing a Boc or a benzyl protecting group, respectively (Scheme 2). Substrate **21a** was first treated with Selectfluor in acetonitrile according to a mild protocol developed by Smith and co-workers for the α-fluorination of ketones [31]. However, ^1^H NMR and ^19^F NMR analysis of the crude reaction mixture revealed that the only identifiable product was the undesired *gem*-difluorinated compound **25** (Scheme 2), which was obtained along with a significant amount of unreacted starting material **21a** (see Supporting Information File 1). When the alternative substrate **21b** was exposed to a variety of different electrophilic fluorinating conditions (Scheme 2), a new reaction outcome was observed: in this case, the only identifiable product was the undesired dimeric species **26**, which was consistently obtained in reasonably high yields (see Supporting Information File 1). This product presumably arose through aldol condensation of the readily enolisable ketone **22** with another molecule of **21**. Overall then, it was concluded that approach #2 was not a viable strategy for synthesising target **6**. Alternative substrates based on the piperidine-2,5-dione scaffold might prove more tractable in the future, but this has not yet been investigated in our laboratories.

**Scheme 3:**
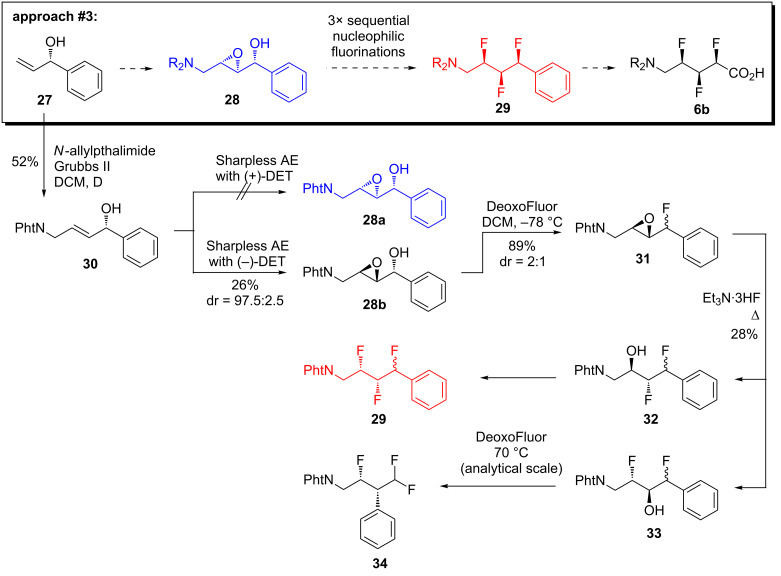
The third synthetic approach.

Since the first two approaches to target **6** (Scheme 1 and Scheme 2) were unsuccessful, we reasoned that a better-precedented synthetic method was needed. O’Hagan and co-workers have previously reported a concise method for synthesising compounds that contain three vicinal C–F bonds [34]; their method commences with an epoxy alcohol, which undergoes three successive nucleophilic substitutions with fluoride (i.e., deoxyfluorination of the alcohol, epoxide ring opening with fluoride, then deoxyfluorination). We therefore sought to apply O’Hagan’s method to the target **6b** (Scheme 3, boxed).

Accordingly, the enantiopure allylic alcohol **27** [35] was extended through a cross-metathesis reaction to deliver the disubstituted alkene **30** (Scheme 3). Compound **30** became the substrate for an attempted Sharpless asymmetric epoxidation reaction using (+)-DET (Scheme 3); however, none of the desired product **28a** was observed in this case, presumably due to a substrate/catalyst mismatch effect. Therefore, the epoxidation reaction was re-attempted using (−)-DET (Scheme 3); this successfully afforded the *syn*,*anti*-epoxy alcohol **28b** with good stereoselectivity, albeit in poor yield. One reason for the low yield of **28b** was the difficulty in its chromatographic separation from the byproducts of the epoxidation reaction. Nevertheless, a sufficient quantity of **28b** was obtained to proceed some way with the synthesis. Compound **28b** was treated with DeoxoFluor at low temperature, in order to affect a deoxyfluorination of the benzylic alcohol. This reaction gave the product **31** in high yield, but unfortunately with poor stereoselectivity, presumably due to a competing S_N_1-type reaction mechanism [36–37]. This reaction was not fully optimised; instead, the available quantity of the fluoroepoxide **31** was carried forward so that some idea could be obtained about the feasibility of the subsequent steps in the synthesis. Thus, the fluoroepoxide **31** (as a mixture of diastereoisomers) was treated with Et_3_N·3HF according to O’Hagan’s method [34] (Scheme 3). This did effect epoxide-opening to some extent, but the reaction was rather unsatisfactory because it was low-yielding and non-regioselective, which made full characterisation of the product mixture (**32**/**33**) impossible. Nevertheless, an analytical-scale final fluorination reaction was attempted (Scheme 3) because this was anticipated to converge some of the compounds into a simpler product mixture. Analysis of the crude reaction mixture by ^19^F NMR revealed that the desired product **29** may have been formed in small quantity. However, there was clear evidence that a *gem*-difluorinated compound had also formed: presumably this was compound **34** arising through neighbouring group participation and migration of the phenyl group [38]. A similar problem was encountered in the synthesis of α,β-difluorinated-γ-amino acids (e.g., **5**, Figure 1), which was being investigated in parallel [5–6].

**Scheme 4:**
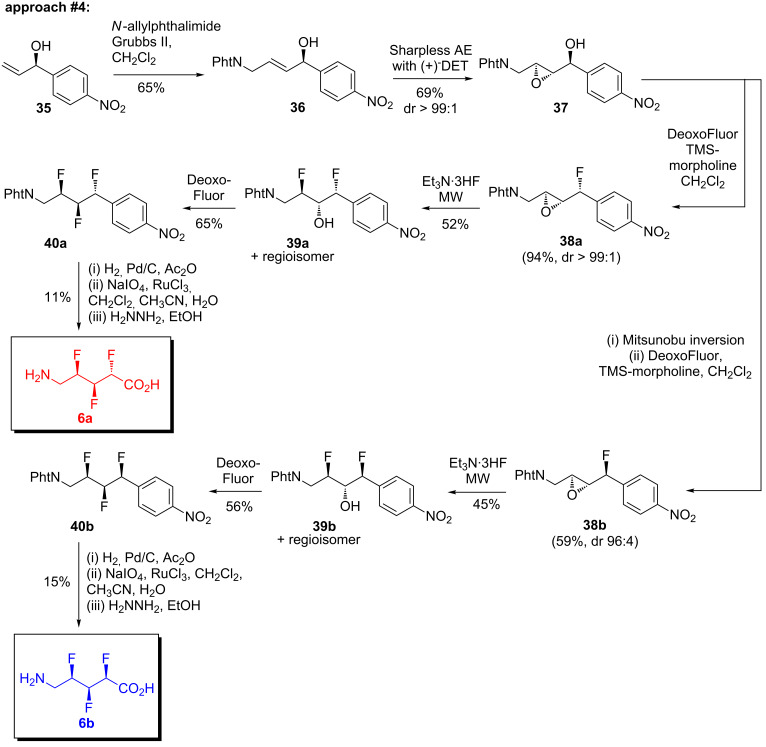
The fourth synthetic approach (partially reproduced from ref. [17]).

At this stage, it was clear that O’Hagan’s method [34] (Scheme 3) was the most promising strategy that had been examined so far. But four major obstacles remained: first, the starting material **27** was volatile and difficult to stockpile; second, the purification of epoxy alcohol **28b** was troublesome; third, the fluorination of **28b** proceeded with poor stereoselectivity; and fourth, the final fluorination reaction suffered from an undesired rearrangement side-reaction. We subsequently found that all four of these problems could be solved by making a single change to the synthesis: namely, by introducing a *p*-nitro group onto the aryl ring of the starting material, **35** (Scheme 4) [17].

A benefit of the *p*-nitro group immediately became apparent: the starting material **35** [35] (Scheme 4) was less volatile and hence easier to stockpile than its unsubstituted counterpart **27** (Scheme 3). Compound **35** was carried through the same set of reactions that were described previously for substrate **27** (Scheme 3). Thus, **35** underwent a cross metathesis reaction to furnish **36** in good yield (Scheme 4). Compound **36** then became the substrate for a Sharpless asymmetric epoxidation reaction, which delivered **37** with very high stereoselectivity (Scheme 4). The *p*-nitro group of **37** played another useful role here: compound **37** was rather insoluble, so it could be efficiently purified simply by triturating the crude product mixture with toluene, a procedure which afforded **37** in much higher yield than was obtained for the epoxy alcohol **28b** lacking the *p*-nitro group (Scheme 3). Compound **37** then underwent the first deoxyfluorination reaction to give compound **38a** in excellent yield (Scheme 4). The presence of the *p*-nitro group did improve the stereoselectivity of this reaction somewhat, but it was found that the inclusion of the additive TMS-morpholine [36–37] was also required to ensure a high diastereoisomeric excess of **38a**. The epoxide **38a** was then ring-opened using Et_3_N·3HF to deliver the difluorodiol **39a** as a mixture of regioisomers. This mixture subsequently converged during the next deoxyfluorination reaction (Scheme 4). Gratifyingly, the *p*-nitro group of **39a** was found to completely shut down the neighbouring group participation pathway; the desired trifluoroalkane **40a** was obtained in good yield with no evidence of rearrangement or epimerization.

It was also possible to modify the synthesis shown in Scheme 4 to produce the all-*syn* trifluoroalkane **40b**. Thus, the alcohol **37** underwent a Mitsunobu-type inversion of configuration, and O’Hagan’s series of three consecutive fluorination reactions [34] were subsequently applied to successfully deliver the all-*syn* trifluoroalkane **40b** (Scheme 4) [17].

Trifluoroalkanes **40a** and **40b** (Scheme 4) were advanced intermediates along the route towards the target trifluorinated amino acids (**6**). To complete the synthesis, the final requirements were to oxidise the aryl moiety into a carboxylic acid, and to deprotect the amino group. However, the *p*-nitro group of **40a**,**b** now posed a complication, because aryl oxidation reactions are only facile for electron-rich systems [39–40]. Unsurprisingly, when the oxidation reaction was attempted under standard NaIO_4_/RuCl_3_ conditions [39–40] with the nitroaryl substrate **40a**, no reaction was observed and the starting material was recovered intact.

Therefore, in order to identify a suitable method converting **40a**,**b** into **6a**,**b** (Scheme 4), model studies was undertaken using the simplified substrate **41** (Table 2). Initially, attempts were made to reduce **41** into the corresponding aniline **42**, with a view to its subsequent elaboration, e.g., via diazotization. However, a variety of reduction conditions resulted either in no observable reaction (Table 2, entries 1 and 2), or else in defluorination at the benzyl position (Table 2, entry 3). The latter proceess is precedented [41]. Since none of the reductions to arylamines were successful, an alternative approach was investigated in which the nitroarene group would be converted into the corresponding acetanilide **43**. If this approach were successful, it was envisaged that the acetanilide **43** could be directly oxidised to carboxylic acid **44**, thereby bypassing any diazotization process. Hydrogenation of **41** with 10% Pd/C in the presence of acetic anhydride allowed the isolation of acetanilide **43** in moderate yields (Table 2, entries 4−6). It was found that the acetic anhydride solvent needed to be freshly distilled in every case in order for the reaction to be successful. The reaction duration was another significant determinant of the yield of **43** (Table 2, entries 4−6), since the over-reduced (i.e., benzylic defluorination) product was still produced in varying amounts. The subsequent oxidation of **43** was successfully achieved using sodium metaperiodate and ruthenium chloride (Table 2) [39–40], with the desired carboxylic acid **44** being obtained in 31% yield.

**Table 2:** Model studies that informed the final steps of the synthesis.

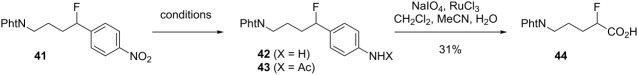		

Entry	Conditions	Outcome

1	Na_2_S_2_O_4_, aq HCl, rt, 20 h	no reaction
2	Na_2_S_2_O_4_, HCl, ethanol, reflux, 4 h	no reaction
3	Pd/C, ammonium formate, THF, 5 h	defluorination of **41** observed by ^1^H and ^19^F NMR analysis of crude reaction mixture
4	H_2_, 10% Pd/C, Ac_2_O, 3 h	**43** (38%)
5	H_2_, 10% Pd/C, Ac_2_O, 5 h	**43** (58%)
6	H_2_, 10% Pd/C, Ac_2_O, 18 h	**43** (21%)

Having established the conditions necessary for the conversion of the nitroaryl group in model system **41** (Table 2), the procedure could now be applied to the trifluoroalkanes **40a**,**b** (Scheme 4). Thus, compound **40a** was dissolved in freshly distilled acetic anhydride and subjected to hydrogenation over Pd/C (Scheme 4). The reaction was monitored by TLC at short time intervals in order to avoid over-reduction. The starting material was consumed within 5 h, but the expected acetanilide product (see Supporting Information File 1) was accompanied by varying quantities of a side-product that was tentatively identified either as an alternative rotamer of the acetanilide, or the corresponding imide (i.e., ArNAc_2_, see Supporting Information File 1). Although the formation of this imide would be unexpected, it was reasoned that it might still be a suitable substrate for the subsequent oxidation reaction. Accordingly, the product of the hydrogenation reaction was next treated with sodium metaperiodate and ruthenium trichloride (Scheme 4), and gratifyingly this delivered the desired trifluorinated carboxylic acid (see Supporting Information File 1) in moderate yield. Finally, the pthalimide group was removed with hydrazine to give the target amino acid **6a** (Scheme 4). The modest overall yield for this three-step sequence can be partially attributed to the challenge of purifying the penultimate and final compounds, which were of low molecular weight and very polar. Nevertheless, the first synthesis of a δ-amino acid containing three vicinal fluorines on the backbone had been successfully completed. The all-*syn* target **6b** was then obtained in a similar fashion from **40b** (Scheme 4).

**Figure 2:**
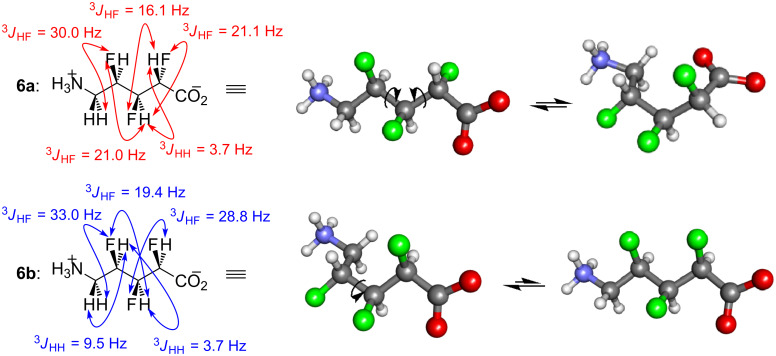
Selected *J* values and the inferred molecular conformations of **6a** and **6b**.

The ^1^H and ^19^F NMR spectra of **6a** and **6b** were simulated (see Supporting Information File 1) in order to measure the spin–spin coupling constants and thereby gain information on the solution-state conformations (Figure 2). For **6a**, the observed *J* values about the Cα–Cβ and Cβ–Cγ bonds are intermediate in magnitude [42], suggesting that conformational averaging is occurring about both of these bonds. In contrast, the *J* values about the Cγ–Cδ bond of **6a** fall clearly into either *gauche* or *anti* ranges [42], suggesting that this part of the molecule is relatively rigid in solution. Overall, the pattern of large, small and intermediate *J* values is consistent with two major conformations of **6a** existing in equilibrium (Figure 2). The first conformer (left) has an extended zigzag structure. This matches the geometry that was observed in the X-ray crystal structure for the *anti*,*syn-*trifluoroalkane **40a** [17]. The second conformer (right) has a bent shape which provides *gauche* alignments between all pairs of vicinal C–F and C–N bonds, whilst avoiding any 1,3-dipolar repulsions [11–12,43].

The observed *J* values for the all-*syn* trifluoro amino acid **6b** also allowed its solution conformation to be deduced (Figure 2). The *J* values about the Cα–Cβ and Cγ–Cδ bonds of **6b** mostly fall clearly into *gauche* or *anti* ranges, suggesting that these segments of the molecule are relatively rigid in solution. In contrast, the *J* values about the Cβ–Cγ bond of **6b** are more intermediate in magnitude (e.g., ^3^*J*_HH_ = 3.7 Hz), suggesting that conformational averaging could be occurring about this bond. Overall, the pattern of large, small and intermediate *J* values is consistent with two conformations of **6b** existing in equilibrium (Figure 2). The first conformer (left) has a bent structure. This provides *gauche* alignments between all pairs of vicinal C–F and C–N bonds, whilst avoiding 1,3-dipolar repulsion [11–12,43]. The second suggested conformer of **6b** (right) has an extended zigzag structure. This geometry is counterintuitive, because although it provides *gauche* alignments between all pairs of vicinal C–F and C–N bonds, it includes an unfavourable parallel alignment of the Cα–F and Cγ–F bonds. The extended conformer of **6b** may be a minor contributor only.

## Conclusion

Full details have been presented of the efforts that were required to identify and optimise a synthetic route towards the δ-amino acids **6a** and **6b**, molecules which contain three vicinal C–F bonds positioned stereospecifically along the backbone. Several synthetic approaches towards these challenging targets were investigated, involving both electrophilic and nucleophilic fluorination chemistry. The ultimately successful approach involved a modification of O’Hagan’s method [34], in which a stereochemically-defined epoxy alcohol precursor underwent three sequential nucleophilic deoxyfluorination reactions. The solution-state geometries of amino acids **6a** and **6b** were probed through qualitative NMR *J*-based analyses, revealing that **6a** and **6b** exhibit distinct conformational behaviour. This suggests that these fluorinated backbone-extended amino acids might enjoy future applications, for example as shape-controlled building blocks for incorporation into bioactive peptides [16].

## Supporting Information

File 1 Synthetic procedures and characterisation data of intermediated, NMR spectra and NMR simulations for **6a**,**b**.
