# Supplementary material for: Homologated amino acids with three vicinal fluorines positioned along the backbone: development of a stereoselective synthesis
Source: Beilstein J Org Chem. 2017 Nov 1;13:2316–25. doi: 10.3762/bjoc.13.228 (PMC5687047; doi:10.3762/bjoc.13.228)
Supplement: File 1 — Synthetic procedures and characterisation data of intermediated, NMR spectra and NMR simulations for 6a,b. [file Beilstein_J_Org_Chem-13-2316-s001.pdf]

Supporting Information

for

Homologated amino acids with three vicinal fluorines positioned along the backbone: development of a stereoselective synthesis

Raju Cheerlavancha[1], Ahmed Ahmed[1], Yun Cheuk Leung[1], Aggie Lawer[1], Qing-Quan Liu[2], Marina Cagnes[3], Hee-Chan Jang[3], Xiang-Guo Hu[2] and Luke Hunter*[1]

Address: [1]School of Chemistry, The University of New South Wales, Sydney NSW 2052, Australia, [2]National Engineering Research Center for Carbohydrate Synthesis, Jiangxi Normal University, Nanchang, China and [3]School of Chemistry, The University of Sydney, Sydney NSW 2006, Australia

Email: Luke Hunter - l.hunter@unsw.edu.au

*Corresponding author

Synthetic procedures and characterisation data of intermediated, NMR spectra and NMR simulations for 6a,b

Contents:

Synthetic procedures and characterization of intermediates ………………………………………… S-2

Spectra of novel compounds ……………………………………………………………………… S-28

NMR simulations for **6a**,**b** ……………………………………………………………………… S-68

References …………………………………………………………………………………………… S-72

Synthetic procedures and characterization of intermediates

Amino acid 6a

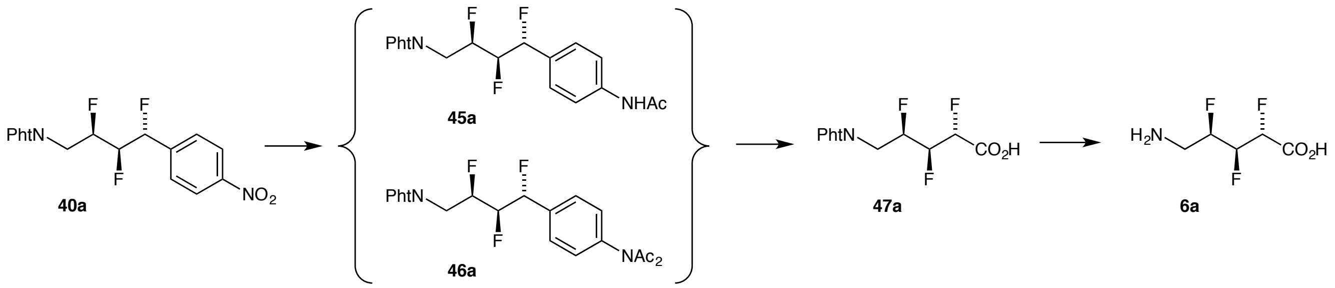

Pd/C (~6 mg) was added to a solution of **40a** (12 mg, 0.031 mmol) in dry acetic anhydride (~3 mL). The mixture was stirred under H_2 atmosphere for 5 h. The reaction mixture was filtered through celite (ethyl acetate wash) and concentrated under reduced pressure. The crude product was purified by flash chromatography (DCM → DCM / EtOAc 17:5) to give imide **46a** as an off-white solid (11.2 mg, 86%); **[α]D** +44.4 (*c* 0.45, $CHCl_3$); **IR** (neat) ν_{max} (cm^{-1}) 2931, 1796, 1609, 1509, 1390, 1172, 1189, 1101, 834; **^{1}H NMR** (300 MHz, $CDCl_3$) δ 7.93–7.74 (m, 4H, phthalimide), 7.54 (d, *J* = 8.6 Hz, 2H, *p*-acetyl phenyl), 7.48 (d, *J* = 8.6 Hz, 2H, *p*-acetyl phenyl), 5.64 (ddd, *J* = 45.0, 8.7, 4.5 Hz, 1H, CHF–CHF–CHF–CH_2), 5.19 (dddddd, *J* = 46.5, 27.2, 25.8, 8.4, 3.9, 1.0 Hz, 1H, CHF–CH_2), 4.64 (ddddd, *J* = 44.8, 25.8, 8.7, 4.3, 1.0 Hz, 1H, CHF–CHF–CH_2), 4.32 (ddd, *J* = 14.5, 8.4, 3.9 Hz, 1H, CHF–CH_2), 3.96 (ddd, *J* = 27.7, 14.5, 3.9 Hz, 1H, CHF–CH_2), 2.24 (s, 3H, CH_3), 2.13 (s, 3H, CH_3); **^{13}C{^{1}H} NMR** (75 MHz, $CDCl_3$) δ 167.8, 140.2, 134.3, 131.8, 127.6, 123.6, 91.7–85.4 (m, 3 × CHF), 38.0 (m, CH_2CHF), 29.6 [1 × C=O signal obscured]; **^{19}F NMR** (282 MHz, $CDCl_3$) δ −187.9 (m, 1F, *p*-acetyl phenyl–CHF), −207.0 (m, 1F, CHF–CH_2), −211.0 (m, 1F, CHF–CHF–CHF); **^{19}F{^{1}H} NMR** (282 MHz, $CDCl_3$) δ −187.9 (m, 1F, *p*-acetyl phenyl–CHF), −207.0 (m, CHF–CH_2), −211.0 (m, 1F, CHF–CHF–CHF).

To a stirred mixture of imide **46a** (10 mg, 0.023 mmol), dichloromethane (0.6 mL), acetonitrile (0.6 mL) and water (0.72 mL) was added sodium metaperiodate (98 mg, 0.46 mmol) followed by ruthenium chloride hydrate (~3 mg). The resulting mixture was stirred at room temperature for 5 days. The mixture was filtered through celite (ethyl acetate wash) and the filtrate was concentrated *in vacuo*. The crude product was subjected to flash chromatography (98:0:2→80:18:2 $CHCl_3$/MeOH/AcOH) to give carboxylic acid **47a**[1] as a brown oil (3.9 mg, 46%); **[α]D** +65 (*c* 0.15, $CHCl_3$); **IR** (neat) ν_{max} (cm^{-1}) 3381, 2917, 2942, 2368, 1716, 1632, 1393, 1032; **^{1}H NMR** (400 MHz, CD_3CN) δ 7.73–7.46 (m,

4H, phthalimide), 5.17–5.50 (m, 3H, C$\underline{H}$F–C$\underline{H}$F–C$\underline{H}$F), 4.16–3.99 (m, 2H, CHF–C$\underline{H}_2$); **^{19}F NMR** (282 MHz, CD_3CN) δ −185.1 (m, 1F, CHF–CH_2), −207.3 (m, 1F, CH$\underline{F}$–CHF–CHF), −212.5 (m, 1F, CHF–CH$\underline{F}$–CHF); **^{19}F{^{1}H} NMR** (282 MHz, CD_3CN) δ −198.5 (m, 1F, CHF–CH_2), −201.5 (m, 1F, CH$\underline{F}$–CHF–CHF), −207.1 (m, 1F, CHF–CH$\underline{F}$–CHF); **HRMS** (ESI, +ve) $C_{13}H_{10}F_3NO_4Na^+$ [MNa^+] requires *m/z* 324.0455, found 324.0449.

A solution of carboxylic acid **47a** (4.0 mg, 0.013 mmol) and hydrazine hydrate (1.3 μL, 0.02 mmol) in ethanol (1.0 mL) was heated at reflux for 5 h, then cooled and concentrated under reduced pressure. The crude compound was triturated with 4M aq. HCl (1 mL × 3) and filtered through celite. The filtrate was concentrated, then triturated in water (1 × 2 mL) and filtered again. The filtrate was concentrated under reduced pressure and purified by preparative HPLC to give **6a** as a sticky solid (0.8 mg, 29%); **[α]$_D$** −125 (c 0.08, H_2O); **IR** (neat) ν_{max} (cm^{-1}) 3564, 2964, 1738, 1613, 1505, 1415, 1215, 1115, 1081, 1048; **^{1}H NMR** (600 MHz, D_2O) δ 5.20 (ddddd, *J* = 49.3, 21.0, 16.0, 11.0, 3.6 Hz, 1H, *α*-CHF), 5.14 (ddddd, *J* = 45.0, 15.0, 16.0, 7.0, 3.8, Hz, 1H, *γ*-CHF), 5.12 (ddddd, *J* = 47.0, 21.0, 7.0, 3.8, 3.6 Hz, 1H, *β*-CHF), 3.48 (ddd, *J* =17.8, 7.0, 3.5, Hz, 1H, δ-CHH), 3.40 (ddd, *J* = 30.4, 18.2, 3.5, Hz, 1H, *δ*-CHH); **^{19}F NMR** (282 MHz, D_2O) δ –194.1 (m, 1F, CH$\underline{F}$–CH_2), –203.6 (m, 1F, CHF–CH$\underline{F}$–CHF), –204.9 (m, 1F, CH$\underline{F}$–CHF–CHF); **^{19}F{^{1}H} NMR** (282 MHz, D_2O) δ–194.2 (m, 1F, CH$\underline{F}$–CH_2), –203.6 (m, 1F, CHF–CH$\underline{F}$–CHF), –204.9 (m, 1F, CH$\underline{F}$–CHF–CHF); **HRMS** (ESI, +ve) $C_5H_9NO_2F_3^+$ [MH^+] requires *m/z* 172.0580, found 172.0577.

Amino acid 6b

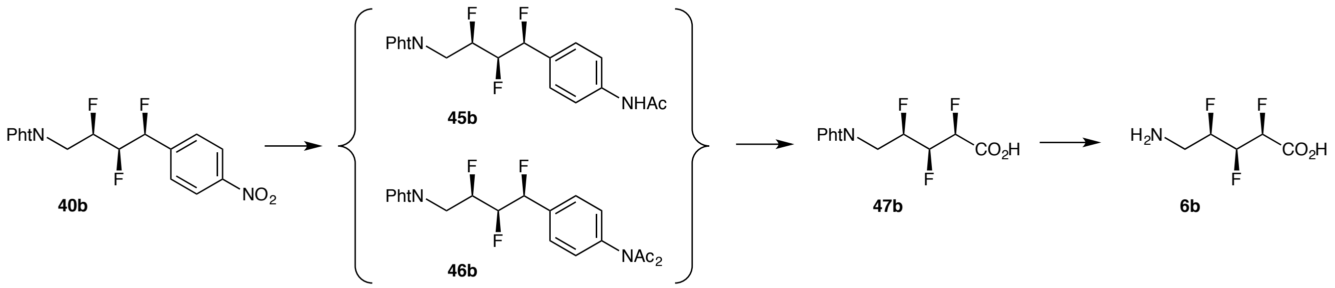

Pd/C (~15 mg) was added to a mixture of **40b** (30.0 mg, 0.08 mmol) in acetic anhydride (~6 mL). The mixture was stirred under H_2 atmosphere for 5 h. The reaction mixture was filtered through celite (ethyl acetate wash) and concentrated under reduced pressure. The crude product was purified by flash chromatography (dichloromethane → dichloromethane / ethyl acetate 17:3) to provide **45b** as a white

solid (28 mg, 83%) and a side-product (4.0 mg) that was tentatively identified either as an alternative amide rotamer of **45b**, or imide **46b**.

Data for **45b**: **m.p.** 175−178 °C; **[α]$_D$** −31.2 (*c* 0.01, $CHCl_3$); **^{1}H NMR** (300 MHz, $CDCl_3$) δ 7.86−7.71 (m, 4H, phthalimide), 7.55 (d, *J* = 8.5 Hz, 2H, *p*-nitrophenyl), 7.35 (d, *J* = 8.5 Hz, 2H, *p*-nitrophenyl), 5.74 (ddd, *J* = 47.6, 13.3, 7.4 Hz, 1H, *p*-nitrophenyl−C<u>H</u>F), 4.78 (ddddd, *J* = 48.1, 26.3, 14.4, 7.4, 1.8 Hz, 1H, CHF−C<u>H</u>F−CHF), 4.47 (ddddd, *J* = 47.6, 23.6, 8.8, 3.2, 1.8 Hz, 1H, C<u>H</u>F−CH_2), 4.31 (ddd, *J* = 15.2, 14.5, 8.8 Hz, 1H, C<u>H</u>H), 3.86 (ddd, *J* = 30.2, 15.2, 3.2 Hz, 1H, CH<u>H</u>), 2.21 (s, 3H, CH_3); **^{19}F NMR** (282 MHz, $CDCl_3$) δ −185.8 (ddd, *J* = 47.6, 16.9, 14.4 Hz, 1F, *p*-nitrophenyl−CHF), −204.7 (ddddd, *J* = 47.3, 30.2, 26.3, 14.5, 10.3 Hz, 1F, CHF−CH_2), −205.6 (ddddd, *J* = 48.1, 23.6, 16.9, 13.3, 10.3 Hz, 1F, CHF−CH<u>F</u>−CHF); **^{19}F{^{1}H} NMR** (282 MHz, $CDCl_3$) δ −185.8 (d, *J* = 16.9 Hz, 1F, *p*-nitrophenyl−CHF), −204.7 (d, *J* = 10.3 Hz, 1F, CHF−CH_2), −205.6 (dd, *J* = 16.9, 10.3 Hz, 1F, CHF−CH<u>F</u>−CHF).

Data for **46b**: **[α]$_D$** −83 (*c* 0.12, $CHCl_3$); **^{1}H NMR** (300 MHz, $CDCl_3$) δ 7.87−7.73 (m, 4H, phthalimide), 7.52 (d, *J* = 8.3 Hz, 2H, *p*-nitrophenyl), 7.46 (d, *J* = 8.3 Hz, 2H, *p*-nitrophenyl), 5.83 (ddd, *J* = 46.9, 15.7, 6.7 Hz, 1H, *p*-nitrophenyl−C<u>H</u>F), 4.79 (ddddd, *J* = 47.4, 24.9, 15.5, 6.7, 2.6 Hz, 1H, CHF−C<u>H</u>F−CHF), 4.57 (ddddd, *J* = 47.6, 23.6, 8.8, 3.4, 2.6 Hz, 1H, C<u>H</u>F−CH_2), 4.30 (ddd, *J* = 16.3, 15.3, 8.8 Hz, 1H, C<u>H</u>H), 3.92 (ddd, *J* = 29.2, 15.3, 3.4 Hz, 1H, CH<u>H</u>), 2.23 (s, 3H, CH_3); **^{19}F NMR** (282 MHz, $CDCl_3$) δ −188.6 (m, 1F, *p*-nitrophenyl−CHF), −203.7 (ddddd, *J* = 47.6, 29.2, 24.9, 16.3, 10.6 Hz, 1F, CHF−CH_2), −206.0 (m, 1F, CHF−CH<u>F</u>−CHF); **^{19}F{^{1}H} NMR** (282 MHz, $CDCl_3$) δ −188.6 (m, 1F, *p*-nitrophenyl−CHF), −203.7 (d, *J* = 10.6 Hz, 1F, CHF−CH_2).

To a stirred mixture of **45b** (15.0 mg, 0.034 mmol), dichloromethane (0.5 mL), acetonitrile (0.5 mL) and water (0.62 mL) was added sodium metaperiodate (147 mg, 0.7 mmol) followed by ruthenium chloride hydrate (~6 mg). The resulting mixture was stirred at room temperature for 5 days. The mixture was filtered through celite (ethyl acetate wash) and the filtrate was concentrated *in vacuo*. The crude product was subjected to flash chromatography (98:0:2→80:18:2 $CHCl_3$/MeOH/AcOH) to give carboxylic acid **51**[1] as a white semisolid (6.0 mg, 60%); **[α]$_D$** +47.6 (*c* 0.02, $CHCl_3$); **^{1}H NMR** (300 MHz, CD_3CN) δ 7.88−7.71 (m, 4H, phthalimide), 5.43 (ddd, *J* = 47.1, 28.9, 2.6 Hz, 1H, *p*-nitrophenyl−C<u>H</u>F), 5.23−5.03 (m, 2H, CHF−C<u>H</u>F−C<u>H</u>F), 4.09 (ddd, *J* = 15.0, 12.6, 6.2 Hz, 1H, C<u>H</u>H), 4.03 (dd, *J* = 23.5, 15.0, 4.1 Hz, 1H, CH<u>H</u>); **^{19}F NMR** (282 MHz, CD_3CN) δ −203.2 (dddddd, *J* = 46.8, 23.5, 12.6, 11.6, n/d, 8.8 Hz, 1F, CHF−CH_2), −207.8 (dddd, *J* = 47.1, 24.7, 9.4, 8.8 Hz, 1F, *p*-

nitrophenyl–CHF), –210.9 (ddddd, J = 46.3, 28.9, 17.6, 11.6, 9.4 Hz, 1F, CHF–CH$\underline{F}$–CHF); **^{19}F{^{1}H} NMR** (282 MHz, CD_3CN) δ –203.2 (dd, J = 11.6, 8.8 Hz, 1F, CHF–CH_2), –207.8 (dd, J = 9.4, 8.8 Hz, 1F, *p*-nitrophenyl–CHF), –210.9 (dd, J = 11.6, 9.4 Hz, 1F, CHF–CH$\underline{F}$–CHF); **HRMS** (ESI, +ve) $C_{13}H_{10}F_3NO_4Na^+$ [MNa^+] requires *m*/*z* 324.0455, found 324.0449.

A solution of carboxylic acid **47b** (4.0 mg, 0.013 mmol) and hydrazine hydrate (1.3 µL, 0.02 mmol) in ethanol (1.0 mL) was heated at reflux overnight, then cooled and concentrated under reduced pressure. The crude compound was triturated with 4 M aq. HCl (1 mL × 3) and filtered through celite. The filtrate was concentrated, then triturated in water (1 × 2 mL) and filtered again. The filtrate was concentrated under reduced pressure and purified by preparative HPLC to give **6b** as a sticky solid (1.2 mg, 41%); **[α]$_D$** +8.3 (c 0.01, H_2O); **IR** (neat) ν_{max} (cm^{-1}) 3564, 2964, 1738, 1613, 1505, 1415, 1215, 1115, 1081, 1048; **^{1}H NMR** (600 MHz, D_2O) δ 5.17 (ddddd, J = 45.2, 20.0, 9.0, 3.9, 3.8 Hz, 1H, γ-CHF), 5.08 (ddddd, J = 47.0, 28.8, 21.0, 11.0, 2.9, Hz, 1H, α-CHF), 5.06 (ddddd, J = 43.2, 21.0, 3.9, 3.4, 2.9 Hz, 1H, β-CHF), 3.44 (ddd, J =16.0, 14.2, 9.6, Hz, 1H, δ-C$\underline{H}$H), 3.40 (ddd, J = 30.4, 14.2, 3.9, Hz, 1H, δ-CH$\underline{H}$); **^{13}C{^{1}H} NMR** (100 MHz, D_2O) δ 172.5, 88.0, 39.6; **^{19}F NMR** (282 MHz, D_2O) δ –198.6 (m, 1F), –204.0 (m, 1F), –209.1 (m, 1F); **^{19}F{^{1}H} NMR** (282 MHz, D2O) δ –198.5 (dd, J = 10.5, 8.65 Hz, 1F), –203.9 (d, J = 11.5, 9.3 Hz, 1F); –209.1 (d, J = 11.6, 9.3 Hz, 1F); **HRMS** (ESI, +ve) $C_5H_9NO_2F_3^+$ [MH^+] requires *m*/*z* 172.0580, found 172.0577.

Fluoroalcohol 11

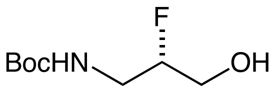

A solution of aldehyde **7b**[2] (97.3 mg, 0.56 mmol) and (*S*)-**10**[3] (3.2 mg, 0.004 mmol) in MTBE (1 mL) was stirred at room temperature for 15 min. NFSI (121.5 mg, 0.38 mmol) was added, and the mixture was stirred at room temperature for 2 h. Pentane (2 mL) was added and the precipitate was filtered off, the filtrate was concentrated under *vacuo*. The desired product was dissolved in MeOH (3 mL) and $NaBH_4$ (30.0 mg, 0.8 mmol) was added. The reaction was stirred for 1 h at room temperature and then quenched with $KHSO_4$ (1 M, 3 mL), the aqueous layer was extracted with EtOAc (4 × 5 mL). The organic layers were collected and dried over $MgSO_4$ and concentrated in *vacuo*. The crude product was subjected to flash chromatography and eluted with 5:2 hexane/EtOAc to give the

title compound as a colourless oil (21.8 mg, 20%); **R$_f$** 0.51 (3:1:1 hexane/EtOAc/DCM); **^{1}H NMR** (300 MHz, $CDCl_3$) δ 4.86 (br, 1H, NH), 4.55 (m, 1H, CHF), 3.70 (m, 2H, CH_2O), 3.47 (m, 2H, CH_2N), 3.02 (t, *J* = 6.8, 13.8 Hz, 1H, OH), 1.45 (s, 9H, $3CH_3$); **^{13}C{^{1}H} NMR** (75 MHz, $CDCl_3$) δ 149.9, 147.2, 139.1, 127.0, 123.6, 116.7, 4.4, 60.6, 21.0, 14.1; **^{19}F NMR** (282 MHz, CDCl3) δ –195.84 (m, 1F, CHF); ***ee*** >99% from Mosher analysis.[4]

Difluoroalcohol 12

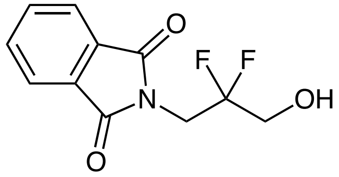

A solution of **7a** (30.1 mg, 0.15 mmol, 1.5 eq) and (*S*)-**10** (0.6 mg, 0.001 mmol, 0.01 eq) was stirred at room temperature in anhydrous THF (2 mL) for 30 min before adding NFSI (31.2 mg, 0.01 mmol, 1 eq). The reaction mixture was then stirred at room temperature for 6 h, then pentane was added and the precipitate was filtered off. The residue was concentrated and then dissolved in MeOH (2 mL), $NaBH_4$ (1 mmol) was added and stirred for 1 h at room temperature. The reaction was quenched with (1M, 2 mL) aqueous solution of $KHSO_4$, (2 mL) water was added and the aqueous layer was extracted with EtOAc (3 × 5 mL), the organic layers was collected and dried over $MgSO_4$ and evaporated in *vacuo*. The crude compound was subjected to silica gel column (98%–90% hexane/EtOAc) to give the title compound as colourless oil; **^{1}H NMR** (300 MHz, CD_3OD) 7.71 (m, 4H, Ar), 3.85 (m, 4H, $2CH_2$). **^{19}F NMR** (282 MHz, CDCl3) δ –110.83 (m, 1F, CHF), –113.23 (m, 1F, CHF).

Bis-nitrile 14

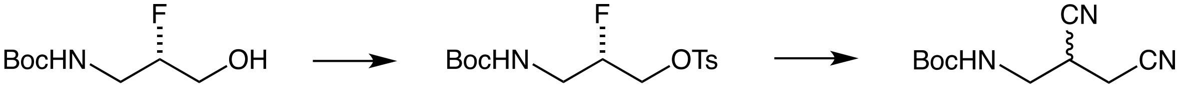

11 **48** **14**

A mixture of Et_3N (0.2 mL), TsCl (23.1 mg, 1.2 mmol) and DMAP (1.0 mg, 0.1 mmol) were stirred in anhydrous DCM (2 mL) at room temperature. Then alchol **11** (15.0 mg, 1 mmol) was added in one

portion and continued stirring at room temperature for 3 h. The crude mixture was subjected to column chromatography (5%−15% EtOAc/hexane) to give tosylate **48** as a colourless oil (13.2 mg, 40%); **R_f** 0.53 (2:1 hexane/EtOAc); **[α]$_D$** 0.6 (*c* 0.1, $CHCl_3$); **IR** (neat) ν_{max} (cm^{-1}) 3331.9, 29860.8, 2924.5, 2115.6, 1682.8, 1363.1, 1174.8; **^{1}H NMR** (300 MHz, $CDCl_3$) δ 7.80−7.34 (m, 4H, Ar), 4.79−4.663 (m, 1H, CHF), 4.26−4.07 (m, 2H, CH_2CO), 3.44−3.30 (m, 2H, CH_2CN), 2.45 (s, 3H, CH_3), 1.42 (s, 9H, $3CH_3$); **$^{13}C\{^1H\}$ NMR** (75 MHz, $CDCl_3$) δ 155.9, 145.3, 132.5, 130.1, 128.1, 89.2, 80.2, 68.7, 41.0, 28.4, 22.7; **^{19}F NMR** (282 MHz, CDCl3) δ –193.4 (m, 1F, CHF); **HRMS** (ESI, +ve) $C_{11}H_9NO_4Na^+$ [M+Na+] requires *m*/*z* 370.1094, found 370.1087.

Tosylate **48** (13.0 mg, 0.04 mmol) was stirred in DMSO (2 mL) at 80 °C and then potassium cyanide (9.1 mg, 0.14 mmol) was added and continued stirring for 4 h at 80 °C. The reaction mixture was allowed to cool to room temperature and EtOAc (3 mL) and water (3 mL) were added. The organic layer was separated and the aqueous layer was extracted with EtOAc (3 × 3 mL). The crude mixture was subjected to column chromatography (15% EtOAc/hexane) to give bis-nitrile **14** as a white solid (16.1 mg, 40%); **[α]$_D$** 0.0 (*c* 0.2, $CHCl_3$); **R_f** 0.56 (3:2 hexane/EtOAc); **IR** (neat) ν_{max} (cm^{-1}) 3381, 2971, 2249, 1683, 1513; **^{1}H NMR** (300 MHz, $CDCl_3$) δ 5.12 (br s, 1H, NH), 3.50 (td, *J* = 6.0, 2.2 Hz, 2H, $C\underline{H}_2CNH$), 3.30 (m, 1H, $CH_2C\underline{H}CH_2$), 2.78 (dd, *J* = 6.0, 2.8 Hz, 2H, CH_2CN), 1.47 (s, 9H, 3 × CH_3); **$^{13}C\{^1H\}$ NMR** (75 MHz, $CDCl_3$) δ 156.0, 117.9, 115.5, 81.1, 41.1, 29.7, 28.4, 16.7; **HRMS** (ESI, +ve) $C_{11}H_9NO_4Na^+$ [M+Na+] requires *m*/*z* 232.1397, found 232.1029. Compound **14** was also analysed by chiral HPLC, but it was not possible to observe separate enantiomers.

β-Fluoroaldehyde 17

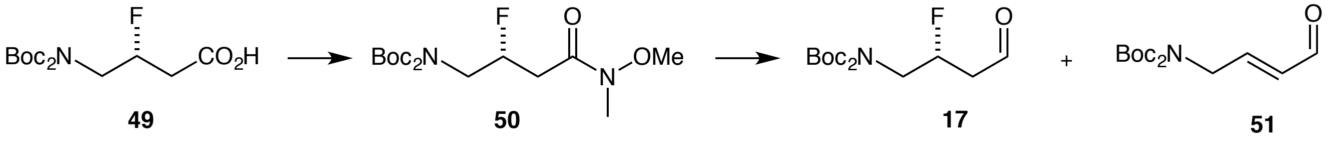

To a vigorously stirring solution of **49**[5] (83.1 mg, 0.258 mmol) in CH_2Cl_2 (40 mL) at 0 °C was added HOBt (52.3 mg, 0.387 mmol) and DIC (48.9 mg, 0.387 mmol). This solution was allowed to warm to room temperature over 1.5 h before *N*,*O*-dimethylhydroxylamine hydrochloride (37.8 mg, 0.387 mmol) was added. The resulting suspension was stirred for 13 h at room temperature before it was diluted with 10 mL of 1 *N* aqueous HCl. The layers were separated and the aqueous layer was washed with CH_2Cl_2 (10 mL). The combined organic layers were washed with H_2O (10 mL) and a 1 *N* aqueous solution of

NaOH (10 mL), dried with $MgSO_4$, and concentrated in *vacuo* to afford Weinreb amide **50** as a colorless oil (47.9 mg, 51%), which crystallized after standing at room temperature; **[α]$_D$** −15.6 (*c* 0.60, $CHCl_3$); **m.p.** 53 °C, **IR** (neat) ν_{max} (cm^{-1}) 2921, 2854, 2361, 2046, 2021, 1710, 1358, 1231, 1122, **^{1}H NMR** (300 MHz, $CDCl_3$) δ 5.06–5.30 (m, 1H, CHF), 3.73–4.08 (m, 2H, $CFCH_2N$), 3.69 (s, 3H, OCH_3), 3.19 (s, NCH_3), 2.52–3.03 (m, 2H, CH_2CO), 1.50 (s, 18H, 6 × CH_3); **^{13}C{^{1}H} NMR** (75 MHz, $CDCl_3$) δ 173.0 (CON), 152.6 (NCO_2), 90.0 (*J* = 174.0 Hz, CHF), 82.9 ($C(CH_3)_3$), 61.4 ($NOCH_3$), 49.2 (d, *J* = 23.2 Hz, CH_2N), 35.6 (d, *J* = 22.9 Hz, $CO\underline{C}H_2CF$), 32.2 (NCH_3), 28.1 (6 × CH_3); **^{19}F NMR** (280 MHz, $CDCl_3$): δ −185.9 (m, 1F); **^{19}F{^{1}H} NMR** (280 MHz, $CDCl_3$) δ −185.9 (s, 1F); **HRMS** (ESI, +ve) $C_{16}H_{29}FN_2O_6Na^+$ [$M+Na^+$] requires *m/z* 387.1902, found 387.1889.

To a stirred solution of **50** (12.6 mg, 0.0345 mmol) in THF (4 mL) at −78 °C was added DIBAL-H (0.0379 mmol). The reaction mixture was stirred for 30 min at −78 °C before being quenched with 1.0M aq. HCl solution. The mixture was extracted with EtOAc, and the organic layer was dried with Na_2SO_4 and concentrated in *vacuo* to afford a colorless oil comprising an inseparable mixture of **17** (major) and **51** (minor). Data for **17**: **^{1}H NMR** (300 MHz, $CDCl_3$) δ 9.79 (s, 1H, CHO), 5.34–5.10 (m, 1H, CHF), 4.07–3.69 (m, 2H, $CFCH_2N$), 2.90–2.61 (m, 2H, CH_2CO), 1.50 (s, 18H, 6 × CH_3).

An independent sample of **51** was prepared for comparison purposes, as follows: To a solution of **50** (41.2 mg, 0.113 mmol) in THF (3 mL) was added $LiAlH_4$ (12.8 mg, 0.338 mmol) at 0 °C. After stirring for 1 h at 0 °C, water (15 μL), 20% aq. NaOH (15 μL) and water (45 μL) were added successively. The mixture was stirred at room temperature for 1 h, after which the reaction was quenched by adding excess amount of saturated NH_4Cl aqueous solution, followed by extraction with diethyl ether. The organic phase was washed with brine and then dried over anhydrous Na_2SO_4. After the solution was filtered and the solvent was evaporated *in vacuo*, the residue was purified by flash chromatography to afford the title compound **18** as a colourless oil (3.1 mg, 9%); **^{1}H NMR** (300 MHz, $CDCl_3$) δ 9.57 (d, *J* = 7.80, 1H, CHO), 6.79 (dt, *J* = 15.8, 4.78, 1H, $C\underline{H}CHCHO$), 6.15 (ddt, *J* = 15.8, 7.80, 1.73, 1H, $C\underline{H}CHO$), 4.47 (dd, *J* = 4.78, 1.73, CH_2NH), 1.50 (s, 18H, 6xCH_3); **^{13}C{^{1}H} NMR** (75 MHz, $CDCl_3$) δ 193.2 (CHO), 152.6 (NCO_2), 152.0 ($C\underline{H}CHO$), 132.1 ($C\underline{H}CH_2N$), 83.3 ($\underline{C}(CH_3)_3$), 46.9 (CH_2N), 28.3 (3 × CH_3); **HRMS** (ESI, +ve) $C_{14}H_{23}NO_5Na^+$ [$M+Na^+$] requires *m/z* 308.1474, found 308.1470.

β-Fluoroaldehyde 18

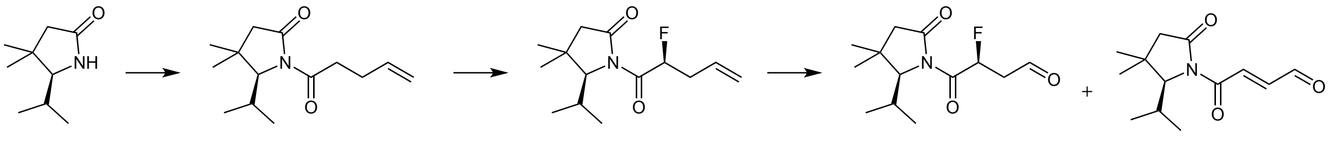

52 53 54 18 55

Oxalyl chloride (0.93mL, 11mmol) was added dropwise to a precooled (0 °C) solution of pent-4-enoic acid (1.0g, 10 mmol), dimethylformamide (10 μL), and dichloromethane (2.0 M). The reaction mixture was allowed to stir for 10 min at 0 °C, and then the solution was warmed to room temperature and stirred for an additional 45 min (or until bubbling stops). The solution was carefully concentrated in vacuo. In a separate flask, n-butyllithium (2.5 M in hexane, 4.2 mL, 10.5 mmol) was added dropwise to a precooled (−78 °C) solution of oxazolidinone **52** (1.57 g, 10 mmol) in THF (28 mL) under argon. The solution was stirred for 30 min at −78 °C. A solution of the crude acyl chloride in THF (15 mL total, 3 rinses) was added dropwise at −78 °C. After being stirred at −78 °C for 2 h, the reaction mixture was warmed to room temperature and stirred for 2 h. The reaction was quenched with saturated aqueous ammonium chloride. The layers were separated. The aqueous layer was extracted with ethyl acetate (3 × 20 mL). The combined organic layers were washed with brine, dried with sodium sulfate, and concentrated in vacuo. The resultant oil was purified by column chromatography (PE:EA=20:1) to give imide **53** as a colourless oil (1.237 g, 52%); $[2]_D$ +0.27 (*c* 0.075, $CHCl_3$); **IR** (neat) ν_{max} (cm^{-1}) 2973, 2928, 1776, 1701, 1393, 1374, 1364, 1315, 1278, 1220, 1171, 1122, 1070; ^{1}**H NMR** (400 MHz, $CDCl_3$): 5.84 (ddt, J = 6.4, 10.2, 16.7 Hz, 1H), 5.09 (dd, J = 1.6, 17.3 Hz, 1H), 5.01 (dd, J = 1.4, 10.3 Hz, 1H), 4.16 (d, J = 3.3 Hz, 1H), 3.12 (dt, J = 7.4, 16.7 Hz, 1H), 3.01 (dt, J = 7.2, 16.9 Hz, 1H), 2.15 (heptd, J = 3.2, 7.0 Hz, 1H), 1.52 (s, 3H), 1.39 (s, 3H), 1.02 (d, J = 7.0 Hz, 3H), 0.95 (d, J = 6.9 Hz, 3H); ^{13}**C{^{1}H} NMR** (100 MHz, $CDCl_3$): δ 173.0, 153.5, 136.8, 115.6, 82.8, 66.2 (d, J = 4.6 Hz), 34.7, 29.5, 28.7 (d, J = 19.9 Hz), 21.4 (d, J = 8.7 Hz), 17.0; **HRMS** (ESI): $[C_{13}H_{21}NO_3 + H]^+$ requires m/z 240.1594, found 240.1593.

In a flame-dried flask, $TiCl_4$ (1.0 M in CH_2Cl_2, 3.15 mL, 3.15 mmol) was added dropwise to the solution of *N*-acyloxazolidinone **53** (500 mg, 2.1 mmol) in CH_2Cl_2 (5.0 mL) at 0 °C under argon. The mixture was stirred for 5 min at 0 °C. Triethylamine (0.58 mL, 4.2 mmol) was added at 0 °C, and the solution was stirred for 30 min. *N*-Fluorosulfonimide (1.32 g, 4.2 mmol) was added in one portion at 0 °C (CAUTION: vigorous gas evolution). The mixture was warmed to 23 °C and stirred for 1 h. The reaction mixture was filtered through a 1.5 cm silica plug column, and the plug was rinsed with CH_2Cl_2

(10 mL) and EtOAc (50 mL). The combined filtrate was concentrated in vacuo. Analysis of the crude mixture of products by ^{1}H NMR spectroscopy showed the diastereoselectivity (dr >20:1) of the reaction. The crude residue was purified by flash column chromatography (PE:EA=15:1) to give **54** as a colourless oil (353 mg, 66%); **[2]$_D$** +0.31 (*c* 0.25, $CHCl_3$); **IR** (neat) ν_{max} (cm^{-1}) 2973, 2927, 1773, 1714, 1395, 1374, 1363, 1314, 1279, 1221, 1173, 1120, 1071; **^{1}H NMR** (400 MHz, $CDCl_3$) δ 6.10 (ddd, *J* = 4.5, 6.68, 49.3 Hz, 1H), 5.88 (ddt, *J* = 7.4, 9.61, 16.87, 1H), 5.20 (dd, *J* = 1.36, 17.2 Hz, 1H), 5.17 (dd, *J* = 1.56, 8.43 Hz, 1H), 4.12 (d, *J* = 3.15 Hz, 1H), 2.77-2.55 (m, 2H), 2.20 (heptd, *J* = 3.1, 6.9 Hz, 1H), 1.54 (s, 3H), 1.39 (s, 3H), 1.08 (d, *J* = 7.1 Hz, 3H), 0.98 (d, *J* = 6.9 Hz, 3H); **^{13}C{^{1}H} NMR** (100 MHz, $CDCl_3$) *δ* 169.4 (d, *J* = 22.8 Hz), 153.0, 131.1 (d, *J* = 3.9 Hz), 119.3, 88.1 (d, *J* = 180.4 Hz), 84.1, 66.9, 36.5 (d, *J* = 22.3 Hz), 29.1 (d, *J* = 59.7 Hz), 21.3 (d, *J* = 9.6 Hz), 16.9; **^{19}F NMR** (367 MHz, $CDCl_3$) *δ* –190.2 (dt, *J* = 49.7, 25.3 Hz, 1F); **^{19}F{^{1}H} NMR** (367 MHz, $CDCl_3$) *δ* –190.2 (s, 1F); **HRMS** (ESI): $[C_{13}H_{20}FNO_3 + H]^+$ requires *m/z* 258.1500, found 258.1495.

A solution of **54** (74 mg, 0.29 mmol) in CH_2Cl_2 (15 mL) was cooled to –78 °C and a stream of ozone was bubbled through the mixture until a deep blue color persisted. Oxygen was then bubbled through the solution for an additional 10 min to remove excess ozone. Triphenylphosphine (88.3 mg, 0.34 mmol) was added to the solution, which was then stirred and warmed to room temperature over 30 min. The mixture was then concentrated under reduced pressure, the products were purified by flash chromatography (PE:EA 12:1) on silica gel to give a small quantity of **18** (unstable, prone to elimination) and the α,β-unsaturated aldehyde **55** as a colourless oil (55 mg, 79%).

Data for **18**: **^{1}H NMR** (400 MHz, $CDCl_3$) *δ* 9.78 (t, *J* = 2.3 Hz, 1H), 6.43 (ddd, *J* = 5.1, 6.5, 47.3 Hz, 1H), 4.16 (d, *J* = 3.2 Hz, 1H), 3.21–2.96 (m, 2H), 2.20 (heptd, *J* = 3.3, 7.0 Hz, 1H), 1.55 (s, 3H), 1.44 (s, 3H), 1.06 (d, *J* = 7.1 Hz, 3H), 0.98 (d, *J* = 6.9 Hz, 3H); **^{19}F NMR** (367 MHz, $CDCl_3$) *δ* –189.9 (dddd, *J* = 2.8, 19.9, 24.2, 47.5 Hz, 1F); **^{19}F{^{1}H} NMR** (367 MHz, $CDCl_3$) *δ* –190.2 (s, 1F).

Data for **55**: **[2]$_D$** +0.43 (*c* 0.075, $CHCl_3$); **IR** (neat) ν_{max} (cm^{-1}) 2923, 2852, 1772, 1699, 1694, 1678, 1570, 1502, 1478, 1443, 1374, 1364, 1340, 1278, 1263, 1221, 1108, 1070; **^{1}H NMR** (400 MHz, $CDCl_3$) *δ* 9.84 (d, *J* = 7.9, 1H), 8.20 (d, *J* = 15.6 Hz, 1H), 7.06 (dd, *J* = 7.9, 15.6 Hz, 1H), 4.26 (d, *J* = 3.3 Hz, 1H), 2.21 (heptd, *J* = 3.3, 7.0 Hz, 1H), 1.57 (s, 3H), 1.44 (s, 3H), 1.07 (d, *J* = 7.0 Hz, 3H), 0.99 (d, *J* = 7.0 Hz, 3H); **^{13}C{^{1}H} NMR** (100 MHz, $CDCl_3$) *δ* 192.6, 164.1, 153.3, 140.7, 138.6, 83.8, 66.9, 29.7, 28.9, 21.4 (d, *J* = 9.1 Hz), 17.1; **HRMS** (ESI): $[C_{12}H_{17}NO_4 + H]^+$ calc. 240.12303, found 240.12270.

β-Fluoroalcohol 19

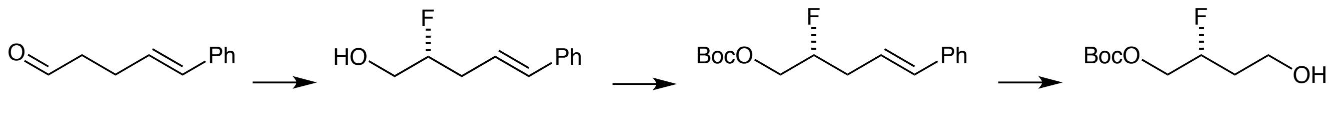

56 **57** **58** **19**

A solution of **56** (1.15 g, 7.2 mmol) and (*S*)-2-[bis-(3,5-bistrifluoromethyl-phenyl)-trimethylsilanyloxy-methyl]-pyrrolidine (215 mg, 0.36 mmol) in MTBE (20 mL) was stirred at r.t. for 10 min before the addition of *N*-fluorodibenzenesulfonimide (NFSI) (2.27 g, 7.2 mmol). The reaction mixture was then stirred for 12 h at r.t.. Pentane (10 mL) was added and the precipitate was filtered off. After dilution of the crude mixture with MeOH (15 mL), KBH_4 (583 mg, 10.8 mmol) was added at 0 °C. After 30 min the reaction was quenched with H_2O (5 mL). After standard aqueous workup, the products were purified by flash chromatography (PE:EA=5:1) on silica gel to give **57** as a colourless oil (967 mg, 75%); $\mathbf{[2]_D}$ –0.029 (*c* 0.62, $CHCl_3$); **IR** (neat) ν_{max} (cm^{-1}) 3378, 2932, 2363, 2340, 1598, 1494, 1449, 1248, 1030; **^{1}H NMR** (400 MHz, $CDCl_3$) δ 7.40−7.20 (m, 5H), 6.50 (d, *J* = 15.9 Hz, 1H), 6.19 (dt, *J* = 15.8 Hz, 7.3 Hz, 1H), 4.69 (dddt, *J* = 3.0, 6.1, 6.9, 49.1 Hz, 1H), 3.86−3.68 (m, 2H), 2.71−2.48 (m, 2H), 1.81 (br s, 1H); **^{13}C{^{1}H} NMR** (100 MHz, $CDCl_3$) δ 137.0, 133.5, 128.6, 127.5, 126.2, 123.7 (d, *J* = 7.0 Hz), 93.8 (d, *J* = 171.6 Hz), 64.3 (d, *J* = 23.7 Hz), 34.7 (d, *J* = 21.8 Hz); **^{19}F{^{1}H} NMR** (367 MHz, $CDCl_3$) δ –188.8 (s, 1F); **^{19}F NMR** (367 MHz, $CDCl_3$) δ –188.8 (dddt, *J* = 49.4, 25.9, 23.5, 17.9 Hz, 1F); **HRMS** (ESI, +ve) $[C_{11}H_{13}FO + H]^+$ requires *m*/*z* 181.1023, found 181.1017.

To a solution of **57** (60 mg, 0.34 mmol) in CH_2Cl_2 (3 mL) at 0 °C was added di-*tert*-butyl dicarbonate (74 mg, 0.34 mmol), Et_3N (40 mg, 0.4 mmol) and DMAP (0.4 mg, 0.0034 mmol). After 2 h of stirring, the reaction was quenched with H_2O, the organic layer was dried over anhydrous $MgSO_4$, filtered and concentrated. The residue was purified by flash chromatography (PE:EA=80:1) on silica gel to give **58** as a colorless oil (46 mg, 48%); $\mathbf{[2]_D}$ +0.072 (*c* 0.35, $CHCl_3$); **IR** (neat) ν_{max} (cm^{-1}) 2921, 2850, 2360, 1745, 1279, 1256, 1160, 1095; **^{1}H NMR** (400 MHz, $CDCl_3$) δ 7.38−7.20 (m, 5H), 6.51 (d, *J* = 16.5 Hz, 1H), 6.19 (dt, *J* = 7.2, 15.7 Hz, 1H), 4.81 (dddt, *J* = 3.1, 6.4, 6.4, 48.8 Hz, 1H), 4.26 (dd, *J* = 3.2, 7.4 Hz, 1H), 4.21 (dd, *J* = 3.2, 6.0 Hz, 1H) 2.71−2.50 (m, 2H), 1.49 (s, 9H); **^{13}C{^{1}H} NMR** (100 MHz, $CDCl_3$) δ 153.3, 136.9, 133.8, 128.6, 127.5, 126.2, 123.1, 123.0, 90.4 (d, *J* = 176.8 Hz), 67.7 (d, *J* = 23.7 Hz), 35.1 (d, *J* = 21.2 Hz), 27.7; **^{19}F{^{1}H} NMR** (367 MHz, $CDCl_3$) δ –186.7 (s, 1F); **^{19}F{^{1}H} NMR** (367 MHz, $CDCl_3$) δ –186.7 (m, 1F); **HRMS** (ESI, +ve) $[C_{16}H_{21}FO_3 + H]^+$ requires *m*/*z*. 281.1548, found 281.1547.

A solution of **58** (53 mg, 0.19 mmol) in CH_2Cl_2 (15 mL) was cooled to –78 °C and a stream of ozone was bubbled through the mixture until a deep blue color persisted. Oxygen was then bubbled through the solution for an additional 10 min to remove excess ozone. Dimethyl sulfide (0.2 mL, 3.8 mmol) was added to the solution, which was then stirred and warmed to room temperature over 1 h. The mixture was then concentrated under reduced pressure, the crude mixture with MeOH (4 mL), KBH_4 (102 mg, 1.9 mmol) was added at ambient temperature. After 30 min the reaction was quenched with H_2O (3 mL). After standard aqueous workup, the products were purified by flash chromatography (PE:EA=4:1) on silica gel to give **19** as a colorless oil (31 mg, 80%); **[2]$_D$** –0.026 (*c* 0.27, $CHCl_3$); **IR** (neat) ν_{max} (cm^{-1}) 3445, 2921, 2849, 2360, 1743, 1369, 1287, 1255, 1160, 1054; **^{1}H NMR** (400 MHz, $CDCl_3$) δ 4.93 (dddt, J = 2.9, 4.0, 6.7, 49.4 Hz, 1H), 4.32−4.14 (m, 2H), 3.83 (dd, J = 5.3, 6.7 Hz, 2H), 2.03−1.78 (m, 2H), 1.75 (br s, 1H), 1.50 (s, 9H); **^{13}C{^{1}H} NMR** (100 MHz, $CDCl_3$) δ 153.3, 89.1 (d, J = 171.7 Hz), 82.7, 68.1 (d, J = 22.3 Hz), 58.5 (d, J = 5.0 Hz), 33.9 (d, J = 19.7 Hz), 27.7; **^{19}F{^{1}H} NMR** (367 MHz, $CDCl_3$) δ –189.9 (s, 1F); **^{19}F NMR** (367 MHz, $CDCl_3$) δ –189.9 (dddt, J = 49.6 Hz, 38.0 Hz, 22.2 Hz, 15.8 Hz, 1F); **HRMS** (ESI, +ve) $[C_9H_{17}FO_4 + H]^+$ requires m/z 209.1184, found 209.1181.

β-Fluoroester 20

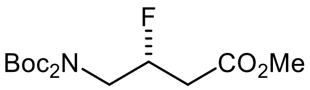

To a stirred mixture of compound **49**[5] (33.1 mg, 0.10 mmol), CH_2Cl_2 (3 mL), methanol (1 mL) and DMAP (10.2 mg, 0.082 mmol) at 0 °C was added DCC (23.3 mg, 0.113 mmol) over a 5 min period. After a further 5 min at 0 °C, the reaction mixture was stirred for 3 h at room temperature. The formed precipitate was removed by filtration, and the filtrate was washed with 0.5 *N* hydrochloric acid (2 × 10 mL) and saturated aqueous sodium bicarbonate solution (2 × 10 mL). During this procedure some additional precipitate formed, which was removed by filtration of both layers to facilitate their separation. The organic solution was dried over anhydrous sodium sulfate and concentrated *in vacuo*. The residue was purified by flash chromatography to afford the title compound as a colourless oil (21.5 mg, 62%); **[α]$_D$** –26.1 (*c* 0.80, $CHCl_3$); **IR** (neat) ν_{max} (cm^{-1}) 2979, 2358, 1743, 1435, 1367, 1231, 1122, 1042, 855; **^{1}H NMR** (300 MHz, $CDCl_3$) δ 4.99–5.20 (m, 1H, CHF), 3.72–4.09 (m, 2H, $CFCH_2N$), 3.74 (s, 3H, OCH_3), 2.57–2.70 (m, 2H, CH_2CO), 1.49 (s, 18H, 6 × CH_3); **^{13}C{^{1}H} NMR**

(75 MHz, $CDCl_3$) δ 170.1 (d, *J* = 5.7 Hz, CO_2CH_3), 152.5 (NCO_2), 88.7 (*J* = 173.9 Hz, CHF), 83.0 ($C(CH_3)_3$), 52.1 (OCH_3), 48.9 (d, *J* = 23.6 Hz, CH_2N), 38.0 (d, *J* = 23.3 Hz, CO<u>C</u>H_2CF), 28.1 (6 × CH_3); **^{19}F NMR** (280 MHz, $CDCl_3$) δ −186.2 (m, 1F); **^{19}F{^{1}H} NMR** (280 MHz, $CDCl_3$) δ −186.2 (s, 1F); **HRMS** (ESI, +ve) $C_{15}H_{26}FNO_6Na^+$ [M+Na$^+$] requires *m/z* 358.1636, found 358.1629.

Difluoropiperidinedione 25

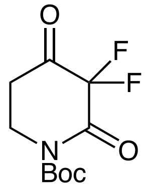

To a solution of piperidinedione **21a** (10 mg, 0.04 mmol) in acetonitrile (1 mL) at 0 °C was added NFSI (16.6 mg, 0.04 mmol) over 10 min. The reaction was stirred at 0 °C for 3 h then at room temperature for 16 h. The reaction mixture was diluted with dichloromethane and quenched with saturated aqueous $NaHCO_3$. The aqueous layer was extracted with dichloromethane (20 mL). The organic layers were combined, dried over $MgSO_4$ and concentrated onto silica. Flash chromatography provided the title compound as an off-white solid (2.8 mg, 52%); **^{1}H NMR** (300 MHz, $CDCl_3$) δ 4.24 (t, *J* = 6.2 Hz, 2H, C<u>H</u>$_2$–N), 2.91 (t, *J* = 6.2 Hz, 2H, C<u>H</u>$_2$–CO), 1.56 (s, 9H, *N*-Boc); **^{19}F NMR** (282 MHz, $CDCl_3$) δ −118.2 (s, 2F, CF_2); **^{19}F{^{1}H} NMR** (282 MHz, $CDCl_3$) δ −118.2 (s, 2F, CF_2); **HRMS** (ESI, +ve) $C_{24}H_{23}FN_2O_3^+$ requires *m/z* 250.0885, found 250.0696.

Aldol product 26

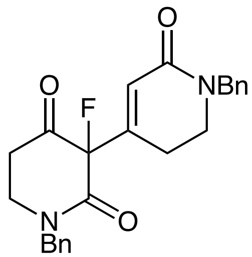

To a suspension of freshly ground sodium carbonate (25.2 mg, 0.23 mmol, 1.5 equiv) and NFSI (50 mg, 0.15 mmol, 1.0 equiv) in tetrahydrofuran (2.0 mL) at 0 °C was added (*R*)-1-(naphthalen-2-

yl)ethanamine trichloroacetic acid salt monohydrate (0.68 mmol, 0.12 equiv) as a solution in tetrahydrofuran (0.3 mL). The reaction was stirred at 0 °C for 10 min before piperidinedione **21b** (64 mg, 3.2 mmol) was added in three portions. The reaction was stirred at 0 °C for 24 h, before dichloromethane (20 mL) was added. The reaction was filtered through silica to remove insolubles, eluting with dichloromethane (25 mL). The filtrate was concentrated under reduced pressure and crude solid compound purified by flash chromatography (5→95% ethyl acetate / dichloromethane) to give the title compound as a white solid (53 mg, 42%); **m.p.** 158−162 °C; **IR** (neat) ν_{max} (cm^{-1}) 2917, 1630, 1741, 1654, 1488, 1048, 720; **^{1}H NMR** (300 MHz, $CDCl_3$) δ 7.42−7.25 (m, 10H, ArH), 6.0−5.95 (m, 1H, C<u>H</u>=C−CH_2), 4.95−4.56 (m, 4H, ArH−C$\underline{H}_2$), 3.65−3.42 (m, 2H, N−C$\underline{H}_2$−CHF), 3.36 (t, *J* = 6.9 Hz, 2H, CO−C$\underline{H}_2$), 2.84−2.57 (m, 2H, N−C$\underline{H}_2$), 2.56−2.23 (m, 2H, CO−C$\underline{H}_2$); **^{13}C{^{1}H} NMR** (75 MHz, $CDCl_3$) δ 198.8, 164.9, 164.6, 164.0, 144.2, 137.8, 136.5, 130.5, 130.2, 129.9, 129.8, 129.4, 129.2, 127.2, 127.1, 97.3 (d, *J* = 195.3 Hz, <u>C</u>−F), 52.7, 52.1, 45.6, 42.06, 37.0; **^{19}F NMR** (282 MHz, $CDCl_3$) δ −163.9 (m, 1F, C−F); **^{19}F{^{1}H} NMR** (282 MHz, $CDCl_3$) δ −163.9 (s, 1F, C−F); **HRMS** (ESI, +ve) $C_{24}H_{23}FN_2O_3Na^+$ requires *m*/*z* 429.1585, found 429.1576.

Epoxy alcohol 28b

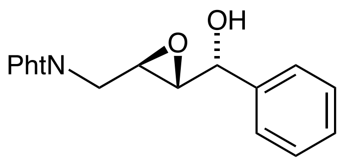

A stirred suspension of activated powdered 4 Å molecular sieves (0.1 g) in dichloromethane (1.2 mL) was cooled to –20 °C. A solution of D-(–)-diethyl tartrate (34 mg, 0.16 mmol) in dichloromethane (0.2 mL), followed by titanium(IV) isopropoxide (40 µL, 0.14 mmol), were added dropwise. After stirring for 20 min, precooled tert-butyl hydroperoxide (5.5 M in decane, 50 µL, 0.27 mmol) was added dropwise and the resulting mixture was stirred for a further 30 min. A precooled solution of **30** (40 mg, 0.14 mmol) in dichloromethane (0.5 mL) was then added dropwise over 20 min to the reaction mixture. The mixture was stirred for a further 1 h at –20 °C before placing in a freezer (–18 °C) for 3 days. A solution of citric acid monohydrate (29 mg, 0.14 mmol) in 10% acetone/diethyl ether (4 mL) was added dropwise to the cold reaction mixture which was then stirred for 30 min without cooling. The mixture was filtered through celite, and the filtrate concentrated in vacuo. Flash chromatography (19:1–93:7 dichloromethane/ethyl acetate) provided a colourless oil comprising a mixture of **28b** (27 mg, 62%,

95% *de*) and D-(–)-diethyl tartrate (9 mg). Data for **28b**: **^{1}H NMR** (200 MHz, $CDCl_3$) δ 7.82–7.70 (m, 4H, phthalimide), 7.35–7.22 (m, 5H, phenyl), 4.86 (m, 1H, CH–OH), 3.95 (dd, *J* = 14.4, 5.4 Hz, 1H, CHH), 3.79 (dd, *J* = 14.4, 5.0 Hz, 1H, CHH), 3.47 (m, 1H, CH–CH_2), 3.22 (dd, *J* = 3.4, 2.1 Hz, 1H, CH–CH–CH_2); **MS** (ESI, +ve) *m/z* 332 (MNa^+, 100%), 641 (M_2Na^+, 72%).

Trifluoroalkanes 29a/b (diastereoisomeric mixture) and 34

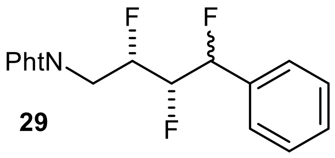

29

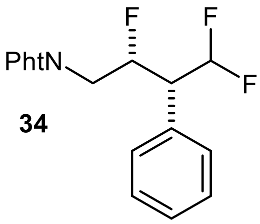

34

DeoxoFluor (0.1 mL, 0.5 mmol) was added to a plastic reaction vessel containing a mixture of **33**/**34** (7.5 mg, 0.023 mmol). The resulting mixture was immediately heated to 70 °C and stirred for 16 h, then cooled to room temperature. Examination of the mixture by ^{19}F{^{1}H} NMR indicated that complete consumption of the starting material had occurred and a 2.3 : 1 : 4.6 ratio of **29a** : **29b** : **34** had formed.

Data for **29a/29b**: **^{19}F{^{1}H} NMR** (282 MHz, $CDCl_3$) –185.9 (m, 0.7F), –187.0 (m, 0.7F), –187.8 (m, 0.7F), –205.2 (m, 0.3F), –207.1 (m, 0.3F), –211.6 (m, 0.3F).

Data for **34**: **^{19}F{^{1}H} NMR** (282 MHz, $CDCl_3$) –121.2 (m, 2F, CHF_2), –197.3 (s, 1F, CHF–CH_2).

Allylic alcohol 30

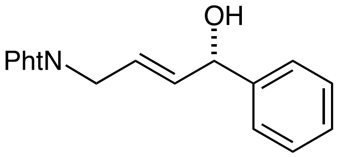

A solution of **27** (491 mg, 3.66 mmol) and *N*-allylphthalimide (3.43 g, 18.3 mmol) in dichloromethane (28 mL) was transferred via cannula to a flask containing Grubbs' 2nd generation catalyst (31 mg, 0.037 mmol). The resulting mixture was heated to reflux for 14 h, then concentrated in vacuo. Purification by flash chromatography (24:1–17:3 dichloromethane/ethyl acetate) provided **30** as a white

solid (554 mg, 52%); **m.p.** 78–80 °C; $[\alpha]_D$ –8 (*c* 0.20, $CHCl_3$); **IR** (neat) ν_{max} (cm^{-1}) 3386, 1770, 1695, 1612, 1424, 1391, 1362, 1324, 1108; **^{1}H NMR** (300 MHz, $CDCl_3$) δ 7.80–7.67 (m, 4H, phthalimide), 7.36–7.22 (m, 5H, phenyl), 5.95 (dd, *J* = 15.4, 5.5 Hz, 1H, OCH–CH), 5.84 (dt, *J* = 15.4, 5.5 Hz, 1H, CH–CH_2), 5.19 (d, *J* = 5.5 Hz, 1H, OCH), 4.28 (d, *J* = 5.5 Hz, 2H, CH_2), 3.13 (br s, 1H, OH); **^{13}C{^{1}H} NMR** (75 MHz, $CDCl_3$) δ 168.0, 142.6, 136.1, 134.0, 132.0, 128.5, 127.6, 126.5, 124.3, 123.3, 74.0, 39.1; **MS** (ESI, +ve) *m/z* 316 (MNa^+, 100%), 609 (M_2Na^+, 42%); **HRMS** (ESI, +ve) $C_{18}H_{15}NO_3Na^+$ requires *m/z* 316.0944, found 316.0947; **elemental analysis** $C_{18}H_{15}NO_3$ requires C 73.7, H 5.2, N 4.8, found C 73.5, H 5.2, N 4.7%.

Fluoroepoxide 31 (diastereoisomeric mixture)

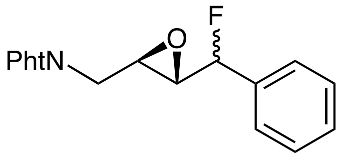

DeoxoFluor (9.8 μL, 0.053 mmol) was added dropwise to a solution of **28b** (11 mg, 0.036 mmol) in dichloromethane (0.5 mL) at –78 °C. The reaction mixture was stirred at –78 °C for 2.5 h, then stored in a freezer (–18 °C) for 16 h. The reaction mixture was concentrated in vacuo and purified by flash chromatography (1:0–1:1 dichloromethane/ethyl acetate) to provide the title compound as an inseparable 2:1 mixture of diastereoisomers (colourless oil, 9 mg, 81%).

Data for **31a**: **^{1}H NMR** (300 MHz, $CDCl_3$) δ 7.86–7.71 (m, 4H, phthalimide), 7.34–7.28 (m, 5H, phenyl), 5.39 (dd, *J* = 47.5, 3.5 Hz, 1H, CHF), 4.01–3.77 (m, 2H, CH_2), 3.36–3.26 (m, 2H, $O(CH)_2$); **^{19}F NMR** (282 MHz, $CDCl_3$) δ –185.1 (dd, *J* = 47.5, 11.2 Hz, 1F, CHF); **MS** (ESI, +ve) *m/z* 334 (MNa^+, 100%).

Data for **31b**: **^{1}H NMR** (300 MHz, $CDCl_3$) δ 7.86–7.71 (m, 4H, phthalimide), 7.34–7.28 (m, 5H, phenyl), 5.23 (dd, *J* = 47.5, 5.2 Hz, 1H, CHF), 4.01–3.77 (m, 2H, CH_2), 3.36–3.26 (m, 2H, $O(CH)_2$); **^{19}F NMR** (282 MHz, $CDCl_3$) δ –183.2 (dd, *J* = 47.5, 12.8 Hz, 1F, CHF); **MS** (ESI, +ve) m/z 334 (MNa^+, 100%).

Difluoroalcohols 32a/b and 33a/b (diastereiosomeric mixtures)

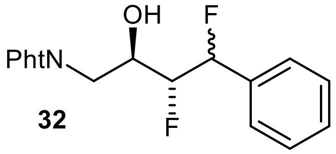

32

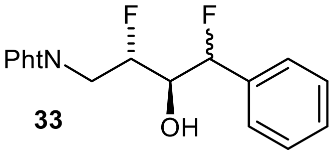

33

A mixture of compound **31** (2:1 mixture of diastereoisomers, 30 mg, 0.096 mmol) and triethylamine trihydrofluoride (0.5 mL) was stirred at 150 °C in a sealed tube for 18 h. The reaction mixture was then cooled to 0 °C, diluted with dichloromethane (20 mL) and washed with saturated aqueous $NaHCO_3$ (2 × 20 mL) and saturated aqueous NH_4Cl (20 mL). The organic layer was dried ($MgSO_4$) and concentrated in vacuo. Purification by flash chromatography (4:1 3:1 petroleum ether/ethyl acetate) provided an inseparable: 1.5 : 1 : 7.5 : 5 mixture of **32a**/**b** and **33a**/**b** as a colourless oil (9 mg, 28%).

Data for **32a**/**b**: **^{19}F NMR** (282 MHz, $CDCl_3$) δ –189.1 (ddd, *J* = 44.4, 13.8, 13.4 Hz, 0.4F, phenyl–CHF), –197.4 (ddd, *J* = 44.8, 24.1, 9.5 Hz, 0.6F, phenyl–CHF), –205.0 (m, 0.4F, OCH–CHF), –208.3 (m, 0.6F, OCH–CHF); **^{19}F{^{1}H} NMR** (282 MHz, $CDCl_3$) δ –189.1 (d, *J* = 13.8 Hz, 0.4F, phenyl–CHF), –197.4 (d, *J* = 9.5 Hz, 0.6F, phenyl–CHF), –205.0 (d, *J* = 13.9 Hz, 0.4F, OCH–CHF), –208.3 (d, *J* = 9.5 Hz, 0.6F, OCH–CHF).

Data for **33a**/**b**: **^{1}H NMR** (300 MHz, $CDCl_3$) δ 7.90–7.73 (m, 4H, phthalimide), 7.42–7.34 (m, 5H, phenyl), 5.70 (dm, *J* = 45.7 Hz, 0.4H, phenyl–CHF), 5.63 (dm, *J* = 44.7 Hz, 0.6H, phenyl–CHF), 4.94 (dm, *J* = 46.6 Hz, 0.4H, CHF–CH_2), 4.57 (dm, *J* = 46.6 Hz, 0.6H, CHF–CH_2), 4.24–4.08 (m, 3H, OCH and CH_2); **^{19}F NMR** (282 MHz, $CDCl_3$) δ –188.2 (dd, *J* = 44.8, 12.0 Hz, 0.6F, phenyl–CHF), –196.0 (m, 0.4F, CHF–CH_2), –196.4 (m, 0.6F, CHF–CH_2), –200.6 (ddd, *J* = 45.7, 24.1, 2.6 Hz, 0.4F, phenyl–CHF); **^{19}F{^{1}H} NMR** (282 MHz, $CDCl_3$) δ –188.2 (s, 0.6F, phenyl–CHF), –196.0 (d, *J* = 2.6 Hz, 0.4F, CHF–CH_2), –196.4 (s, 0.6F, CHF–CH_2), –200.6 (d, *J* = 2.6 Hz, 0.4F, phenyl–CHF); **MS** (ESI, +ve) *m*/*z* 354 (MNa^+, 4%), 685 (M_2Na^+, 10%).

Allylic alcohol 36[1]

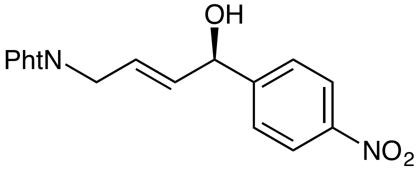

A solution of Grubbs 2nd generation catalyst (376 mg, 0.44 mmol) in anhydrous dichloromethane (320 mL) was added *via* cannula to a stirred mixture of **35** (5.3 g, 29.5 mmol) and *N*-allylphthalimide (27.6 g, 148 mmol) in anhydrous dichloromethane (200 mL). The resulting mixture was then heated to reflux for 16 h. Dichloromethane (200 mL) was added and the mixture was filtered and concentrated *in vacuo*. The crude solid product was washed with ethyl acetate/hexane (1:3, 200 mL) and then further purified by flash chromatography (9:1→7:3 dichloromethane/ethyl acetate) to give allylic alcohol **36**[1] as an off white powder (6.5 g, 65%); **[α]D** −10 (*c* 0.2, $CHCl_3$); **IR** (neat) ν_{max} (cm^{-1}) 3797, 3432, 1766, 1696, 1517, 1425, 1389, 1342, 1059; **^{1}H NMR** (300 MHz, $CDCl_3$) δ 8.19 (d, *J* = 8.6 Hz, 2H, *p*-nitrophenyl), 7.88−7.69 (m, 4H, phthalimide ArH), 7.52 (d, *J* = 8.6 Hz, 2H, *p*-nitrophenyl), 5.95−5.81 (m, 2H, C<u>H</u>=C<u>H</u>−CH_2), 5.31 (t, *J* = 4.0 Hz, 1H, OCH), 4.32 (d, *J* = 3.6 Hz, 2H, CH_2), 2.24 (d, *J* = 3.6 Hz, 1H, OH); **^{13}C {^{1}H} NMR** (75 MHz, $CDCl_3$) δ 167.8, 149.2, 147.4, 134.5, 134.1, 131.9, 127.0, 126.3, 123.8, 123.4, 73.3, 38.8; **MS** (APCI, +ve) *m/z* 321 ($[M–OH]^+$, 26%); **elemental analysis** $C_{18}H_{14}N_2O_5$ requires C 63.9, H 4.2, N 8.3, found C 64.0, H 4.2, N 8.4%.

Epoxy alcohol 37[1]

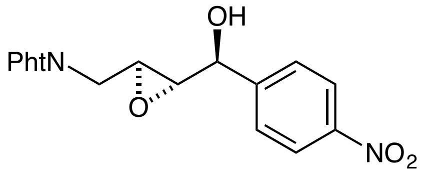

A stirred suspension of activated powdered 4Å molecular sieves (12.0 g) in anhydrous dichloromethane (120 mL) was cooled to −20 °C. A solution of (+)-diethyl-L-tartrate (4.53 g, 21.9 mmol) in anhydrous dichloromethane (30 mL), followed by titanium (IV) isopropoxide (5.3 mL, 18.3 mmol) were added dropwise. After stirring for 20 min, *tert*-butyl hydroperoxide (5.5 M in decane, 6.0 mL, 37 mmol) was added dropwise and the mixture was left stirring for another 30 minutes. A solution of allylic alcohol **36** (6.2 g, 18 mmol) in anhydrous dichloromethane (900 mL) was added and the mixture stirred for another 30 min at −20 °C before being placed in a freezer at −20 °C for 3 days, left undisturbed. A solution of citric acid monohydrate (3.84 g, 18.3 mmol) in 10% acetone/diethyl ether (200 mL) was added dropwise to the reaction mixture at −20 °C. The mixture was then filtered through celite and the filtrate concentrated *in vacuo*. The solid residue was washed with toluene (3 × 80 mL) and dichloromethane (30 mL) to afford epoxy alcohol **37**[1] as a white solid (4.5 g, 69%); **[α]D** +52.2 (*c* 0.15, $CHCl_3$); **IR** (neat) ν_{max} (cm^{-1}) 3461, 1767, 1698, 1519, 1386, 1347, 1005; **^{1}H NMR** (300 MHz,

$CDCl_3$) δ 8.08 (d, *J* = 8.8 Hz, 2H, *p*-nitrophenyl), 7.80–7.68 (m, 4H, phthalimide), 7.50 (d, *J* = 8.8 Hz, 2H, *p*-nitrophenyl), 4.98–4.94 (m, 1H, C<u>H</u>–OH), 4.01 (dd, *J* = 14.3, 4.8 Hz, 1H, C<u>H</u>H), 3.66 (dd, *J* = 14.3, 5.9 Hz, 1H, CH<u>H</u>), 3.41–3.35 (m, 1H, C<u>H</u>–CH_2), 3.23 (dd, *J* = 3.4, 2.2 Hz, 1H, C<u>H</u>–CH–CH_2), 2.50 (d, *J* = 2.1 Hz, 1H, OH); **$^{13}C\{^1H\}$ NMR** (75 MHz, $CDCl_3$) δ 167.7, 147.6, 146.4, 134.3, 131.6, 126.7, 123.6, 123.4, 69.6, 59.8, 51.4, 38.6; MS (ESI, +ve) m/z 377 (MNa+, 35%), 731 (M_2Na^+, 100%); **HRMS** (ESI, +ve) $C_{18}H_{14}N_2O_6Na^+$ requires *m*/*z* 377.0744, found 377.0745; **elemental analysis** $C_{18}H_{14}N_2O_6$ requires C 61.0, H 4.0, N 7.9, found C 60.1, H 3.9, N 7.9%.

Fluoroexpoxide 38a[1]

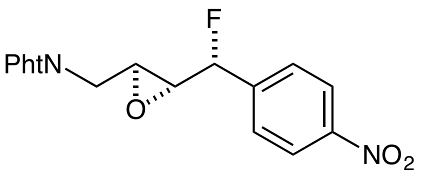

N-(Trimethylsilyl)morpholine (1.0 mL, 5.6 mmol) was added dropwise to a solution of DeoxoFluor (1.0 mL, 5.6 mmol) in anhydrous dichloromethane (10 mL) at −78 °C. The solution was stirred for 2.5 h at room temperature. The solution was then cooled to −78 °C and a solution of **37** (500 mg, 1.41 mmol) in anhydrous dichloromethane (50 mL) was added *via* cannula. The mixture was then heated to reflux for 19 h and then concentrated onto silica. Purification was performed using flash chromatography (1:0→17:3 dichloromethane/ethyl acetate) to give the title compound[1] as a yellow solid (465 mg, 94%); **m.p.** 163−166 °C; **$[\alpha]_D$** +28.5 (*c* 0.03, $CHCl_3$); **IR** (neat) ν_{max} (cm^{-1}) 3747, 3652, 3467, 3275, 1712, 1519, 1392, 1345, 1004; **^1H NMR** (300 MHz, $CDCl_3$) δ 8.15 (d, *J* = 8.5 Hz, 2H, *p*-nitrophenyl), 7.83−7.70 (m, 4H, phthalimide ArH), 7.50 (d, *J* = 8.5 Hz, 2H, *p*-nitrophenyl), 5.40 (dd, *J* = 46.9, 5.0 Hz, 1H, CHF), 4.05 (dd, *J* = 14.3, 4.4 Hz, 1H, C<u>H</u>H), 3.75 (dd, *J* = 14.3, 5.6 Hz, 1H, CH<u>H</u>), 3.34−3.28 (m, 2H, $O(CH)_2$); **^{13}C {^1H} NMR** (75 MHz, $CDCl_3$) δ 167.7, 148.2, 142.5, (d, *J* = 21.6 Hz, <u>C</u>−CHF), 134.4, 131.6, 126.8 (d, *J* = 6.9 Hz, <u>C</u>H−C−CHF), 126.7, 123.8, 123.5, 91.0 (d, *J* = 180.5 Hz, CHF), 58.2, (d, *J* = 26.7 Hz, <u>C</u>H−CHF), 52.3 (d, *J* = 7.2 Hz, <u>C</u>H−CH−CHF), 38.3; **^{19}F NMR** (282 MHz, $CDCl_3$) δ −187.9 (dd, *J* = 47.0, 13.5 Hz, 1F, CHF); **$^{19}F\{^1H\}$ NMR** (282 MHz, $CDCl_3$) δ −187.9 (s, 1F, CHF); **MS** (ESI, +ve) *m*/*z* 379 (MNa^+, 100%), 735 (M_2Na^+, 68%); **HRMS** (ESI, +ve) $C_{18}H_{13}FN_2O_5Na^+$ requires *m*/*z* 379.0701, found 379.0708.

Fluoroepoxide 38b[1]

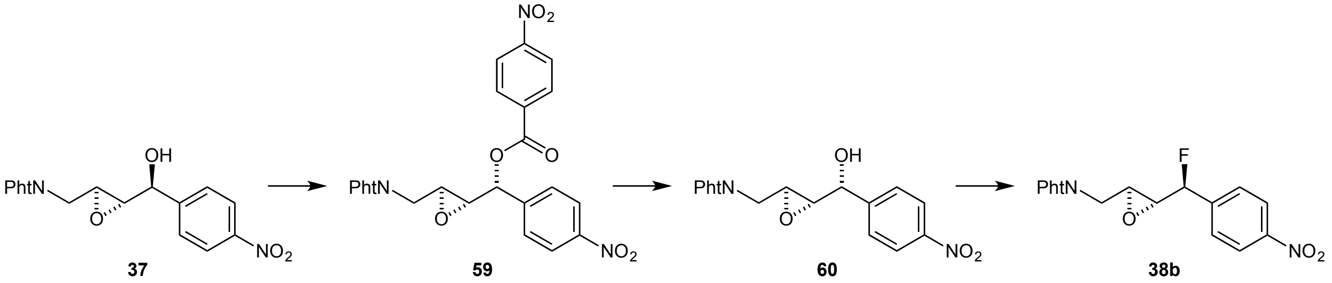

A solution of triphenylphosphine (2.9 g, 11.2 mmol) in anhydrous tetrahydrofuran (25 mL) was cooled to 0 °C. Diisopropyl azodicarboxylate (2.28 mL, 11.2 mmol) was then added dropwise and the mixture was stirred for 20 min. A solution of *p*-nitrobenzoic acid (1. 8 g, 11.2 mmol) in anhydrous tetrahydrofuran (22 mL) was then added dropwise and stirring was continued for a further 20 min. A solution of **37** (2.0 g, 5.2 mmol) in dry tetrahydrofuran (25 mL) was added *via* cannula. The mixture was slowly warmed to rt and stirred overnight. The reaction mixture was then concentrated onto silica and purified by flash chromatography (dichloromethane). The resulting partially-purified solid product was washed with methanol (10 mL) to provide ester **59**[1] as a white solid (2.35 g, 85%); **m.p.** 123–125 °C; **IR** (neat) ν_{max} (cm^{-1}) 2854, 1771, 1702, 1605, 1520, 1389, 1346, 1268, 1159, 943, 714; **$[\alpha]_D$** +69.2 (*c* 0.13, $CHCl_3$); **^{1}H NMR** (300 MHz, $CDCl_3$) δ 8.32–8.25 (m, 4H, *p*-nitrobenzoate), 8.12 (d, *J* = 8.8 Hz, 2H, *p*-nitrophenyl), 7.81–7.69 (m, 4H, phthalimide), 7.58 (d, *J* = 8.8 Hz, 2H, *p*-nitrophenyl), 5.90 (d, *J* = 5.7 Hz, 1H, CH–OCO), 4.05 (dd, *J* = 14.3, 4.6 Hz, 1H, C<u>H</u>H), 3.74 (dd, *J* = 14.3, 5.9 Hz, 1H, CH<u>H</u>), 3.48 (dd, *J* = 5.7, 2.0 Hz, 1H, C<u>H</u>–CH–CH_2), 3.33 (m, 1H, C<u>H</u>–CH_2); **$^{13}C\{^1H\}$ NMR** (75 MHz, $CDCl_3$) δ 167.7, 163.3, 150.9, 148.1, 142.6, 134.4, 134.3, 131.5, 131.0, 127.7, 124.0, 123.7, 123.5, 75.3, 58.1, 53.0, 38.4; **MS** (ESI, +ve) *m/z* 526 (MNa^+, 43%); **HRMS** (ESI, +ve) $C_{25}H_{17}N_3O_9Na^+$ requires *m/z* 526.0857, found 526.0854.

A flask containing ester **59** (2.1 g, 4.17 mmol) was cooled to 0 °C. Methanol (30 mL) followed by potassium carbonate (0.3 g, 2.0 mmol) were slowly added. The resulting mixture was stirred at 0 °C for 1 h, then diluted with dichloromethane (40 mL) and washed with water (25 mL). The organic layer was dried over Na_2SO_4, concentrated, and the crude product was purified by flash chromatography (dichloromethane→dichloromethane/ethyl acetate 1:0→4:1) to give compound **60**[1] as a white solid (1.25 g, 83%); **m.p.** 151–153 °C; **$[\alpha]_D$** +2.0 (*c* 0.04, $CHCl_3$); **^{1}H NMR** (300 MHz, $CDCl_3$) δ 8.13 (d, *J* = 8.8 Hz, 2H, *p*-nitrophenyl), 7.84–7.71 (m, 4H, phthalimide), 7.53 (d, *J* = 8.8 Hz, 2H, *p*-nitrophenyl), 4.69 (d, *J* = 4.9 Hz, 1H, C<u>H</u>–OH), 4.03 (dd, *J* = 14.4, 4.8 Hz, 1H, C<u>H</u>H), 3.77 (dd, *J* = 14.4, 5.5 Hz,

1H, CH$\underline{H}$), 3.37 (ddd, J = 6.9, 5.2, 2.1 Hz, 1H, C$\underline{H}$–CH_2), 3.20 (dd, J = 4.9, 2.1 Hz, 1H, C$\underline{H}$–CH–CH-$_2$); **^{13}C{^{1}H} NMR** (75 MHz, $CDCl_3$) δ 167.8, 147.6, 146.8, 134.3, 131.6, 127.0, 124.0, 123.8, 123.5, 72.2, 60.4, 53.3, 38.5; **MS** (APCI, +ve) *m/z* 355 (MH^+, 87%); **HRMS** (ESI, +ve) $C_{18}H_{15}N_2O_6^+$ requires *m/z* 355.0925, found 355.0926.

N-(Trimethylsilyl)morpholine (3.0 mL, 17.2 mmol) was added dropwise to a solution of DeoxoFluor (3.1 mL, 16.9 mmol) in dichloromethane (30.0 mL) at −78 °C. A solution of **60** (1.0 g, 2.8 mmol) in anhydrous dichloromethane (3.0 mL) was then slowly added *via* cannula. The resulting mixture was heated to reflux for 18 h and then concentrated onto silica. Purification by flash chromatography (dichloromethane) gave fluoroepoxide **38b**[1] as an off-white solid (0.83 g, 83% yield) **m.p.** 126–129 °C; **[α]$_D$** −53.0 (*c* 0.06, $CHCl_3$); **^{1}H NMR** (300 MHz, $CDCl_3$) δ 8.20 (d, J = 8.9 Hz, 2H, *p*-nitrophenyl), 7.87–7.71 (m, 4H, phthalimide), 7.53 (d, J = 8.9 Hz, 2H, *p*-nitrophenyl), 5.45 (dd, J = 46.7, 4.0 Hz, 1H, CHF), 4.01 (dd, J = 14.4, 4.8 Hz, 1H, C$\underline{H}$H), 3.82 (dd, J = 14.4, 5.0 Hz, 1H, CH$\underline{H}$), 3.32–3.24 (m, 2H, 2 × OCH); **^{13}C{^{1}H} NMR** (75 MHz, $CDCl_3$) δ 167.9 (phthalimide CO), 148.5 (ArC–NO_2), 142.7 (d, J = 20.6 Hz, Ar$\underline{C}$–CHF), 134.5, 131.9, 127.0 (d, J = 7.3 Hz), 123.9, 123.7, 90.7 (d, J = 177.9 Hz, CHF), 57.6 (d, J = 30.7 Hz, O$\underline{C}$H–CHF), 53.2 (d, J = 4.6 Hz, O$\underline{C}$H–CH–CHF), 38.6 (CH_2); **^{19}F NMR** (282 MHz, $CDCl_3$) δ −188.5 (dd, J = 46.7, 9.7 Hz, 1F, CHF); **^{19}F {^{1}H} NMR** (282 MHz, $CDCl_3$) δ −188.5 (s, 1F, CHF); **HRMS** (ESI, +ve) $C_{18}H_{13}FN_2O_5Na^+$ [MNa^+] requires *m/z* 379.0701, found 379.0702.

Difluoroalcohols 39a and regioisomer[1]

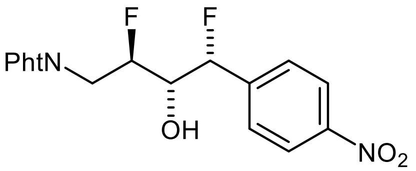

39a

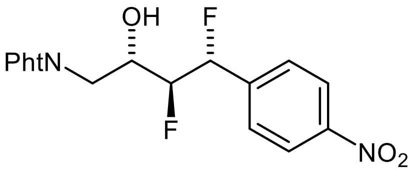

regioisomer of **39a**

Triethylamine trihydrofluoride (1.2 mL) was added to a microwave tube containing compound **39a** (100 mg, 0.28 mmol). The mixture was subjected to microwave irradiation at 150 °C for 15 min. The reaction mixture was then diluted with dichloromethane and quenched with saturated aqueous $NaHCO_3$. The mixture was extracted with ethyl acetate (25 mL) and dichloromethane (30 mL). The organic layers were combined, dried over $MgSO_4$ and concentrated onto silica. The crude product was

purified by flash chromatography (dichloromethane→dichloromethane/ethyl acetate 10:1) to provide a mixture of compounds **39a**[1] and its regioisomer as a pale brown solid (44 mg, 46% combined yield).

Data for **39a**:[1] **m.p.** 226–228 °C; **^{1}H NMR** (300 MHz, $CDCl_3$) δ 8.25 (d, *J* = 8.7 Hz, 2H, *p*-nitrophenyl), 7.92–7.73 (m, 4H, phthalimide), 7.56 (d, *J* = 8.7 Hz, 2H, *p*-nitrophenyl), 5.79 (dm, *J* = 45.4 Hz, 1H, *p*-nitrophenyl–CHF), 4.98 (dddd, *J* = 45.9, 8.9, 3.7, 3.5 Hz, 1H, C<u>H</u>F–CH_2), 4.33 (ddd, *J* = 17.2, 15.2, 3.5 Hz, 1H, C<u>H</u>H), 4.11 (ddd, *J* = 30.2, 15.2, 3.7 Hz, 1H, CH<u>H</u>), 3.80–3.65 (m, 1H, OCH), 3.61 (d, *J* = 6.8 Hz 1H, OH); **^{19}F NMR** (282 MHz, $CDCl_3$) δ –195.8 (m, 1F, CHF–CH_2), –203.8 (ddd, *J* = 45.3, 24.9, 2.6 Hz, 1F, *p*-nitrophenyl–CHF); **^{19}F {^{1}H} NMR** (282 MHz, $CDCl_3$) δ –195.8 (d, *J* = 2.5 Hz, 1F, CHF–CH_2), –203.8 (d, *J* = 2.6 Hz, 1F, *p*-nitrophenyl–CHF).

Data for the regioisomer of **39a**:[1] **^{19}F NMR** (282 MHz, $CDCl_3$) δ –191.7 (ddd, *J* = 44.2, 12.9, 12.9 Hz, 1F, *p*-nitrophenyl–CHF), –205.3 (m, 1F, OCH–CHF); **^{19}F {^{1}H} NMR** (282 MHz, $CDCl_3$) δ –191.7 (d, *J* = 12.9 Hz, 1F, *p*-nitrophenyl–CHF), –205.3 (d, *J* = 12.9 Hz, 1F, OCH–CHF).

Difluoroalcohol 39b and regioisomer[1]

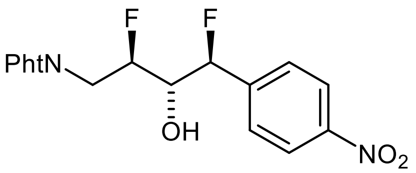

39b

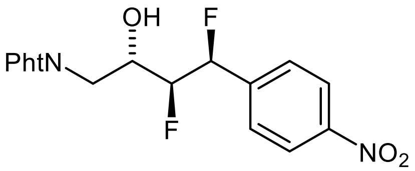

regioisomer of **39b**

Triethylamine trihydrofluoride (1.0 mL) was added to a microwave tube containing compound **38b** (50 mg, 0.14 mmol). The mixture was subjected to microwave irradiation at 150 °C for 10 min. The reaction mixture was then diluted with ethyl acetate (15 mL) and quenched with saturated aqueous $NaHCO_3$ (10 mL). The phases were separated, and the aqueous portion was extracted with ethyl acetate (3 × 25 mL). The combined organic layers were dried with Na_2SO_4 and concentrated onto silica. The crude product was subjected to flash chromatography (dichloromethane→dichloromethane/ethyl acetate 10:1) to provide an inseparable mixture of compounds **39b**[1] and its regioisomer as a brown solid (total 23.5 mg, 45%).

Data for **39b**:[1] **^{1}H NMR** (300 MHz, $CDCl_3$) δ 8.25 (d, *J* = 8.6 Hz, 2H, *p*-nitrophenyl), 7.91–7.76 (m, 4H, phthalimide), 7.59 (d, *J* = 8.6 Hz, 2H, *p*-nitrophenyl), 5.73 (m, 1H, *p*-nitrophenyl–C<u>H</u>F), 4.47 (m,

1H, CHF−CH_2), 4.24–3.97 (m, 3H, OCH and CH_2); **^{19}F NMR** (282 MHz, $CDCl_3$) δ −190.7 (dd, *J* = 44.7, 12.7 Hz, 1F, CHF), −195.6 (m, 1F, CHF–CH_2); **^{19}F {^{1}H} NMR** (282 MHz, $CDCl_3$) δ −190.7 (s, 1F, *p*-nitrophenyl–CHF), −195.6 (s, 1F, CHF−CH_2).

Data for the regioisomer of **39b**:[1] **^{19}F NMR** (282 MHz, $CDCl_3$) δ –202.5 (ddd, *J* = 44.0, 26.3, 8.7 Hz, 1F, *p*-nitrophenyl–CHF), –208.6 (m, 1F, CHF–CH_2); **^{19}F {^{1}H} NMR** (282 MHz, $CDCl_3$) δ –202.5 (d, *J* = 8.7 Hz, 1F, *p*-nitrophenyl–CHF), –208.6 (d, *J* = 8.7 Hz, 1F, CHF−CH_2).

Trifluoroalkane 40a[1]

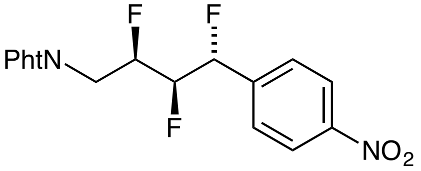

Deoxo-Fluor^{TM} (0.1 mL, 052 mmol) was added to a plastic reaction vessel containing **39a** (regioisomeric mixture, 100 mg, 0.26 mmol). The resulting mixture was immediately heated to 70 °C and stirred for 19 h. The reaction mixture was cooled to room temperature, diluted with dichloromethane (100 mL) and washed with saturated aqueous $NaHCO_3$ (120 mL). The organic layer was dried ($MgSO_4$) and concentrated onto silica. Purification by flash chromatography (4:1→18:7 hexanes/ethyl acetate) provided **40a** as an off-white solid (48 mg, 48%); **m.p.** 193–196 °C; **[α]$_D$** +47.6 (*c* 0.021, $CHCl_3$); **IR** (neat) ν_{max} (cm^{-1}) 2342, 1771, 1714, 1527, 1384, 1344, 1110, 1021; **^{1}H NMR** (300 MHz, $CDCl_3$) δ 8.29 (d, *J* = 8.5 Hz, 2H, *p*-nitrophenyl), 7.91–7.75 (m, 4H, phthalimide), 7.61 (d, *J* = 8.5 Hz, 2H, *p*-nitrophenyl), 5.77 (ddd, *J* = 44.9, 8.7, 4.7 Hz, 1H, CHF–CHF–CHF–CH_2), 5.21 (dddddd, *J* = 46.7, 27.6, 8.3, 3.9, 2.7, 1.0 Hz, 1H, CHF–CH_2), 4.65 (ddddd, *J* = 45.0, 25.7, 8.7, 4.3, 1.0 Hz, 1H, CHF–CHF–CH_2), 4.34 (ddd, *J* = 14.6, 14.3, 8.3 Hz, 1H, CHH), 3.94 (ddd, *J* = 27.7, 14.6, 3.9 Hz, 1H, CHH); **^{13}C {^{1}H} NMR** (75 MHz, $CDCl_3$) δ 168.1, 148.9, 142.5 (d, *J* = 19.5 Hz, C–CHF), 134.7, 132.1, 127.7 (d, *J* = 7.5 Hz, CH–C–CHF), 124.1, 123.9, 92.0–85.6 (m, 3 × CHF), 38.2 (dd, *J* = 25.5, 6.5 Hz, CH_2); **^{19}F NMR** (282 MHz, $CDCl_3$) δ –191.9 (ddddd, *J* = 44.9, 19.0, 4.3, 3.5, 2.7 Hz, 1F, CHF–CHF–CHF–CH_2), –207.4 (dddddd, *J* = 46.7, 27.7, 25.7, 14.3, 9.5, 3.5 Hz, 1F, CHF–CH_2), –211.8 (ddddd, 45.0, 27.6, 19.0, 9.5, 4.7 Hz, 1F, CHF–CHF–CH_2); **^{19}F{^{1}H} NMR** (282 MHz, $CDCl_3$) δ –191.9 (dd, *J* = 19.0, 3.5 Hz, 1F, CHF–CHF–CHF–CH_2), –207.4 (dd, *J* = 9.5, 3.5 Hz, 1F, CHF–CH_2),

–211.8 (dd, *J* = 19.0, 9.5 Hz, 1F, CHF–CHF–CH_2); MS (APCI, +ve) m/z 379 (MH+, 55%); HRMS (ESI, +ve) C18H13F3N2O4Na+ requires m/z 401.0720, found 401.0714.

Trifluoroalkane 40b[1]

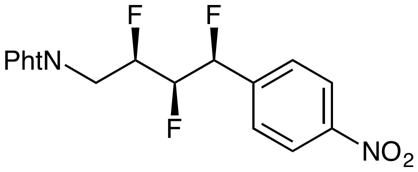

DeoxoFluor (2.10 mL) was added to a plastic reaction vessel containing **39b** (mixture of regioisomers, 64 mg, 0.170 mmol). The resulting mixture was immediately heated to 70 °C and stirred under nitrogen atmosphere overnight. The reaction mixture was cooled to room temperature, diluted with DCM, and quenched with saturated aqueous $NaHCO_3$. The mixture was extracted and the aqueous layer was washed with DCM (3 ×). The combined organic layers were then dried over $MgSO_4$ and concentrated onto silica. Purification by flash column chromatography (eluent: hexane/EtOAc 9:1→2:1 then DCM/hexane 15:1) provided the title compound as a white solid (18.0 mg, 28%); **m.p.** 170–172 °C; $[\alpha]_D$ –14 (*c* 0.07, $CHCl_3$); **IR** (DCM) ν_{max} (cm^{-1}) 2359, 1769, 1714, 1520, 1469, 1417, 1392, 1353, 1315, 1113; **^{1}H NMR** (600 MHz, $CDCl_3$) δ 8.28 (d, *J* = 8.5 Hz, 2H, *p*-nitrophenyl), 7.87–7.74 (m, 4H, phthalimide), 7.62 (d, *J* = 8.6 Hz, 2H, *p*-nitrophenyl), 5.99 (ddd, *J* = 6.1, 15.7, 47.0 Hz, 1H, *p*-nitrophenyl CHF), 4.80 (ddddd, *J* = 3.1, 6.1, 17.1, 23.4, 47.3 Hz, 1H, CHF–CHF–CHF), 4.64 (ddddd, *J* = 3.1, 3.3, 7.1, 22.5, 47.5 Hz, 1H, CHF–CH_2), 4.29 (ddd, *J* = 7.1, 15.0, 17.5 Hz, 1H, CHF–CH_2), 3.97 (ddd, *J* = 3.3, 15.0, 27.7 Hz, 1H, CHF–CH_2); **^{13}C{^{1}H} NMR** (75 MHz, HSQC, HMBC, $CDCl_3$) δ 168.0 (phthalimide CO), 148.8 (ArC–NO_2), 140.9 (dd, *J* = 6.3, 20.2 Hz, ArC–CHF), 134.6, 131.8, 127.7 (d, *J* = 7.0 Hz), 124.3, 123.8, 93.6 86.4 (m, 3 × CHF), 38.0 (dd, *J* = 8.6, 23.7 Hz, NCH_2CHF); **^{19}F{^{1}H} NMR** (282 MHz, $CDCl_3$) δ –192.3 (d, *J* = 14.4 Hz, 1F, *p*-nitrophenyl CHF), 203.2 (d, *J* = 11.3 Hz, 1F, CHF CH2), 206.8 (dd, J = 11.3, 14.4 Hz, 1F, CHF CHF CHF); **^{19}F NMR** (282 MHz, $CDCl_3$) δ –192.3 (ddd, *J* = 14.4, 17.1, 47.0 Hz, 1F, *p*-nitrophenyl CHF), –203.2 (ddddd, *J* = 11.3, 17.5, 23.4, 27.7, 47.5 Hz, 1F, CHF–CH_2), –206.8 (ddddd, *J* = 11.3, 14.4, 15.7, 22.5, 47.3, Hz, 1F, CHF–CHF–CHF); **HRMS** (ESI, +ve) $C_{18}H_{14}F_3N_2O_4^+$ [MH^+] requires *m/z* 379.0861, found 379.0900.

Benzylic fluoride 41

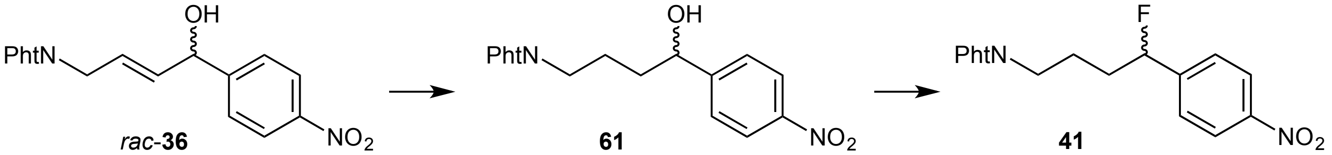

Pd/C (30 mg) was added to a solution of *rac*-**36** (300 mg, 0.88 mmol) in ethyl acetate (15 mL). The mixture was stirred under H_2 atmosphere for 5 h. The reaction mixture was filtered to remove the palladium residue which was washed with ethyl acetate (25 mL). Aqueous HCl (2M) was added, and the aqueous layer was extracted with ethyl acetate (2 × 20 mL). The combined organic layers were washed with brine (30 mL) and water (50 mL), dried ($MgSO_4$) and concentrated under reduced pressure. The crude residue was purified by flash silica gel column chromatography (25% ethyl acetate in hexanes) to give **61** as an off-white solid (180 mg, 60%); **m.p.** 260−265 °C; **IR** (neat) ν_{max} (cm^{-1}) 3461, 3042, 2923, 2864, 1769, 1720, 1517, 1397, 1345, 1070, 720; **^{1}H NMR** (300 MHz, $CDCl_3$) δ 8.21 (d, *J* = 8.8 Hz, 2H, *p*-nitrophenyl), 7.87−7.72 (m, 4H, phthalimide), 7.54 (d, *J* = 8.8 Hz, 2H, *p*-nitrophenyl), 4.93 (t, *J* = 4.6 Hz, 1H, OCH), 3.80−3.77 (m, 2H, NCH_2), 1.80−1.77 (m, 4H, $C\underline{H_2}$–$C\underline{H_2}$); **^{13}C{^{1}H} NMR** (75 MHz, $CDCl_3$) δ 130.1, 128.0, 122.6, 119.8, 119.3, 69.5, 32.0, 31.5, 20.8; **HRMS** (ESI, +ve) $C_{18}H_{16}N_2O_5Na^+$ [MNa^+] requires *m/z* 363.0951, found 363.0954.

DeoxoFluor^{TM} (0.1 mL, 0.52 mmol) was added to a solution of **61** (150 mg, 0.44 mmol) in dichloromethane (10 mL) and the solution was stirred at rt for 1 h. The reaction mixture was diluted with dichloromethane (10 mL) and washed with saturated aqueous $NaHCO_3$ (15 mL). The organic layer was dried ($MgSO_4$) and concentrated onto silica. Purification by flash chromatography (1:2→3:5 hexanes/ethyl acetate) provided **41** as a yellow solid (145 mg, 96%); **m.p.** 188–190 °C; **IR** (neat) ν_{max} (cm^{-1}) 2947, 2909, 2850, 1767, 1720, 1599, 1515, 1400, 1345, 1040, 1006; **^{1}H NMR** (300 MHz, $CDCl_3$) δ 8.23 (d, *J* = 8.6 Hz, 2H, *p*-nitrophenyl), 7.87 –7.71 (m, 4H, phthalimide), 7.50 (d, *J* = 8.6 Hz, 2H, *p*-nitrophenyl), 5.68 (ddd, *J* = 47.6, 7.1, 3.3 Hz, 1H, $C\underline{H}F$–CH_2–CH_2–CH_2), 3.81–3.76 (m, 2H, CHF–CH_2–CH_2–$NC\underline{H_2}$), 2.01–1.83 (m, 4H, CHF–$C\underline{H_2}$–$C\underline{H_2}$–NCH_2); **^{13}C{^{1}H} NMR** (75 MHz, $CDCl_3$) δ 168.4, 147.7, 134.10, 131.9, 126.0, 125.9, 123.8, 123.33, 92.3 (d, *J* = 173.2 Hz, CHF), 37.1, 34.5, 34.2, 24.0; **^{19}F NMR** (282 MHz, $CDCl_3$) δ –181.5 (ddd, *J* = 47.6, 27.6, 18.5 Hz, 1F, $CH\underline{F}$); **^{19}F{^{1}H} NMR** (282 MHz, $CDCl_3$) δ –181.5 (s, 1F, $CH\underline{F}$); **HRMS** (ESI, +ve) $C_{18}H_{15}FNO_4Na^+$ [MNa^+] requires *m/z* 365.0908, found 365.0904.

Acetanilide 43 and over-reduced product 62

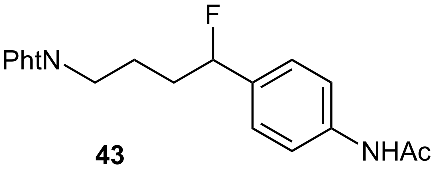

43

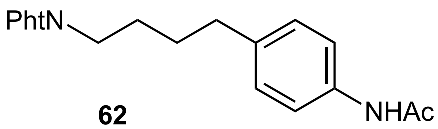

62

Pd/C (~8 mg) was added to a solution of **42** (48 mg, 0.140 mmol) in acetic anhydride (~5 mL). The mixture was stirred under H_2 atmosphere for 4 h. The reaction mixture was filtered through celite (ethyl acetate wash) and concentrated under reduced pressure. The crude product was purified by flash chromatography (dichloromethane → dichloromethane / ethyl acetate 14:3) to give acetanilide **43** as a colourless liquid (29 mg, 58%) and **62** as an off-white semisolid (7.5 mg, 15%).

Data for **43: IR** (neat) ν_{max} (cm^{-1}) 2928, 2330, 1770, 1795, 1711, 1508, 1396, 1369, 1172, 1039, 847, 720; **^{1}H NMR** (300 MHz, $CDCl_3$) δ 7.87−7.72 (m, 4H, phthalimide), 7.54 (d, *J* = 8.6 Hz, 2H, *p*-nitrophenyl), 7.43 (d, *J* = 8.6 Hz, 2H, *p*-nitrophenyl), 5.64 (ddd, *J* = 44.4, 7.7, 3.5 Hz, 1H, CHF), 3.81−3.75 (m, 2H, NCH_2), 2.22 (s, 3H, $COCH_3$), 2.01−1.88 (m, 4H, CHF−CH_2−CH_2); **^{13}C{^{1}H} NMR** (75 MHz, $CDCl_3$) δ 168.4 (phthalimide CO), 167.89, 139.1, 134.0, 132.0, 126.3, 123.3, 93.0 (d, *J* = 171.1 Hz, CHF), 58.5, 37.3, 21.54, 21.0; **^{19}F NMR** (282 MHz, $CDCl_3$) δ −177.5 (m, 1F, *p*-nitrophenyl−CHF); **^{19}F{^{1}H} NMR** (282 MHz, $CDCl_3$) δ −177.5 (m, 1F, *p*-nitrophenyl−CHF); **HRMS** (ESI, +ve) $C_{20}H_{19}FN_2O_3Na^+$ [MNa^+] requires *m/z* 377.1227, found 377.1224.

Data for **62**: **IR** (neat) ν_{max} (cm^{-1}) 2923, 2854, 1769, 1720, 1601, 1533, 1512, 1395, 1368, 1034, 795, 719; **^{1}H NMR** (300 MHz, $CDCl_3$) δ 7.88−7.84 (m, 2H, phthalimide), 7.75−7.71 (m, 2H, phthalimide), 7.39 (d, *J* = 8.1 Hz, 2H, *p*-nitrophenyl), 7.13 (d, *J* = 8.1 Hz, 2H, *p*-nitrophenyl), 3.72 (t, *J* = 6.6 Hz, 2H, NCH_2), 2.63 (t, *J* = 6.9 Hz, 2H, *p*-nitrophenyl CH_2), 2.19 (s, 3H, $COCH_3$), 1.80−1.65 (m, 4H, CH_2−CH_2); **^{13}C{^{1}H} NMR** (75 MHz, $CDCl_3$) δ 168.4, 138.12, 133.9, 133.8, 132.1, 128.9, 123.1, 120, 37.3, 34.7, 29.7, 28.6, 28; **HRMS** (ESI, +ve) $C_{20}H_{20}N_2O_3Na^+$ [MNa^+] requires *m/z* 359.1366, found 359.1363.

Carboxylic acid 44

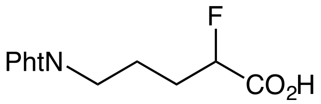

To a stirred mixture of **43** (14 mg, 0.039 mmol), dichloromethane (0.5 mL), acetonitrile (0.5 mL) and water (0.7 mL) was added sodium metaperiodate (168 mg, 0.788 mmol) followed by ruthenium chloride hydrate (~4 mg). The resulting mixture was stirred at rt for 5 days. The mixture was filtered through celite (ethyl acetate wash) and the filtrate was concentrated *in vacuo*. The crude product was subjected to flash chromatography (98:0:2→80:18:2 $CHCl_3$/MeOH/AcOH) to give carboxylic acid **44** as a brown oil (3.1 mg, 31%); **IR** (neat) ν_{max} (cm^{-1}) 3684, 3669, 2983, 2969, 2900,1709, 1400, 1240, 1100, 892; **^{1}H NMR** (300 MHz, $CDCl_3$) δ 7.90–7.86 (m, 2H, phthalimide), 7.77–7.73 (m, 2H, phthalimide), 5.05 (ddd, *J* = 48.0, 7.6, 3.4 Hz, 1H, C<u>H</u>F–COOH), 3.80 (t, 2H, *J* = 48.0, NC<u>H</u>$_2$), 2.10–1.92 (m, 4H, C<u>H</u>$_2$–C<u>H</u>$_2$); **^{13}C {^{1}H} NMR** (75 MHz, $CDCl_3$) δ 168.4 (phthalimide CO), 134.1 (phthalimide ArC), 132.0 (phthalimide ArC), 123.3, 87.9 (d, *J* = 187.3 Hz, 1CF), 37.1, 29.5 (d, *J* = 21.0 Hz, 1C), 23.7 (d, *J* = 3.1 Hz, 1C); **^{19}F NMR** (282 MHz, $CDCl_3$) δ –191.5 (m, 1F, CH<u>F</u>); **^{19}F{^{1}H} NMR** (282 MHz, $CDCl_3$) δ –191.5 (s, 1F, CH<u>F</u>); **HRMS** (ESI, +ve) $C_{13}H_{12}FNO_4Na^+$ [MNa^+] requires *m/z* 288.0642, found 288.0640.

Spectra of novel compounds

^{1}H NMR (600 MHz, D_2O) of **6a**

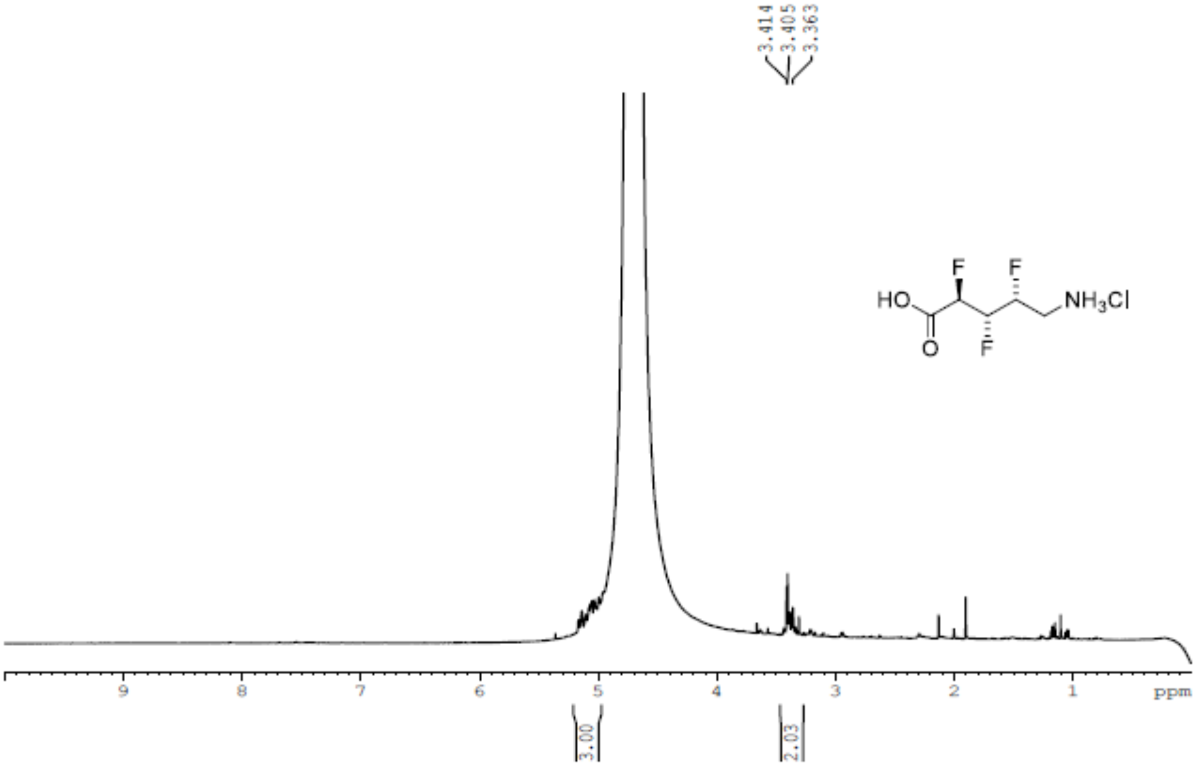

^{19}F NMR (564 MHz, D_2O) of **6a**

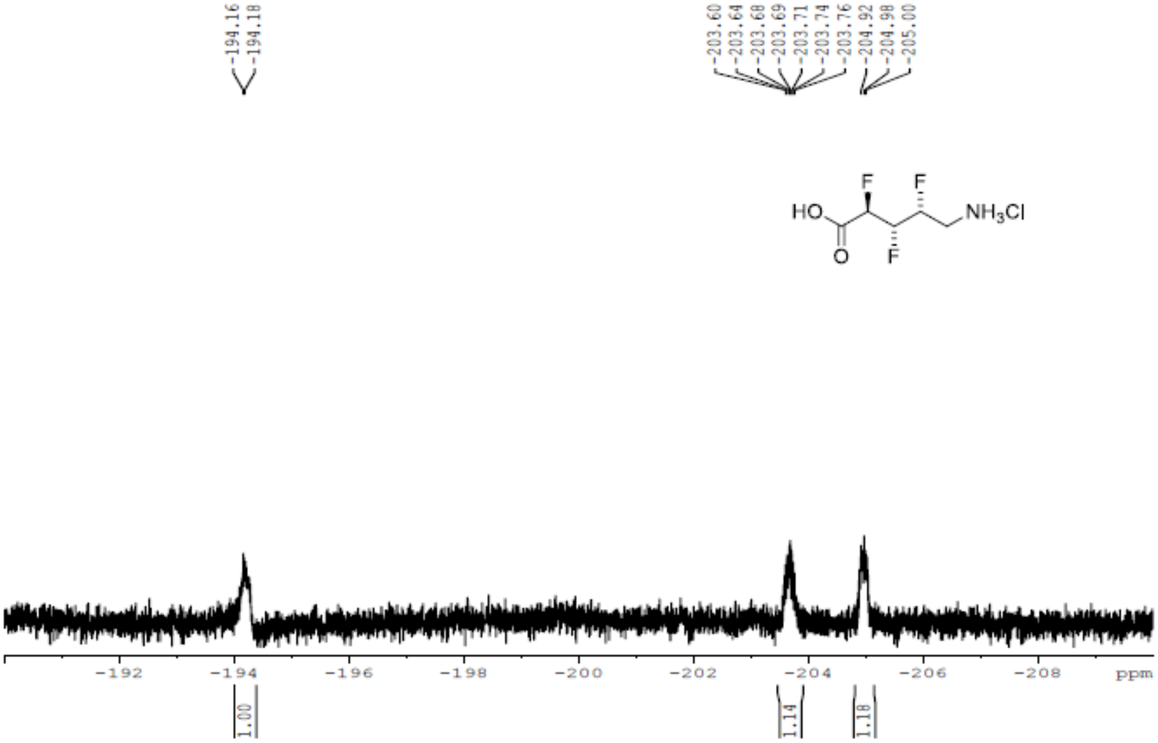

$^{19}F\{^{1}H\}$ NMR (564 MHz, D_2O) of **6a**

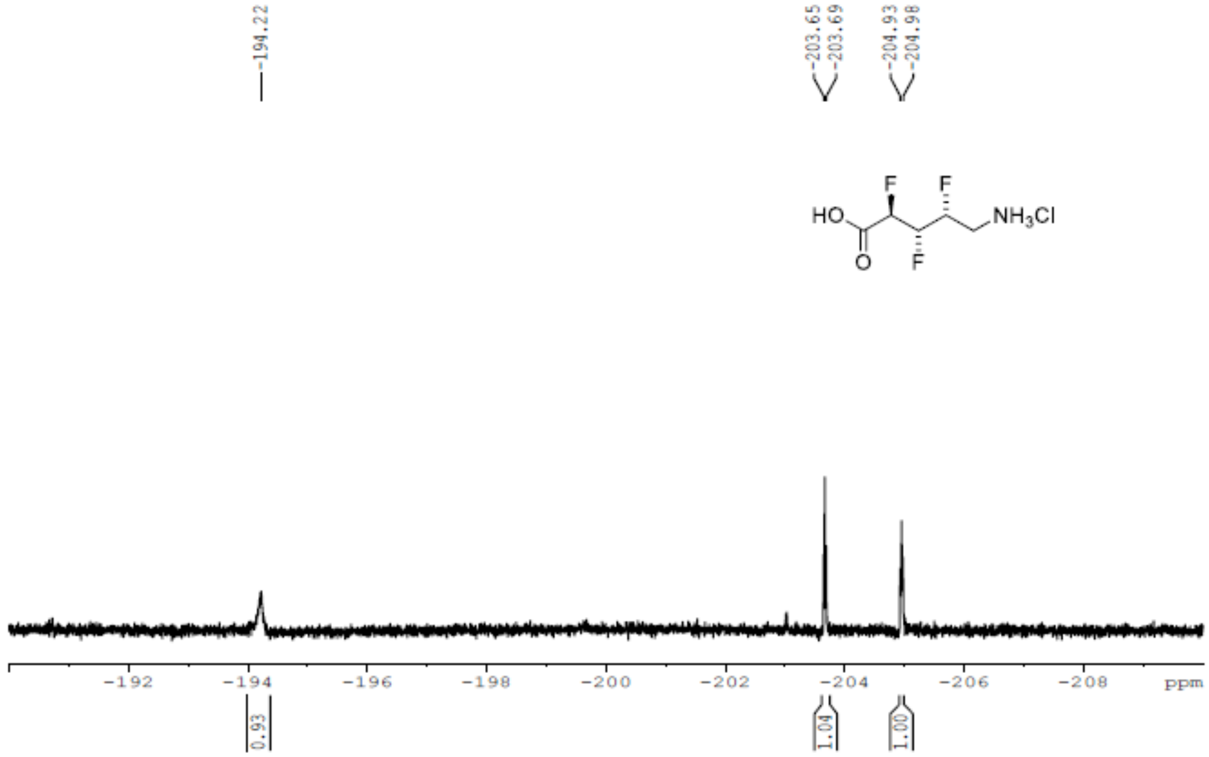

^{1}H NMR (600 MHz, D_2O) of **6b**

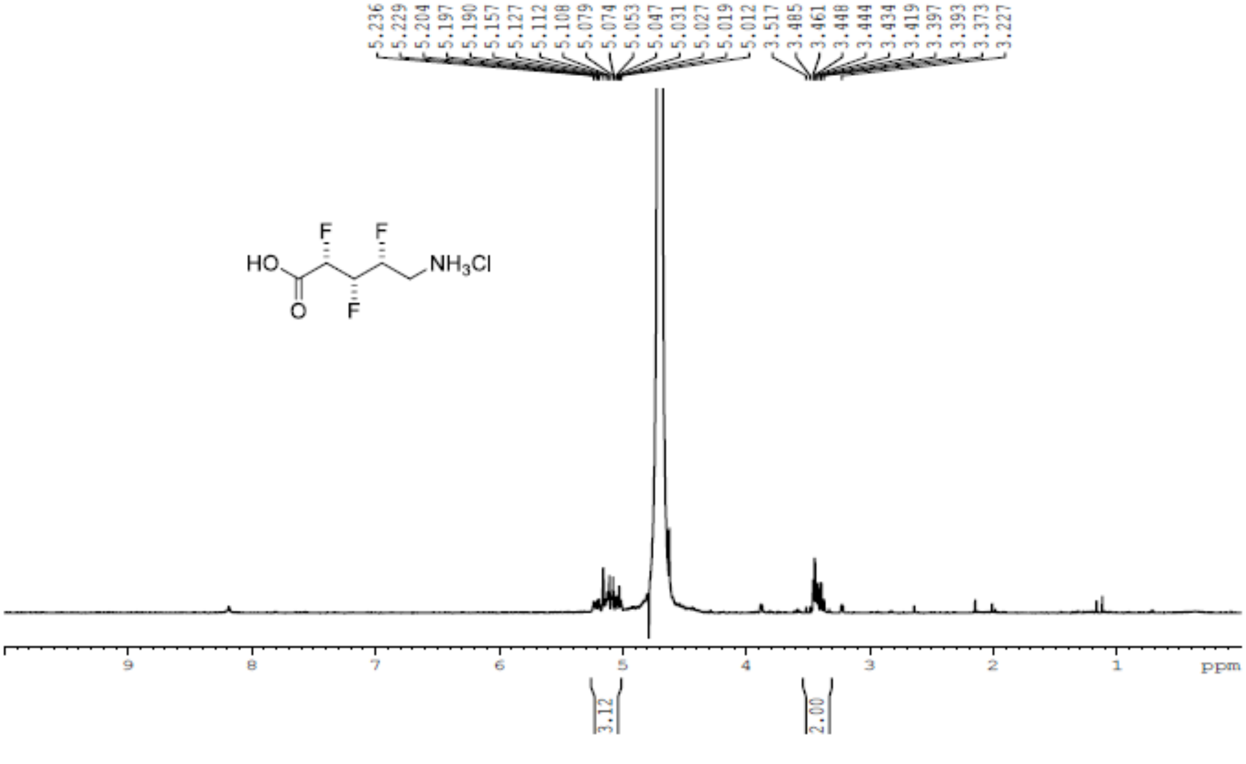

$^{13}C\{^{1}H\}$ NMR (75 MHz, D_2O) of **6b**

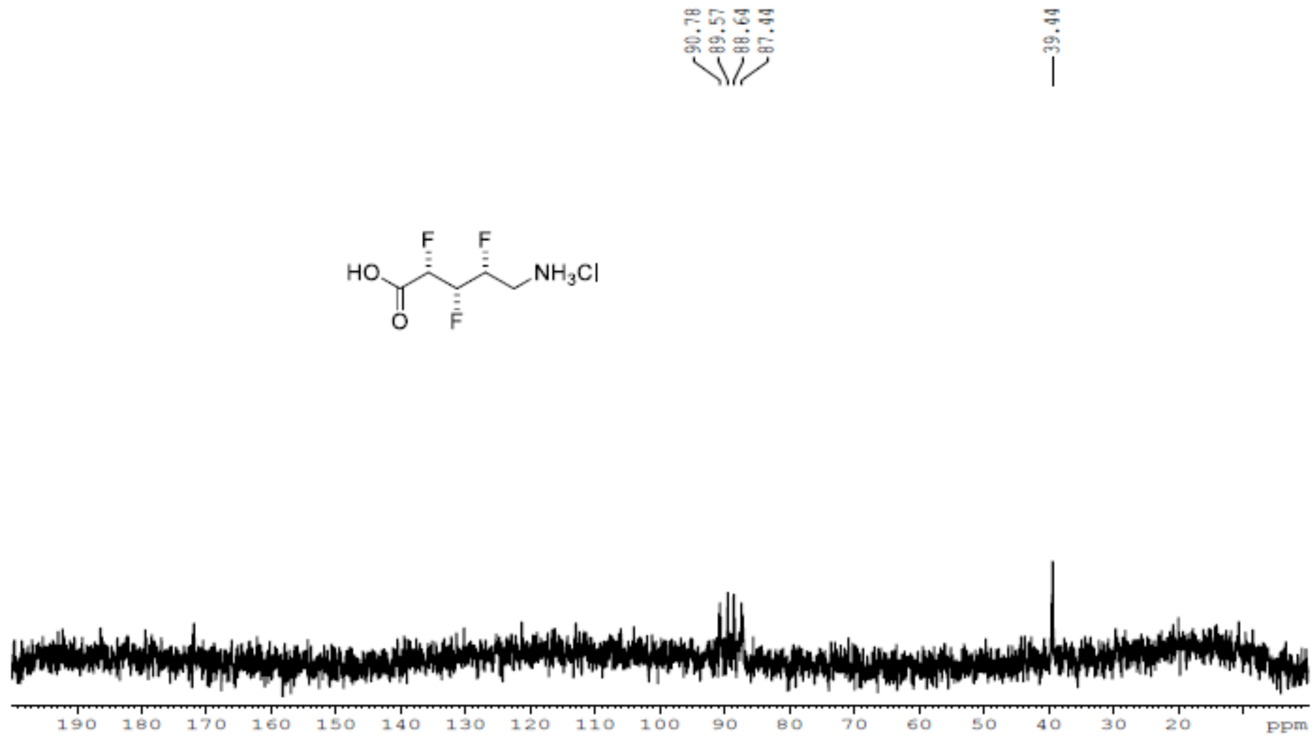

^{19}F NMR (282 MHz, D_2O) of **6b**

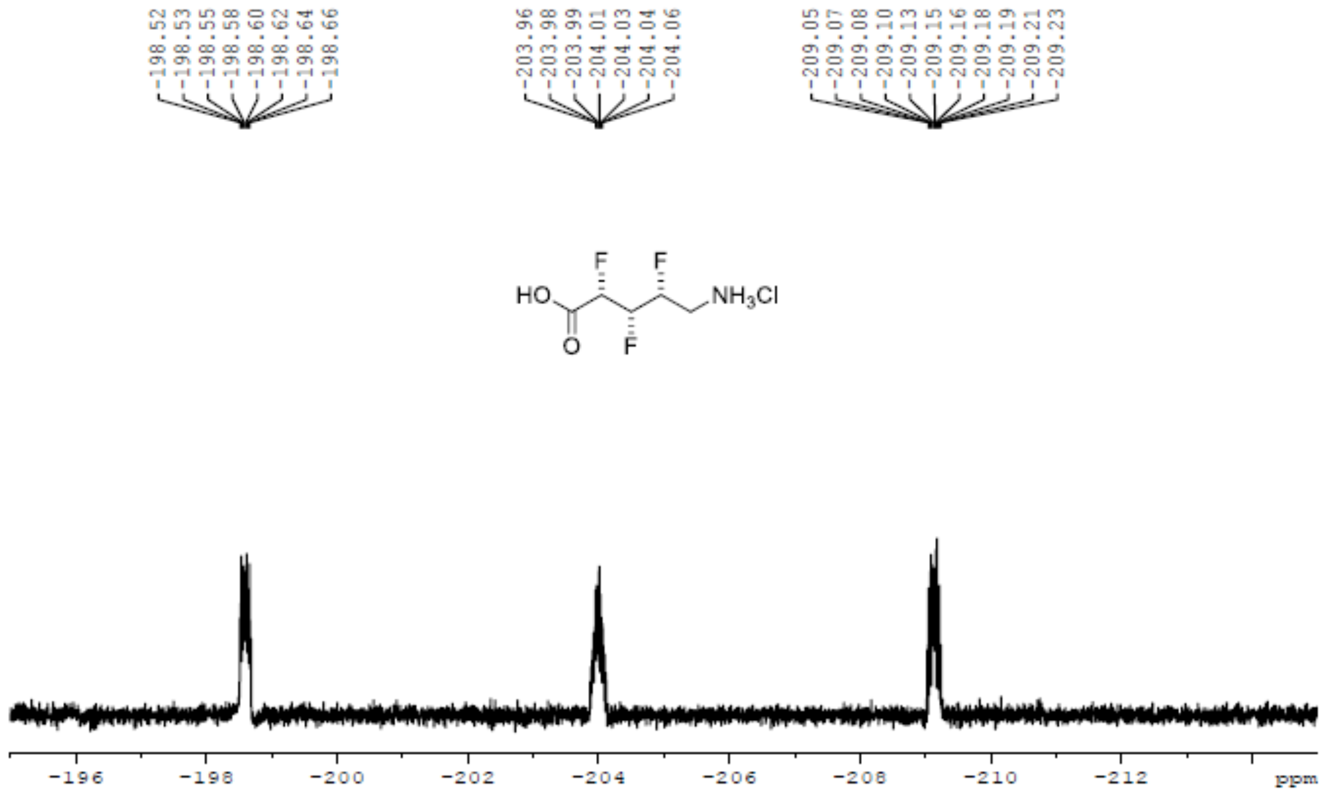

$^{19}F\{^1H\}$ NMR (282 MHz, D_2O) of **6b**

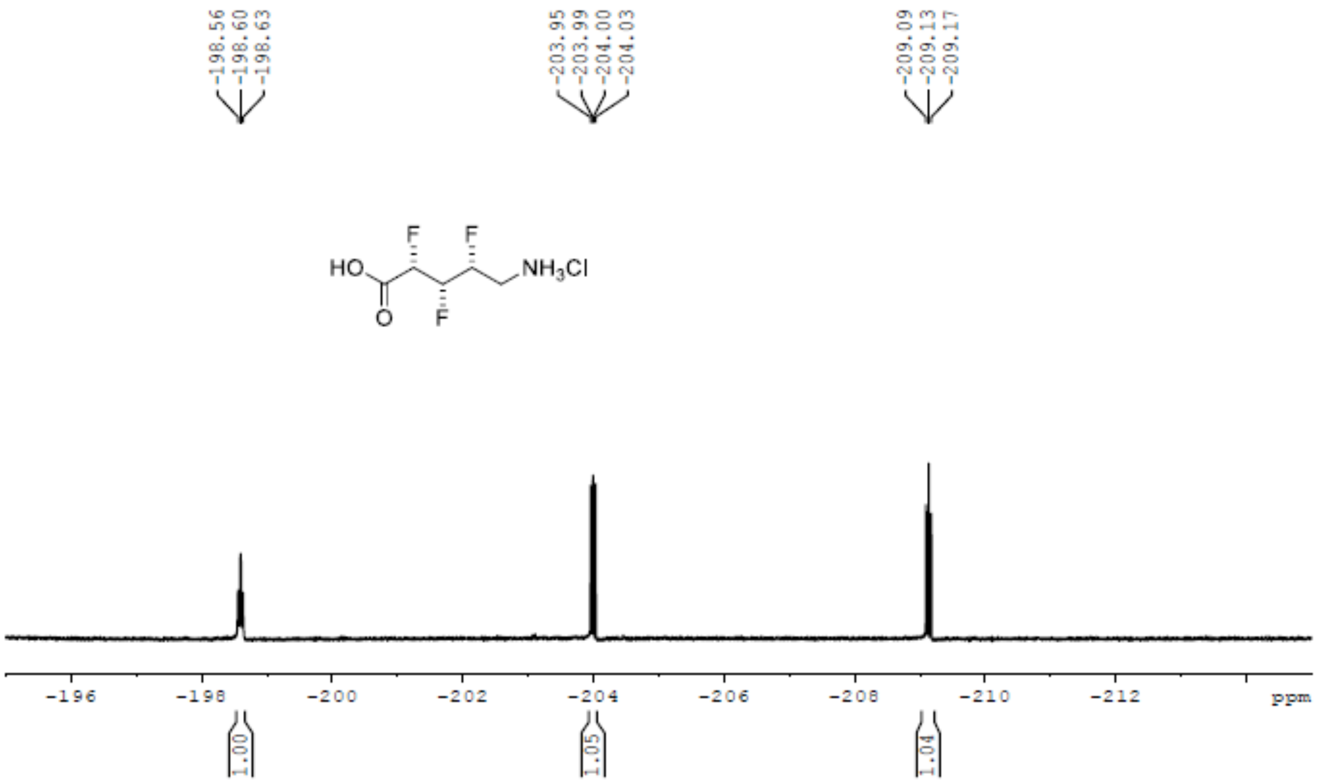

^1H NMR (300 MHz, $CDCl_3$) of **11**

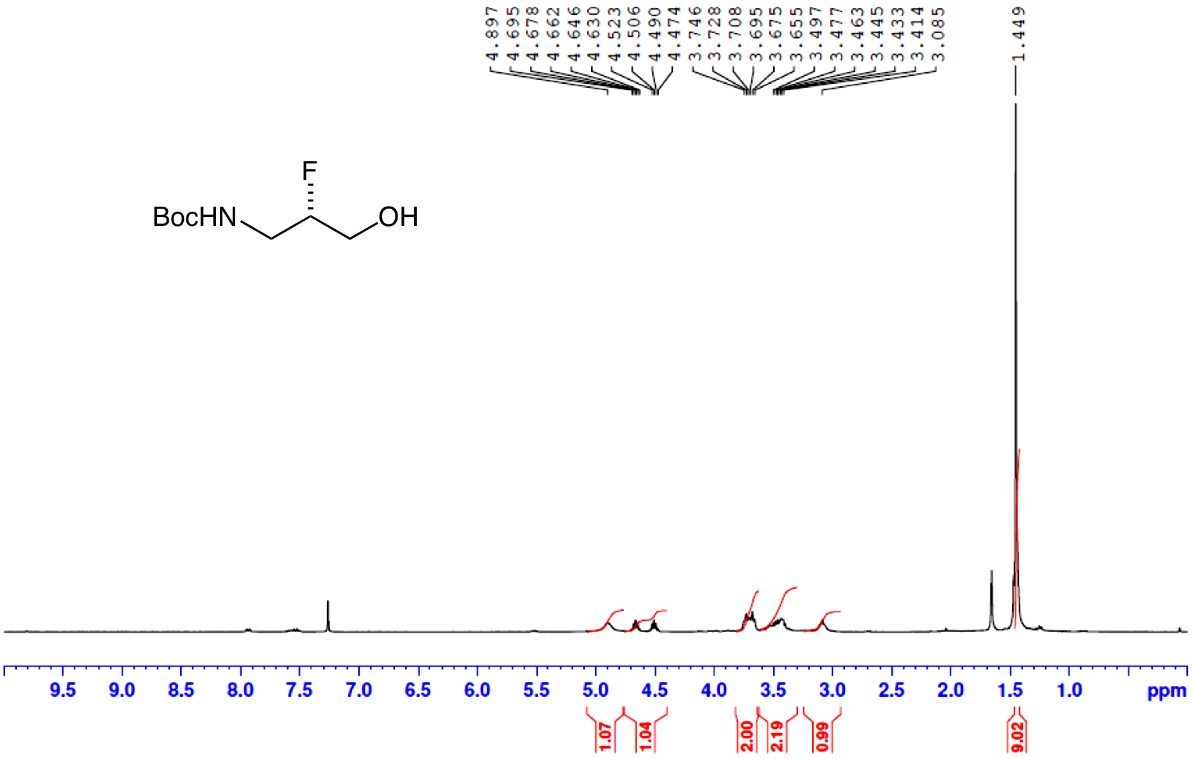

$^{13}C\{^1H\}$ NMR (75 MHz, $CDCl_3$) of **11**

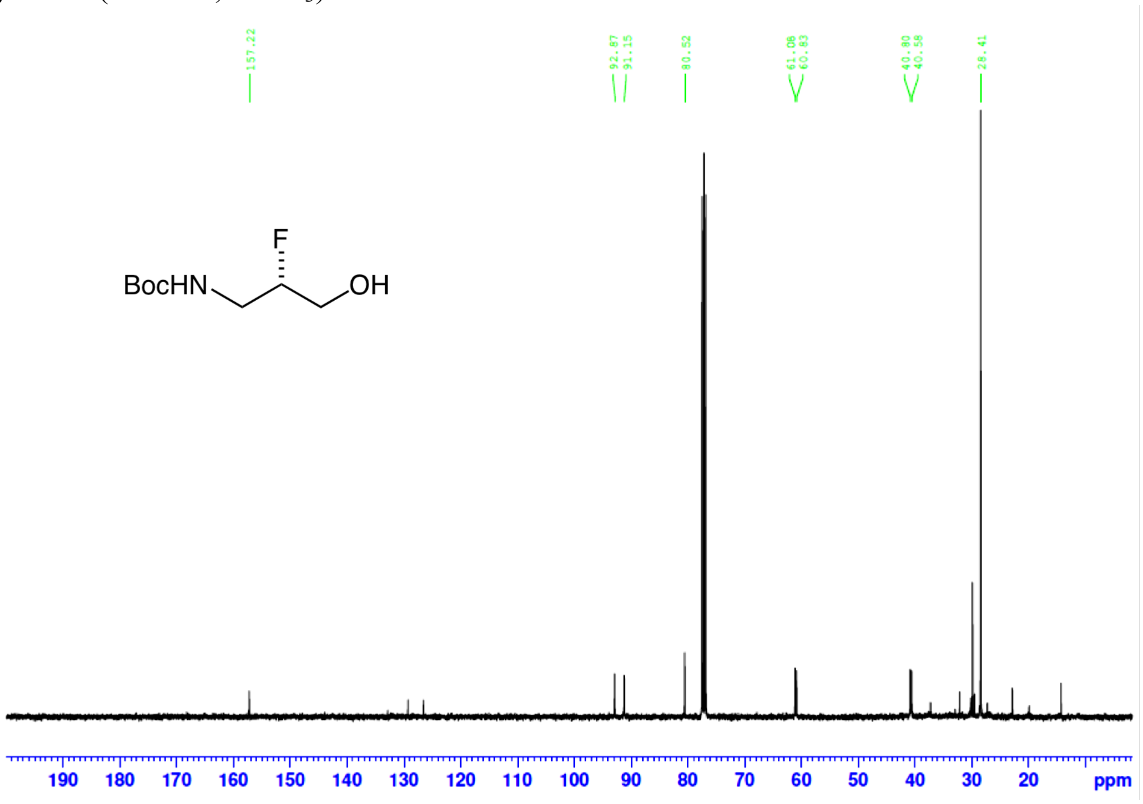

^{19}F NMR (282 MHz, $CDCl_3$) of **11**

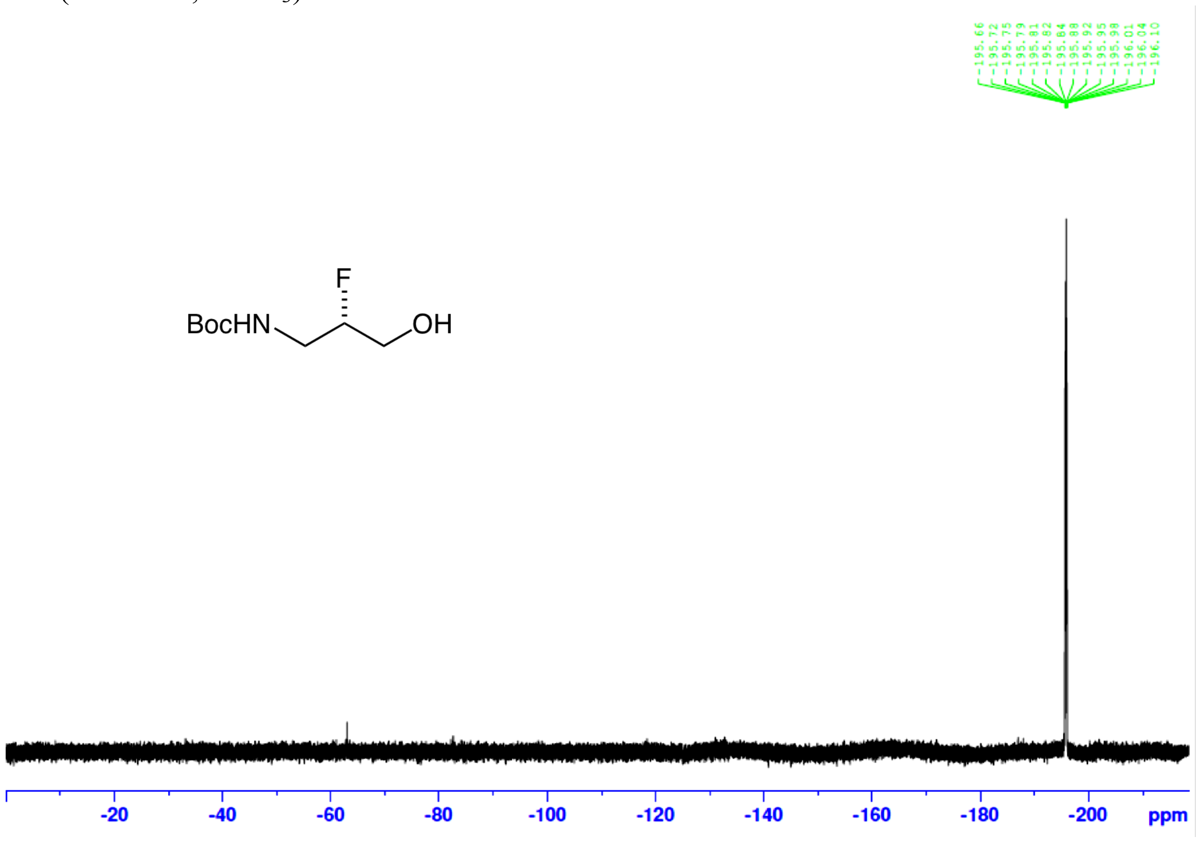

$^{19}F\{^{1}H\}$ NMR of Mosher's ester of **11**

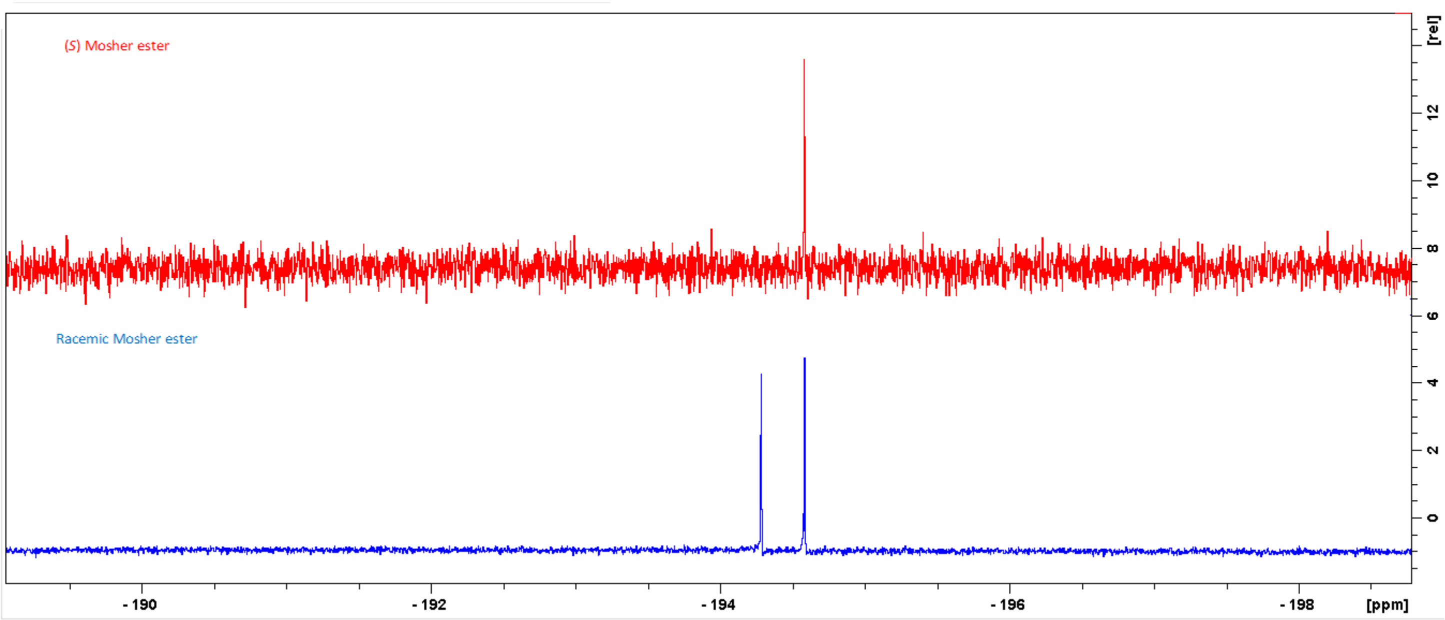

^{1}H NMR (300 MHz, CD_3OD) of **12**

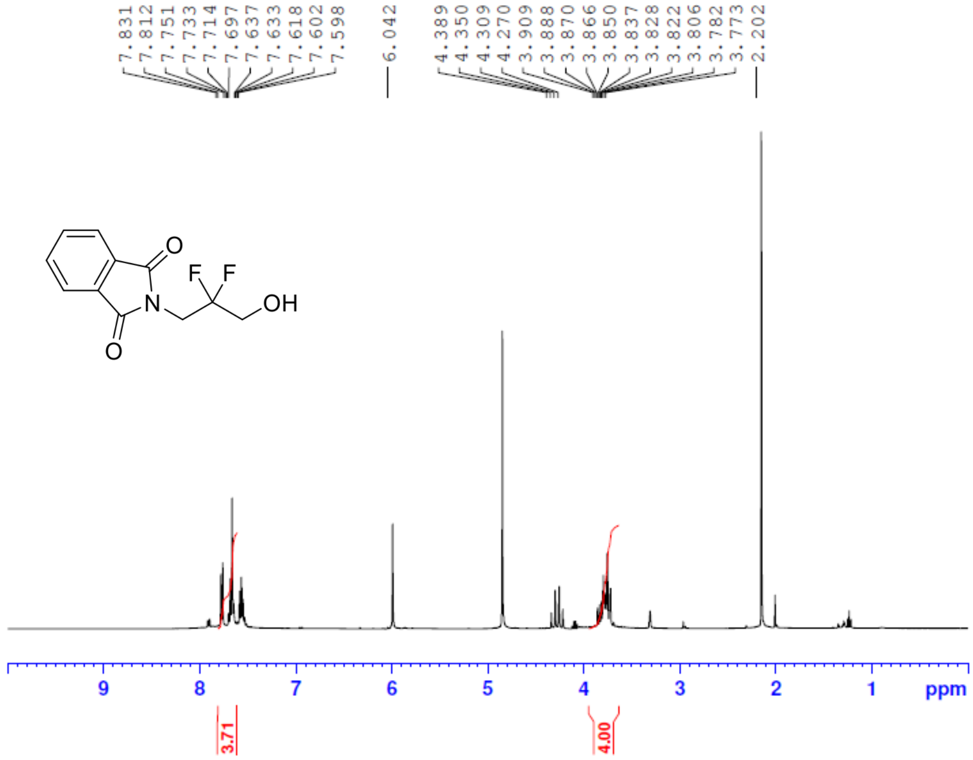

^{19}F NMR (282 MHz, CD_3OD) of **12**

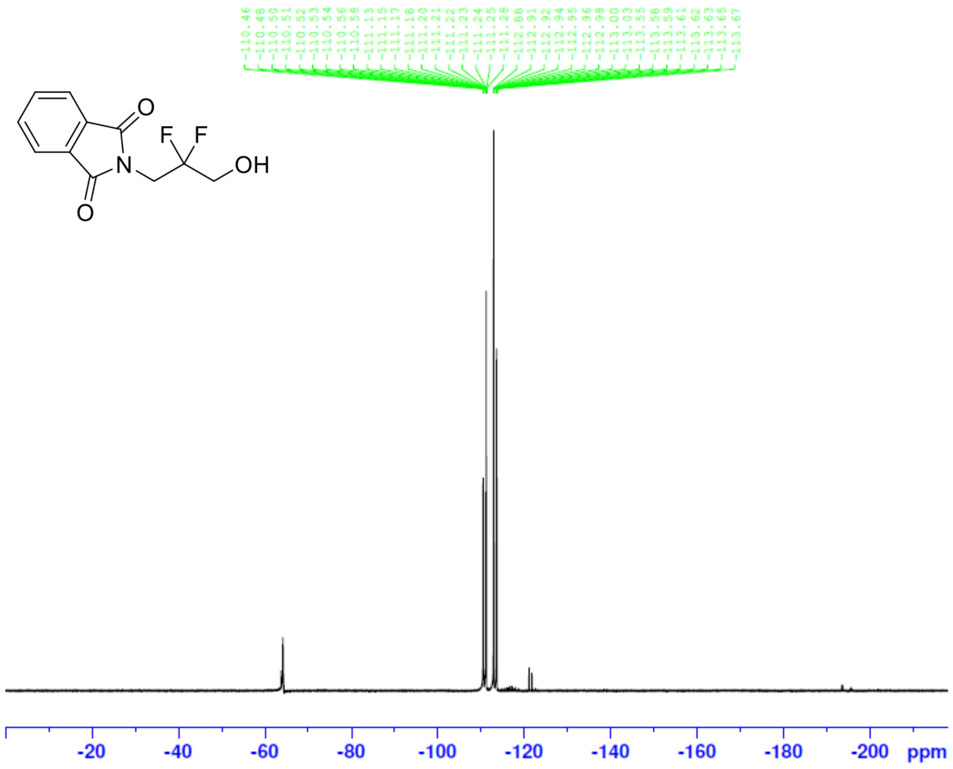

^{1}H NMR (300 MHz, $CDCl_3$) of **14**

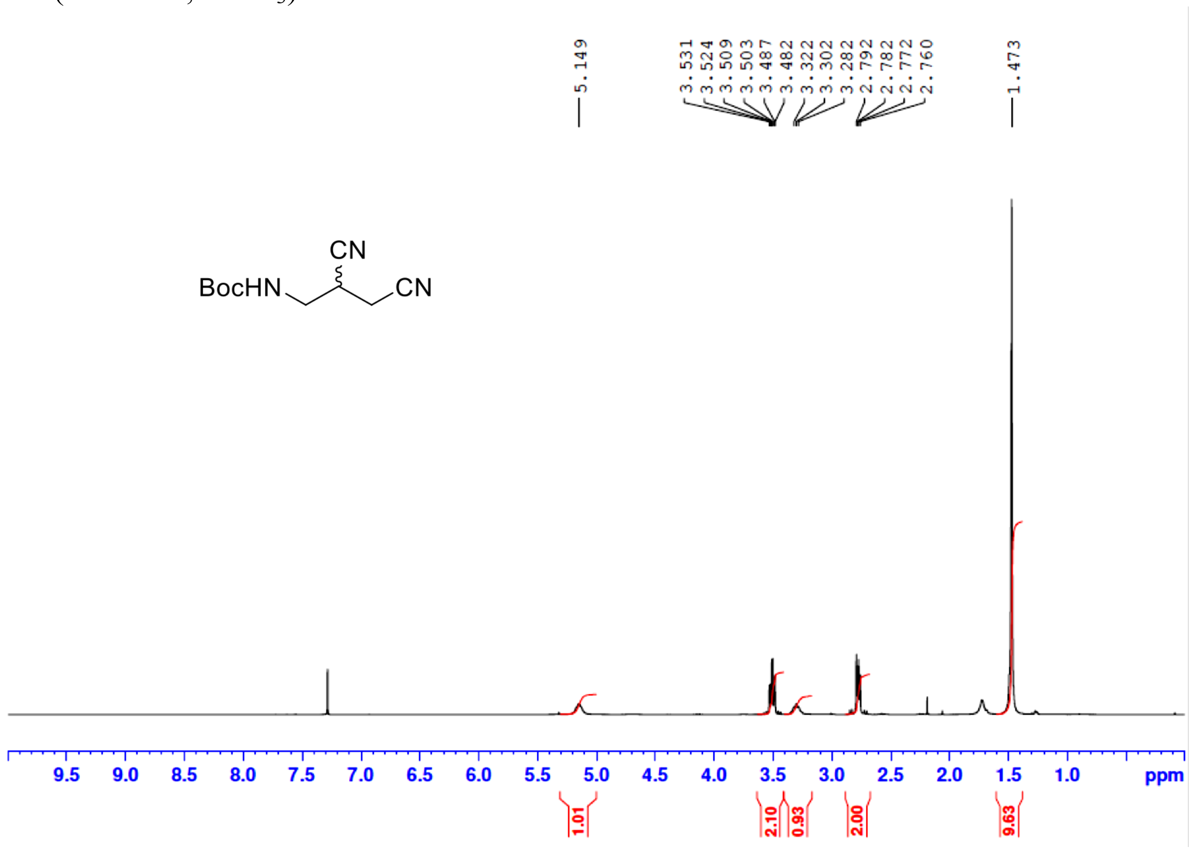

$^{13}C\{^{1}H\}$ NMR (282 MHz, $CDCl_3$) of **14**

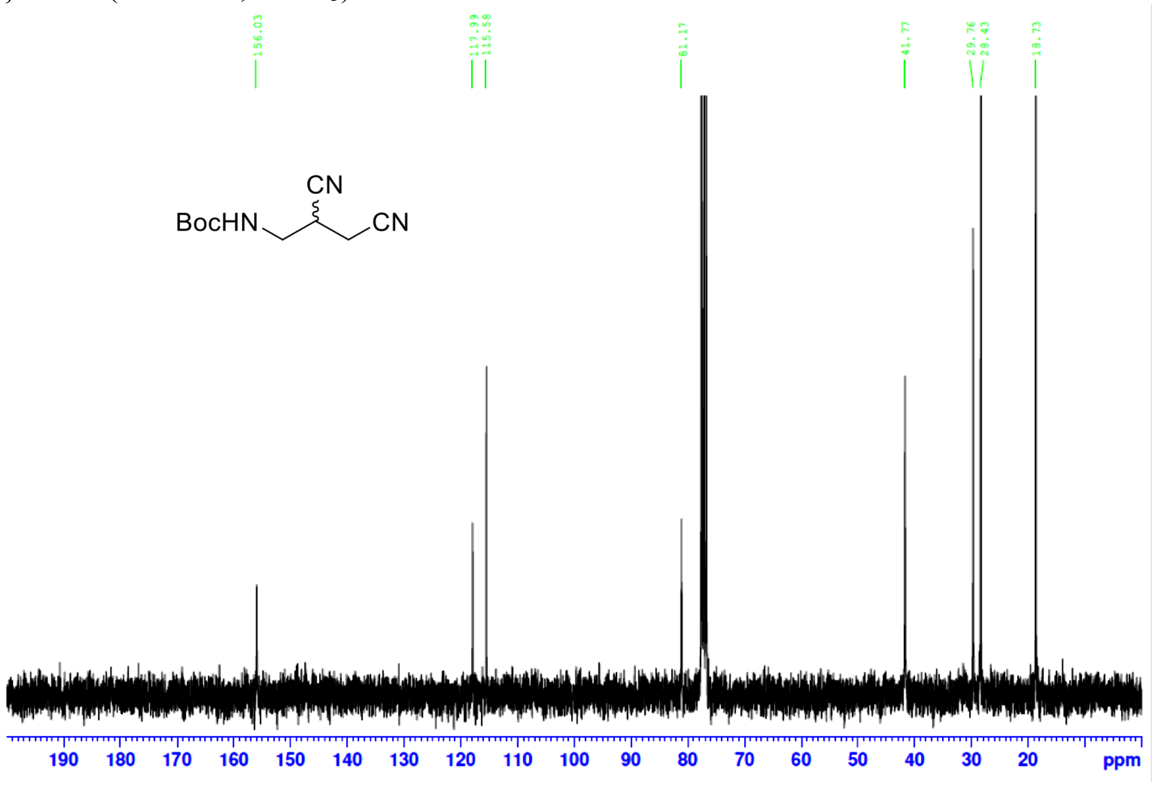

^{1}H NMR (300 MHz, $CDCl_3$) of **17** + **51**

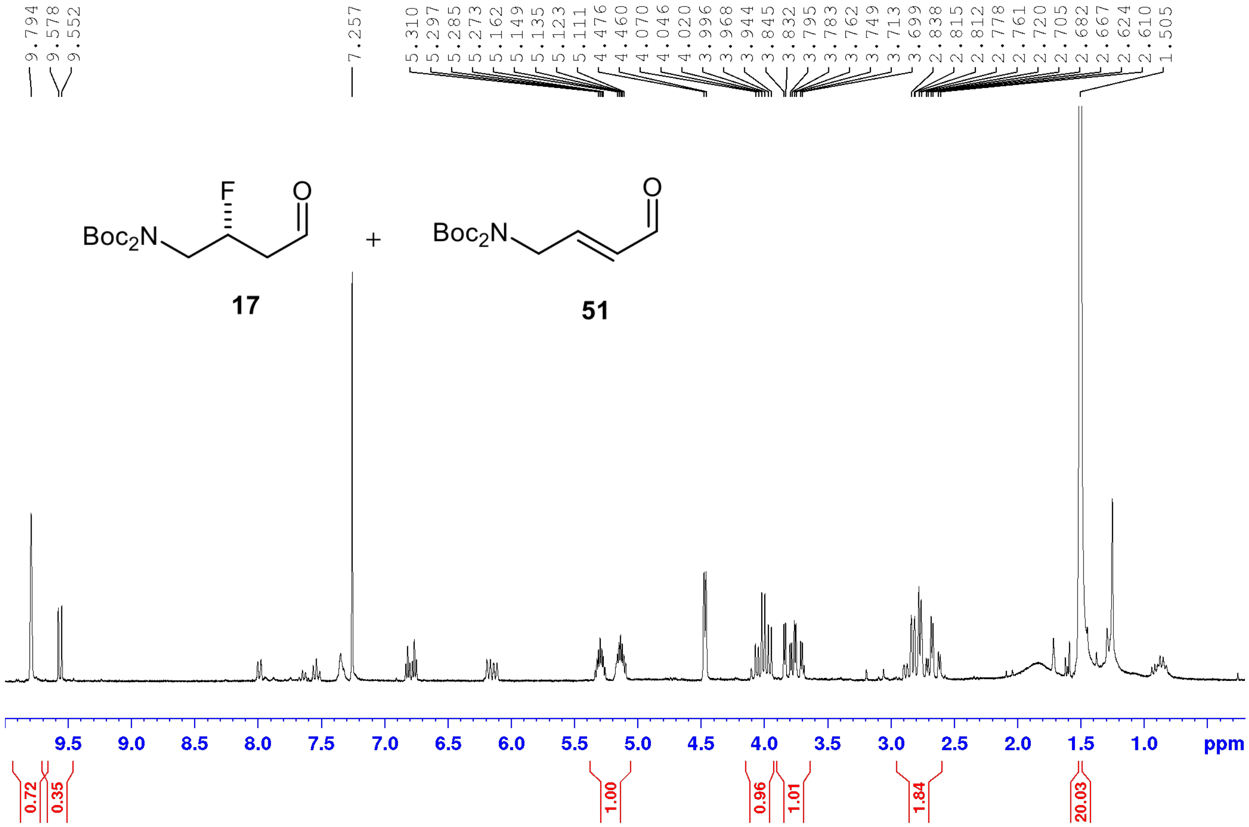

^{19}F NMR (280 MHz, $CDCl_3$) of **17 + 51**

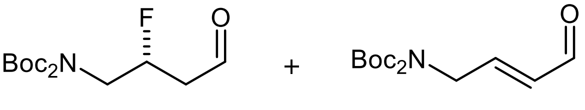

17 **51**

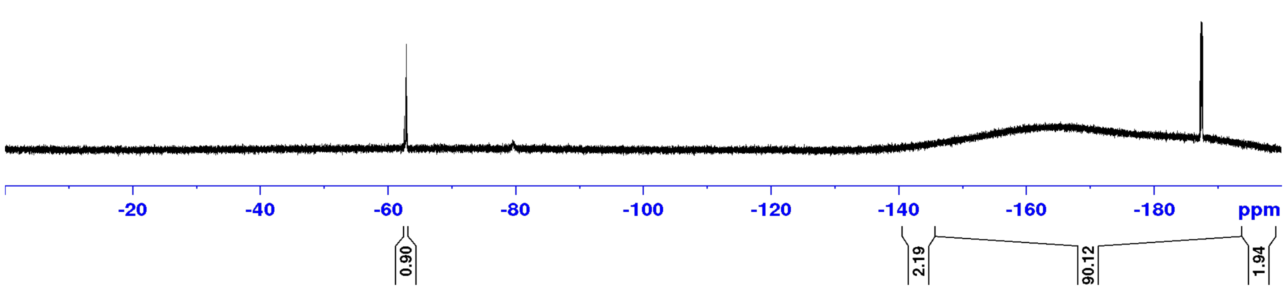

^{1}H NMR (400 MHz, $CDCl_3$) of **18** (crude)

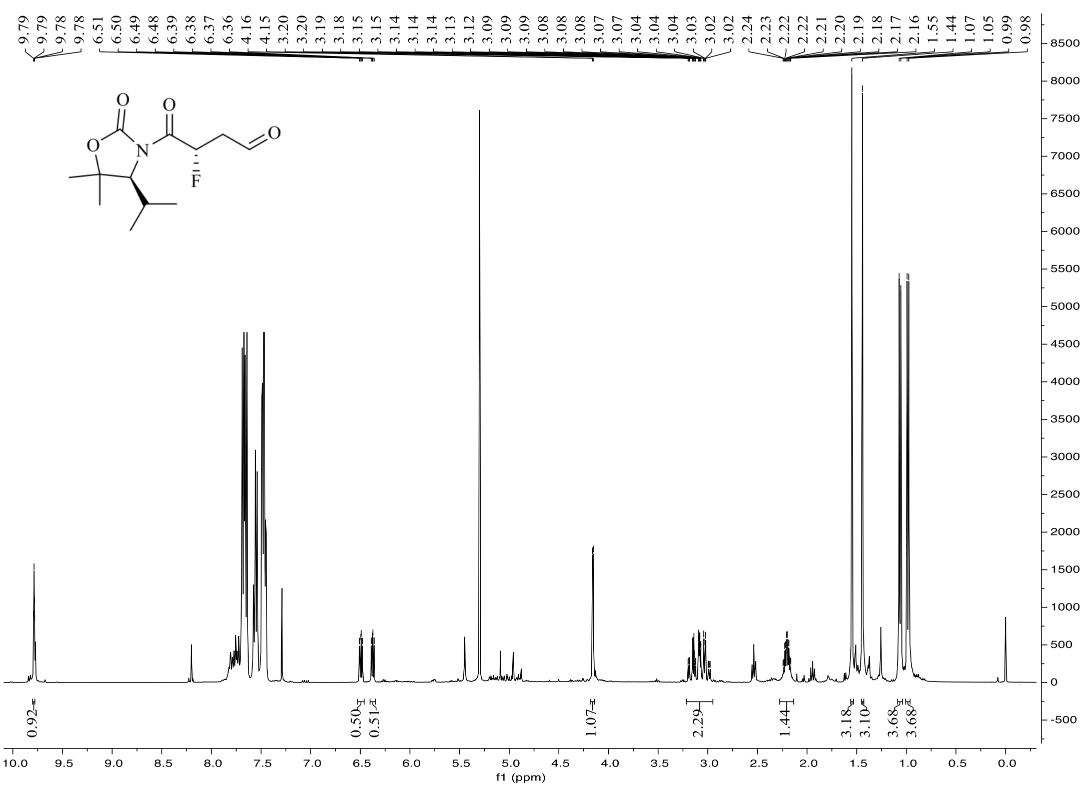

$^{19}F\{^{1}H\}$ NMR (367 MHz, $CDCl_3$) of **18** (crude)

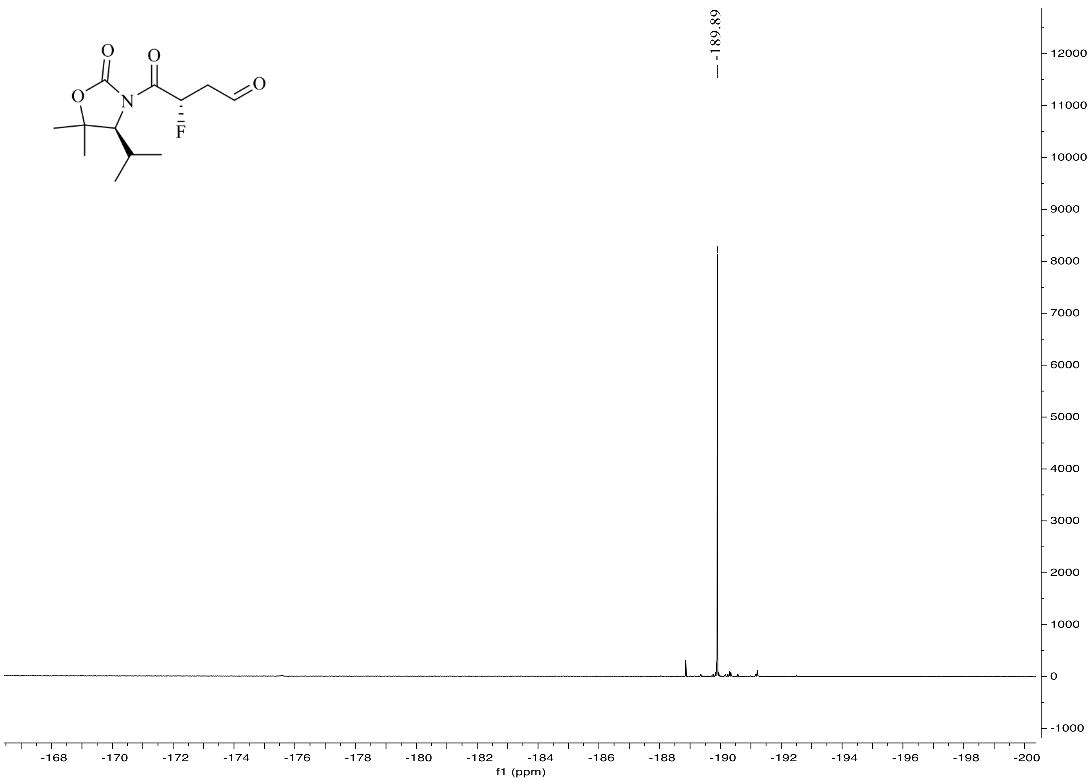

^{19}F NMR (367 MHz, $CDCl_3$) of **18** (crude)

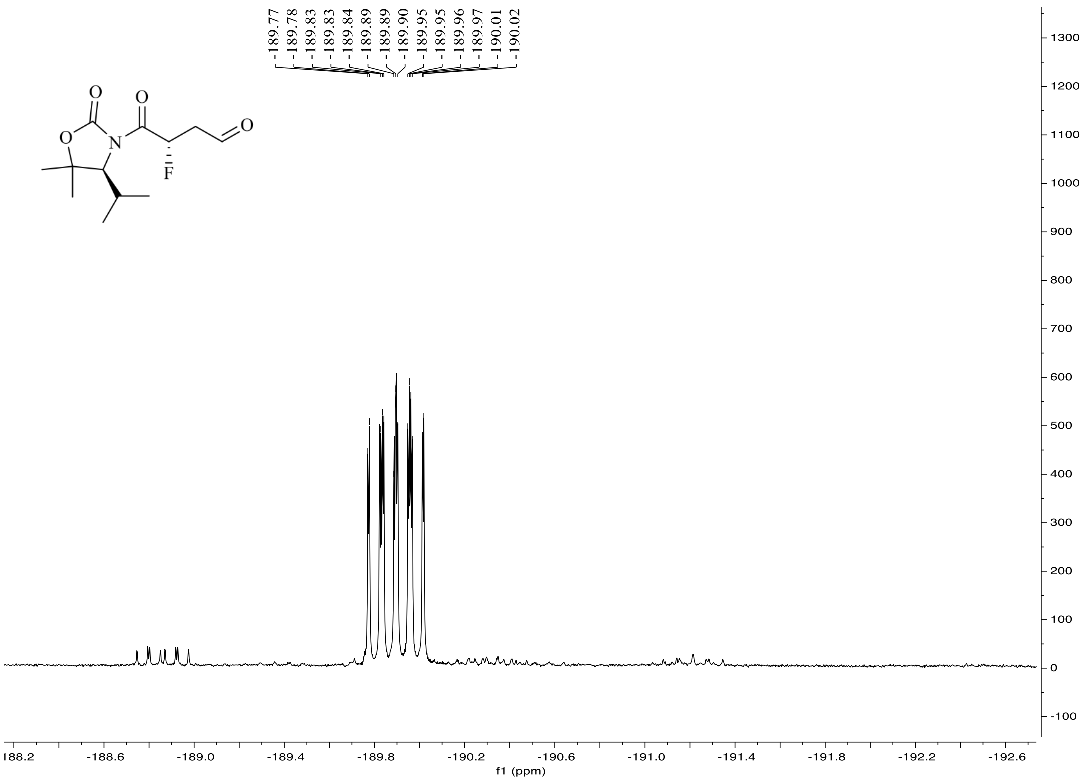

^{1}H NMR (400 MHz, $CDCl_3$) of **19**

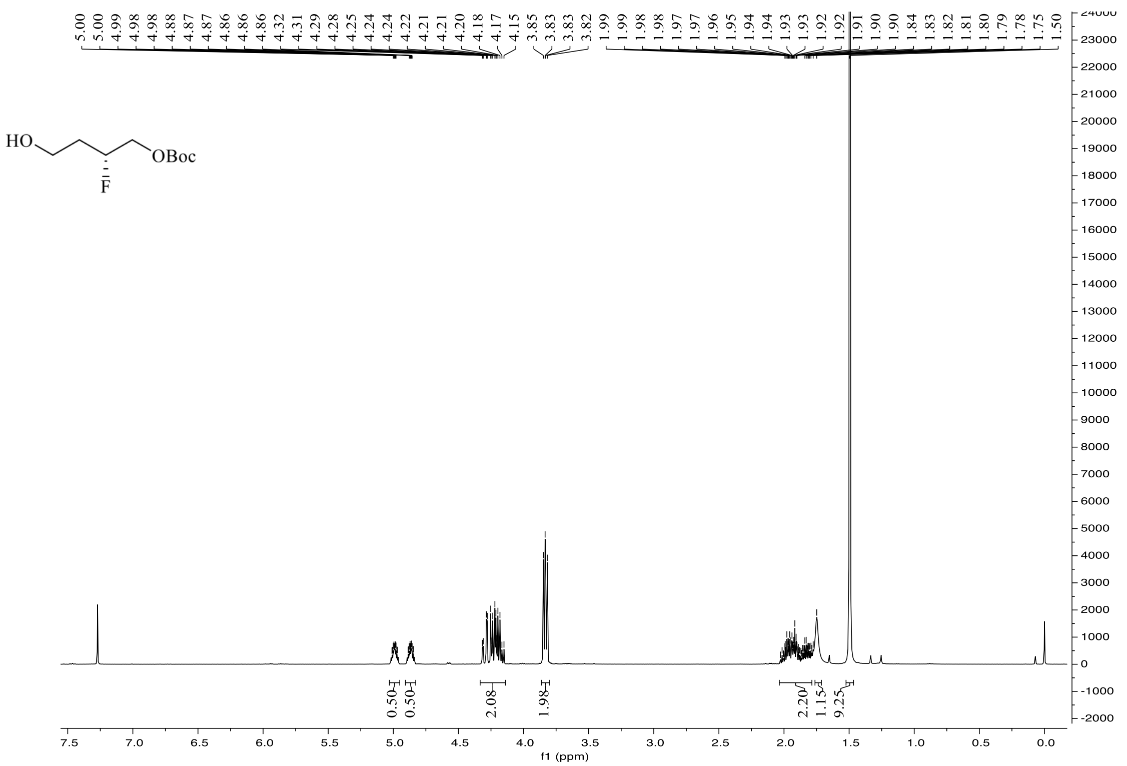

^{13}C{^{1}H} NMR (100 MHz, $CDCl_3$) of **19**

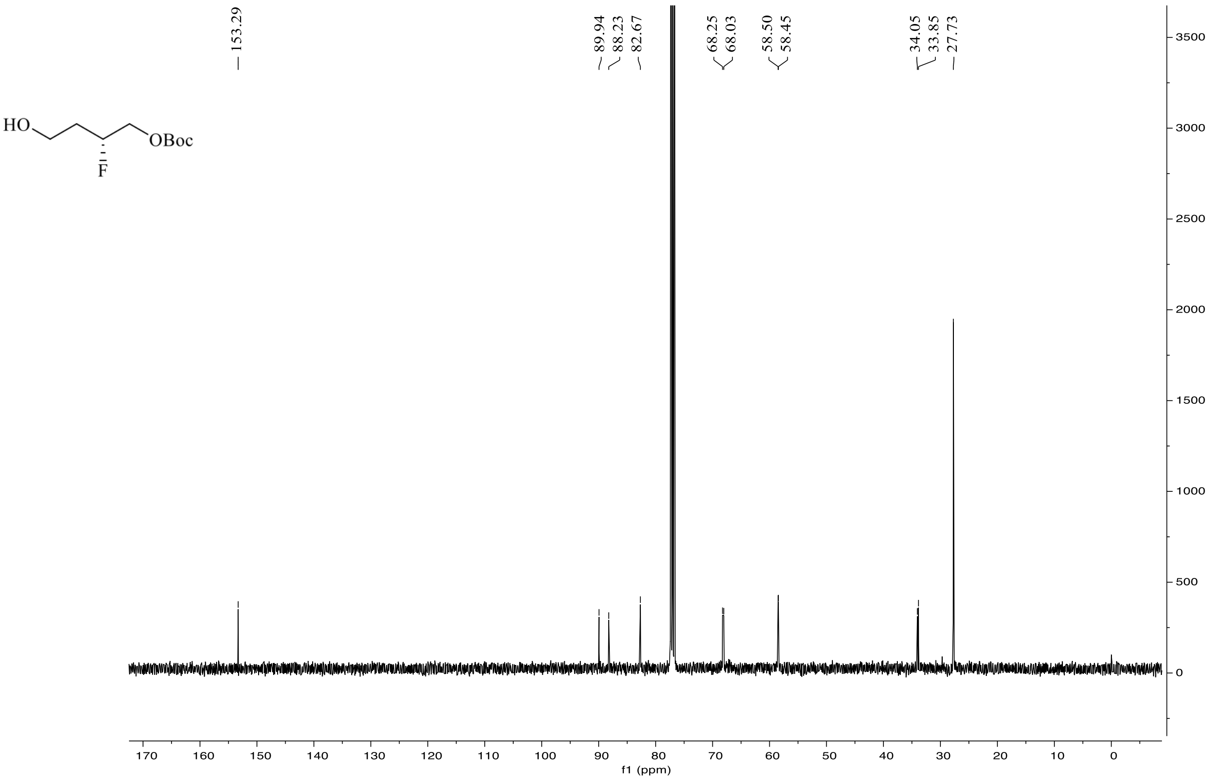

$^{19}F\{^1H\}$ NMR (367 MHz, $CDCl_3$) of **19**

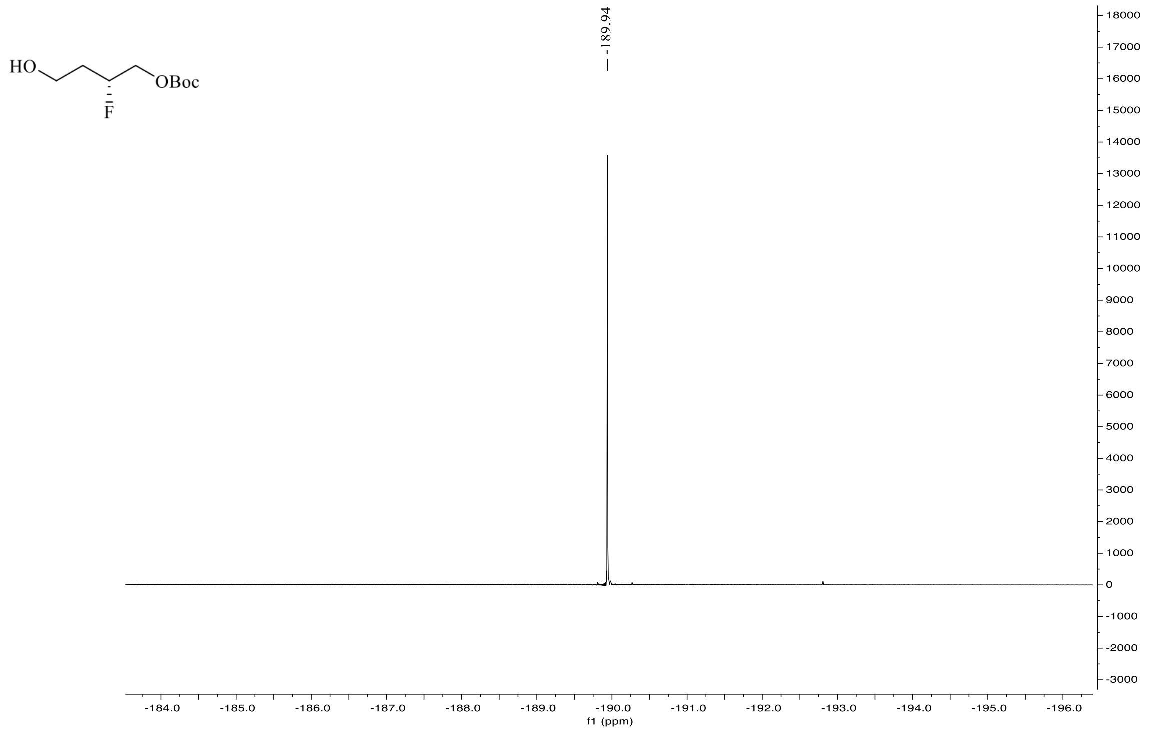

^{19}F NMR (367 MHz, $CDCl_3$) of **19**

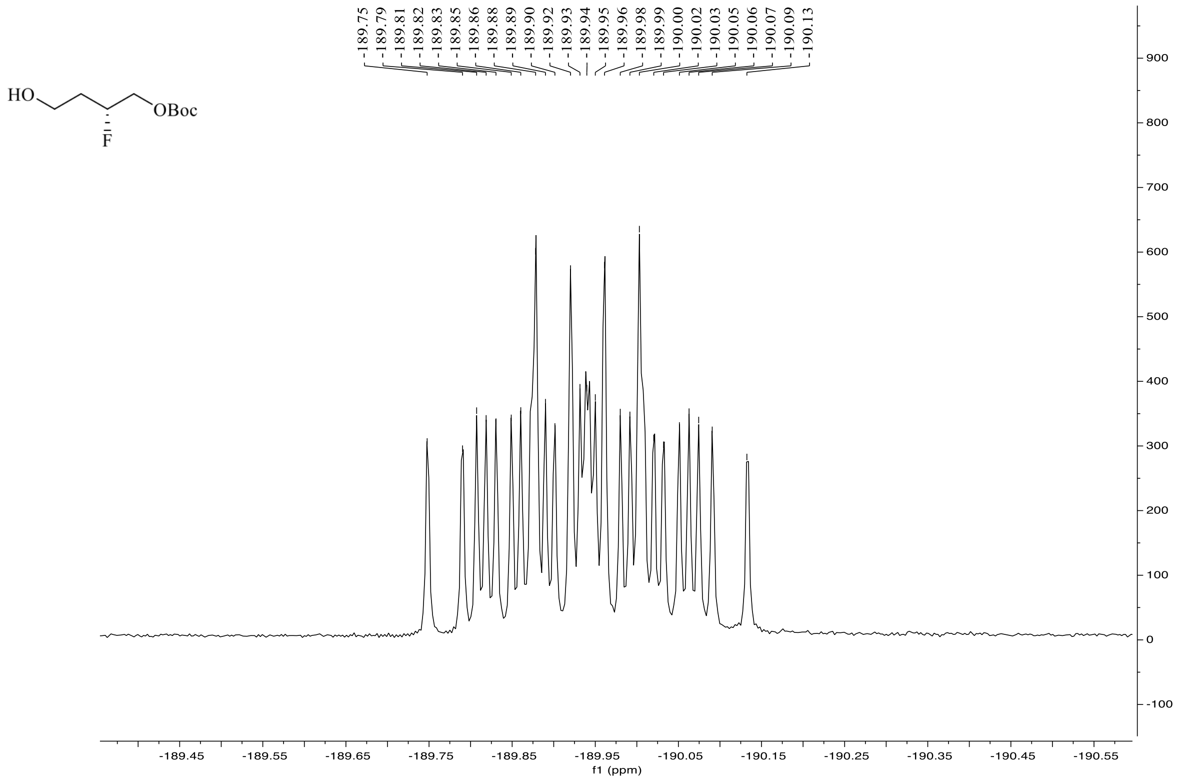

^{1}H NMR (300 MHz, $CDCl_3$) of **20**

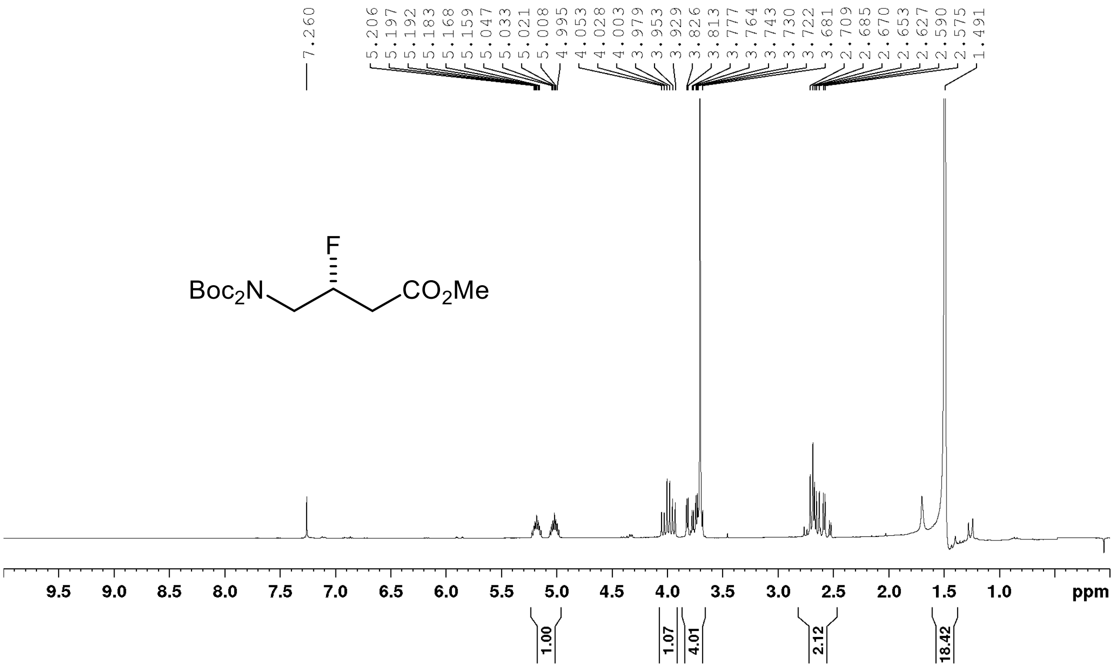

^{13}C{^{1}H} NMR (300 MHz, $CDCl_3$) of **20**

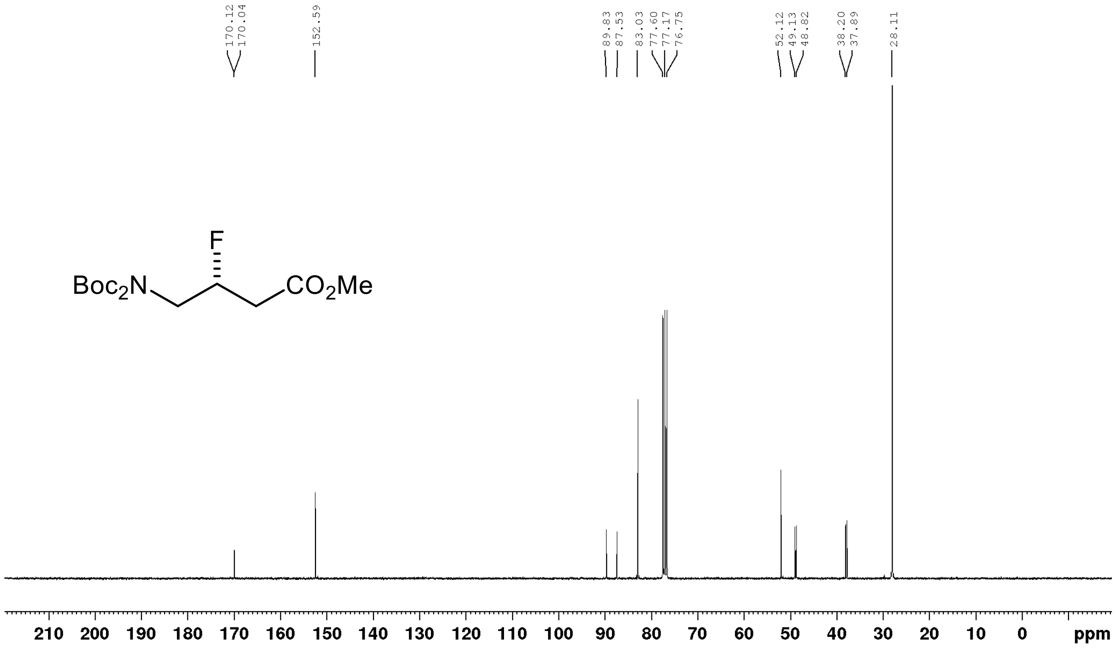

^{19}F NMR (282 MHz, $CDCl_3$) of **20**

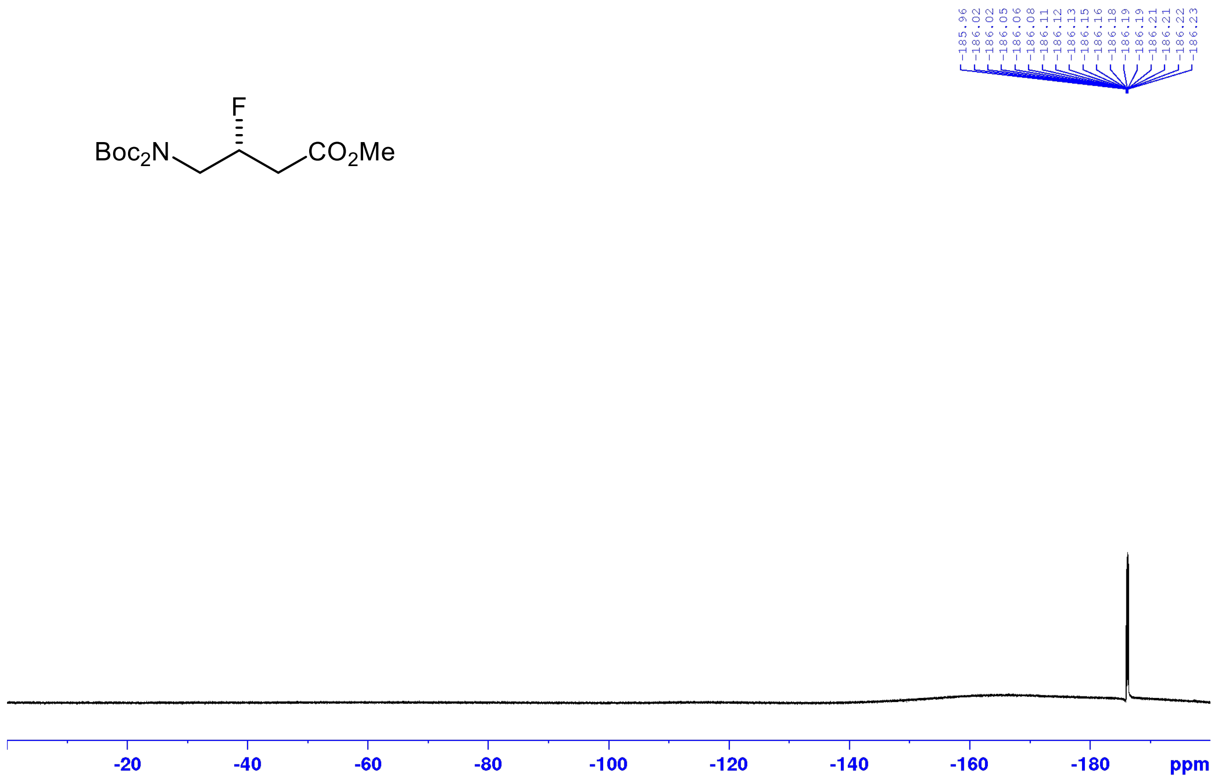

^{19}F{^{1}H} NMR (282 MHz, CDCl3) of **20**

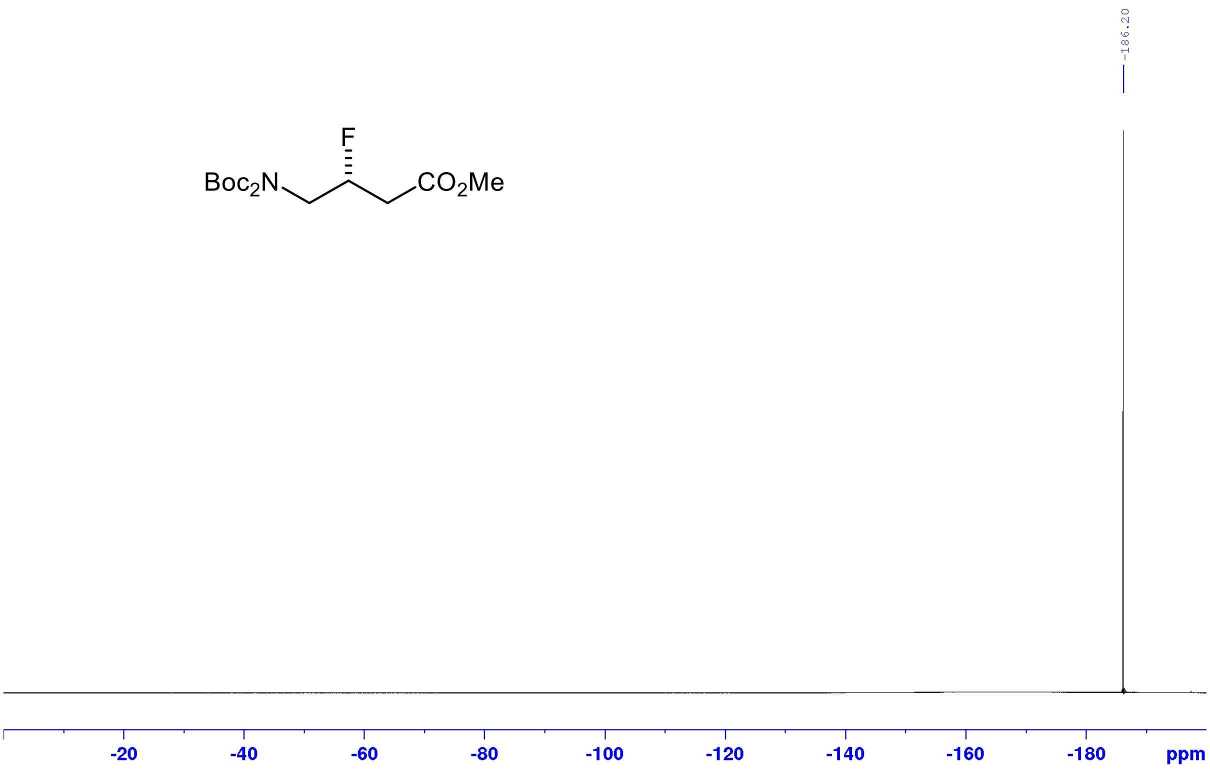

^{1}H NMR (400 MHz, $CDCl_3$) of **25**

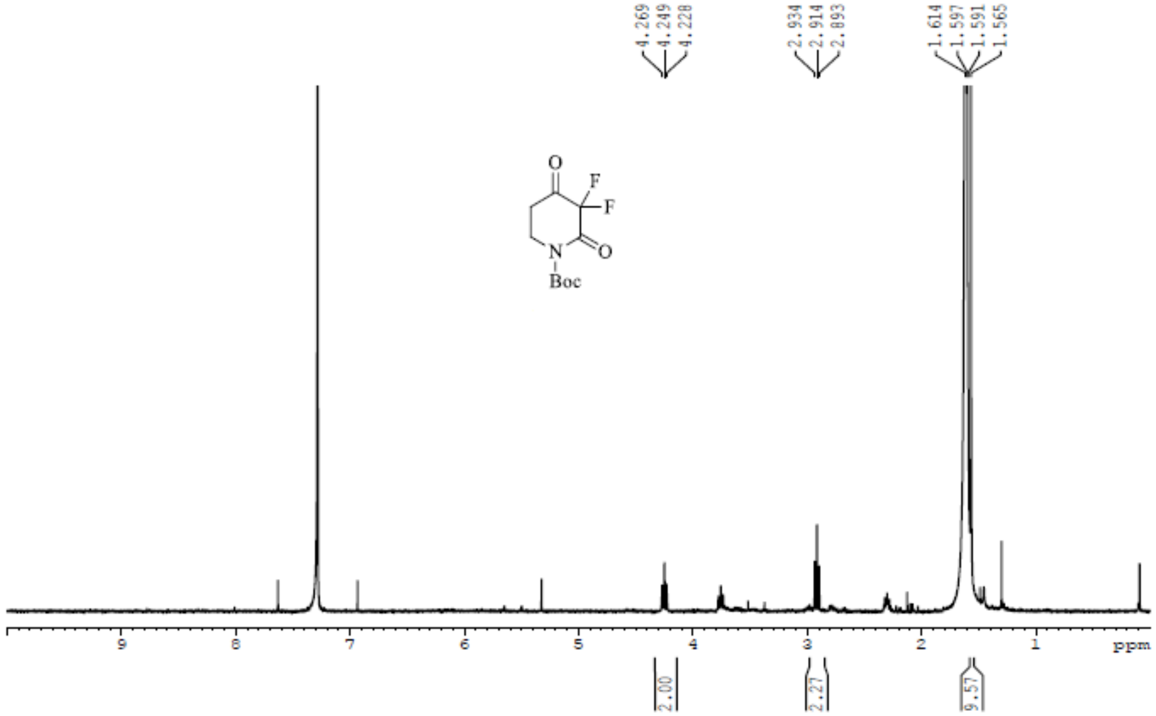

^{19}F NMR (282 MHz, $CDCl_3$) of **25**

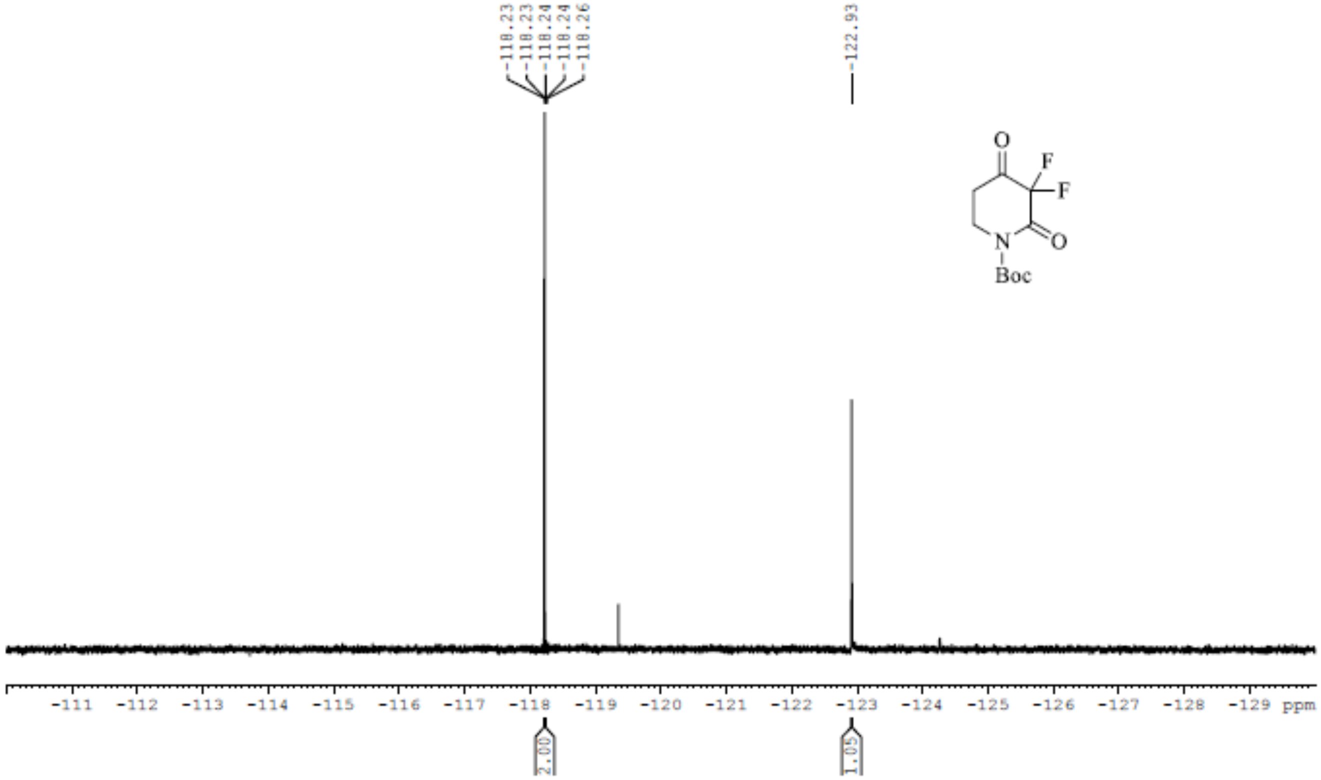

^{1}H NMR (300 MHz, $CDCl_3$) of **26**

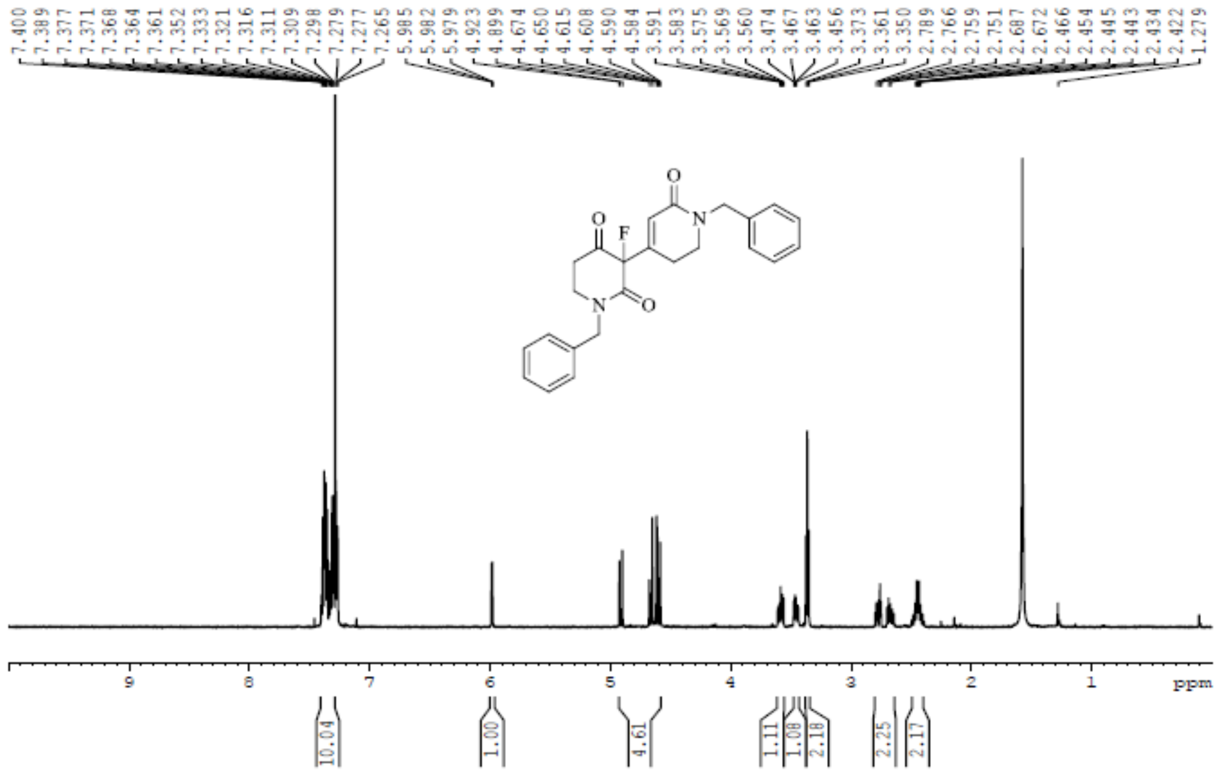

$^{13}C\{^{1}H\}$ NMR (75 MHz, $CDCl_3$) of **26**

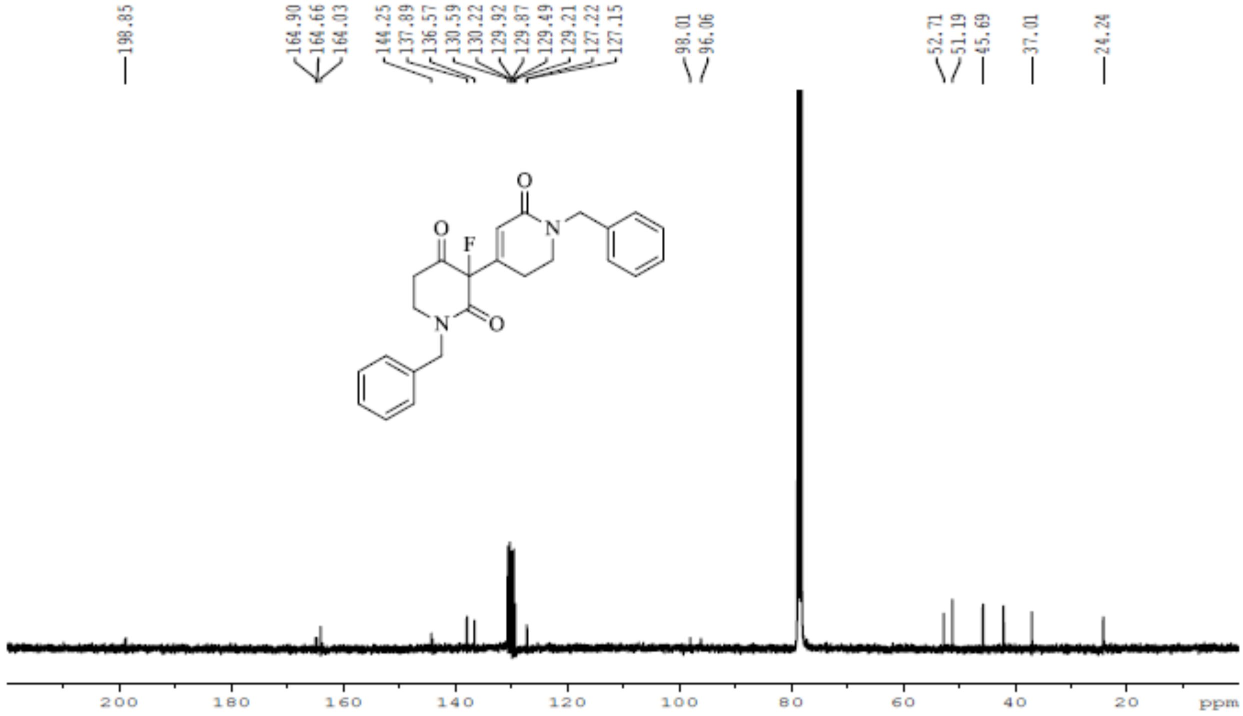

^{19}F NMR (282 MHz, $CDCl_3$) of **26**

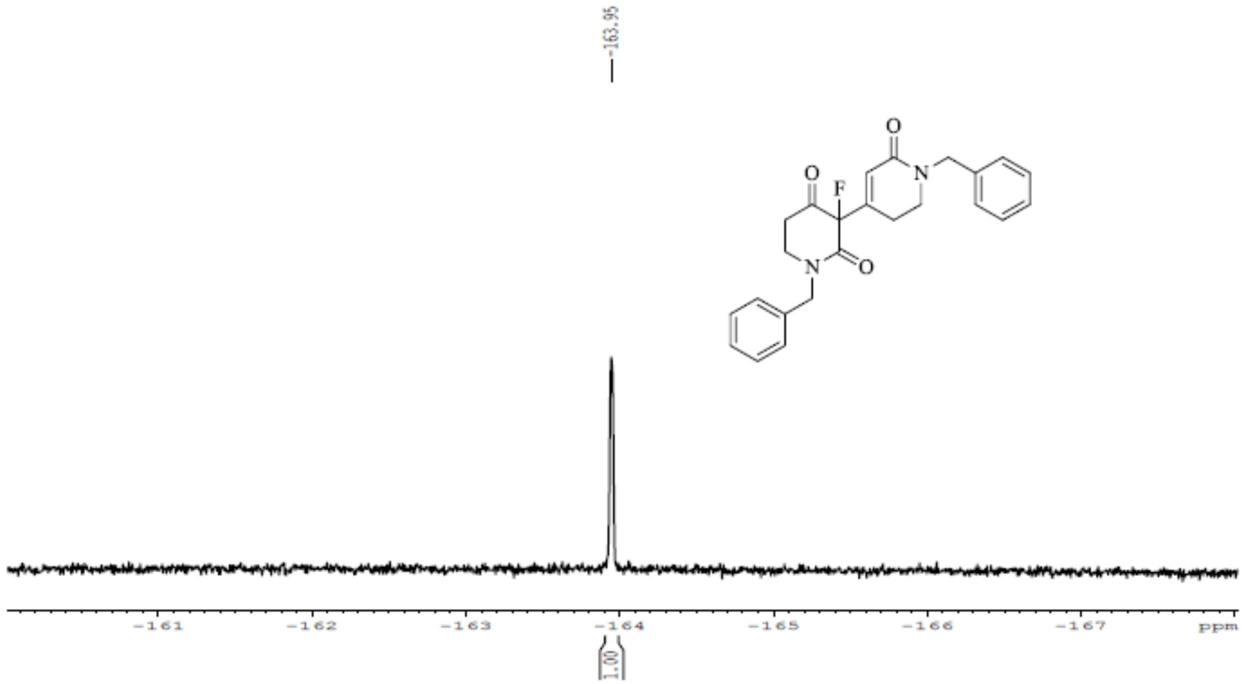

$^{19}F\{^1H\}$ NMR (282 MHz, $CDCl_3$) of **26**

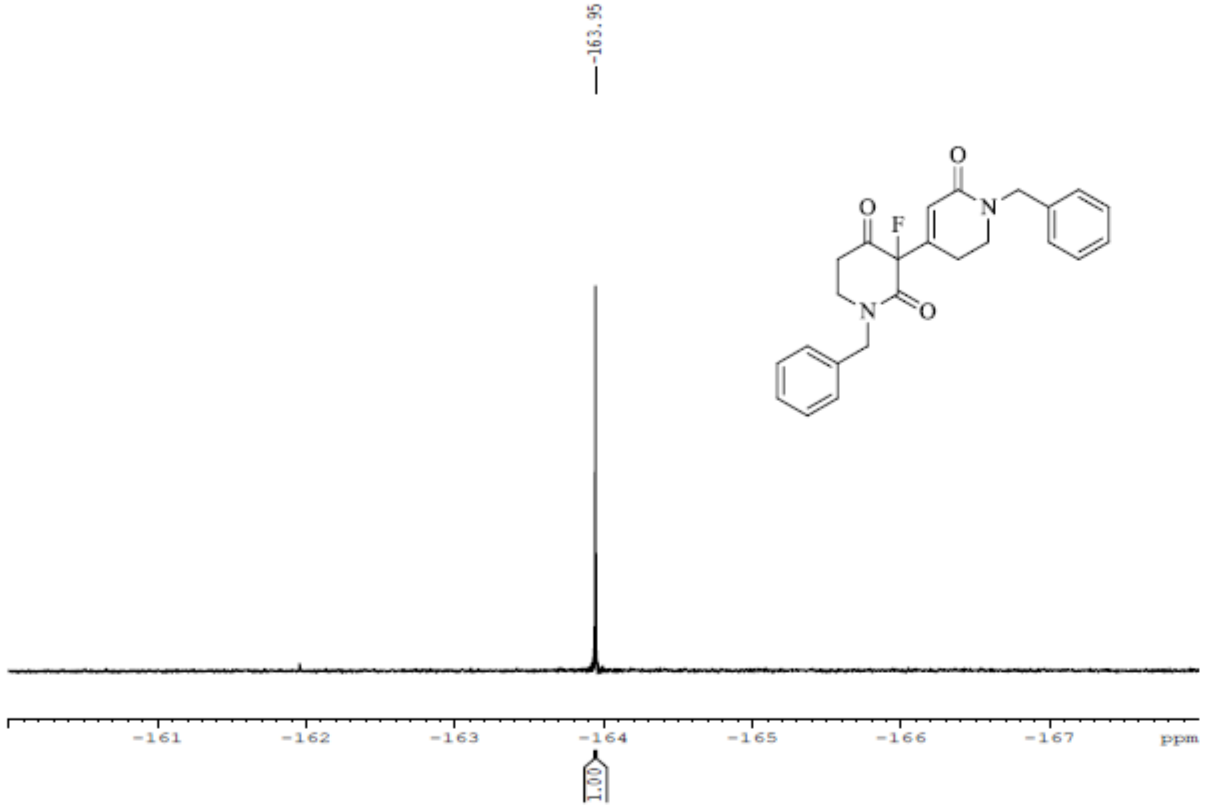

^{1}H NMR (200 MHz, $CDCl_3$) of **28b**

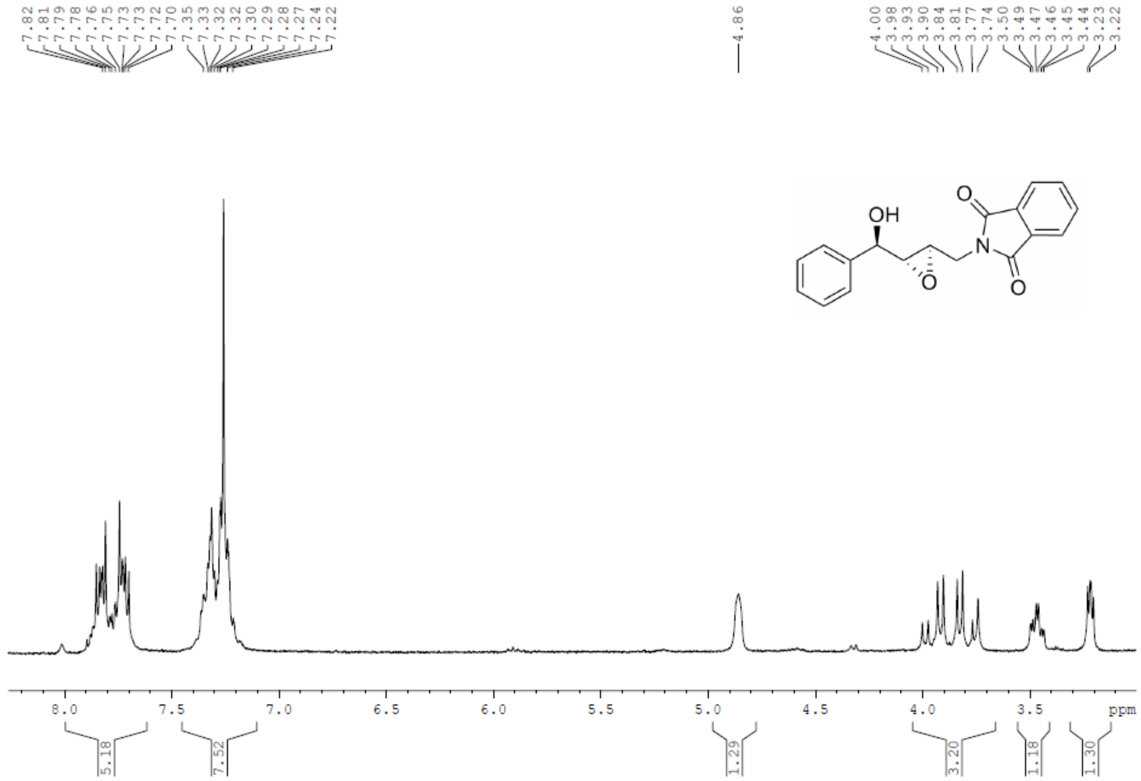

^{1}H NMR (300 MHz, $CDCl_3$) of **30**

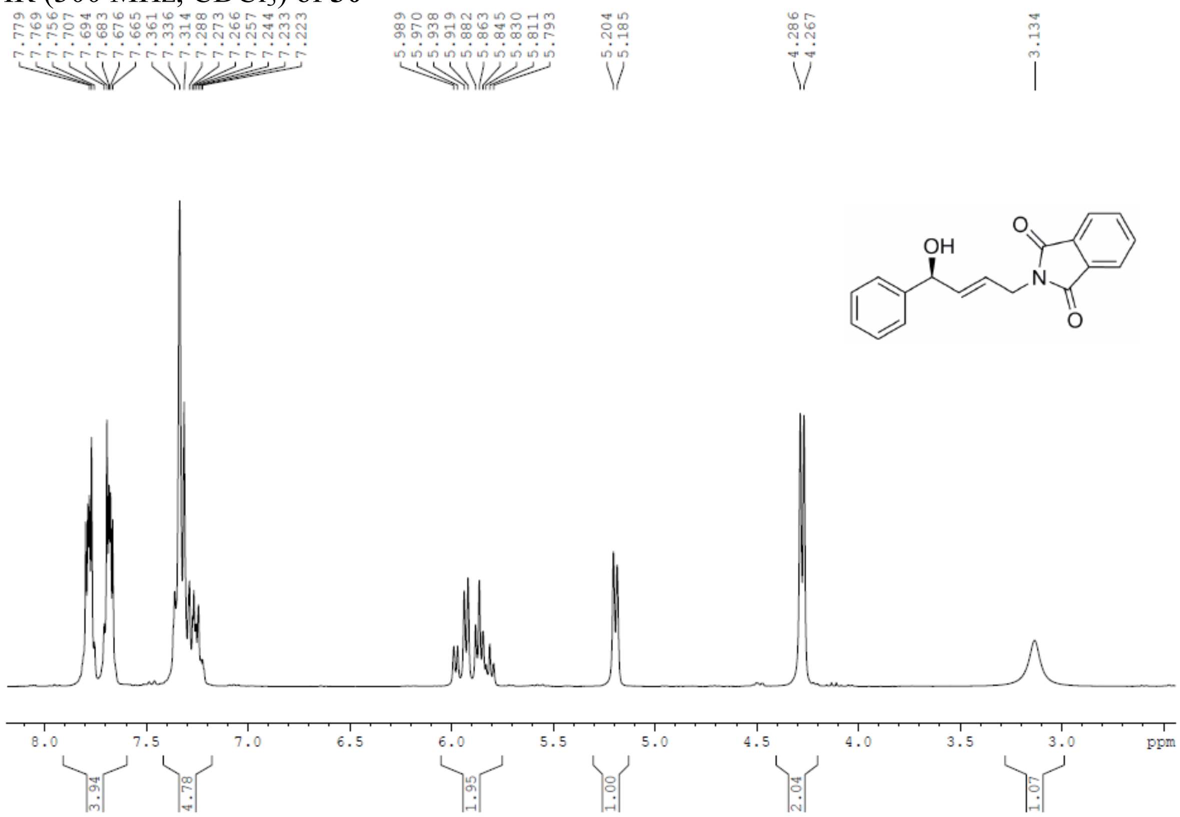

$^{13}C\{^{1}H\}$ NMR (75 MHz, $CDCl_3$) of **30**

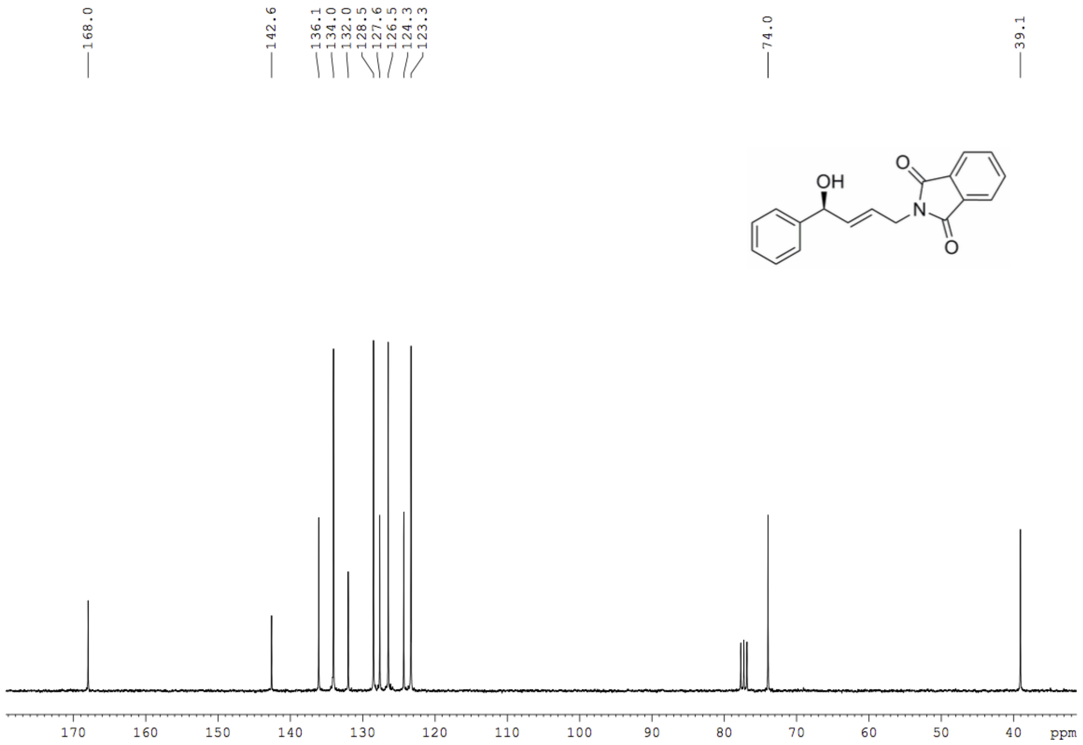

^{1}H NMR (300 MHz, $CDCl_3$) of **31**

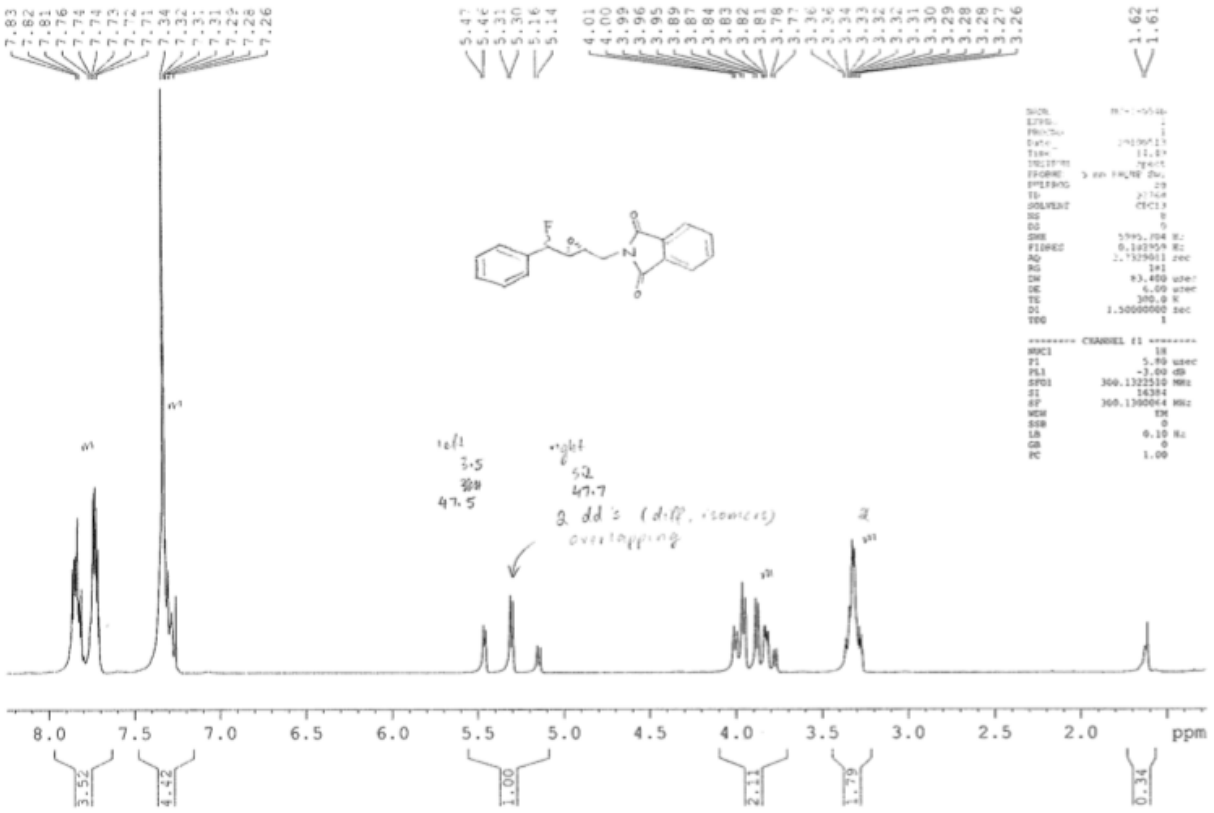

$^{19}F\{^{1}H\}$ NMR (282 MHz, $CDCl_3$) of **31**

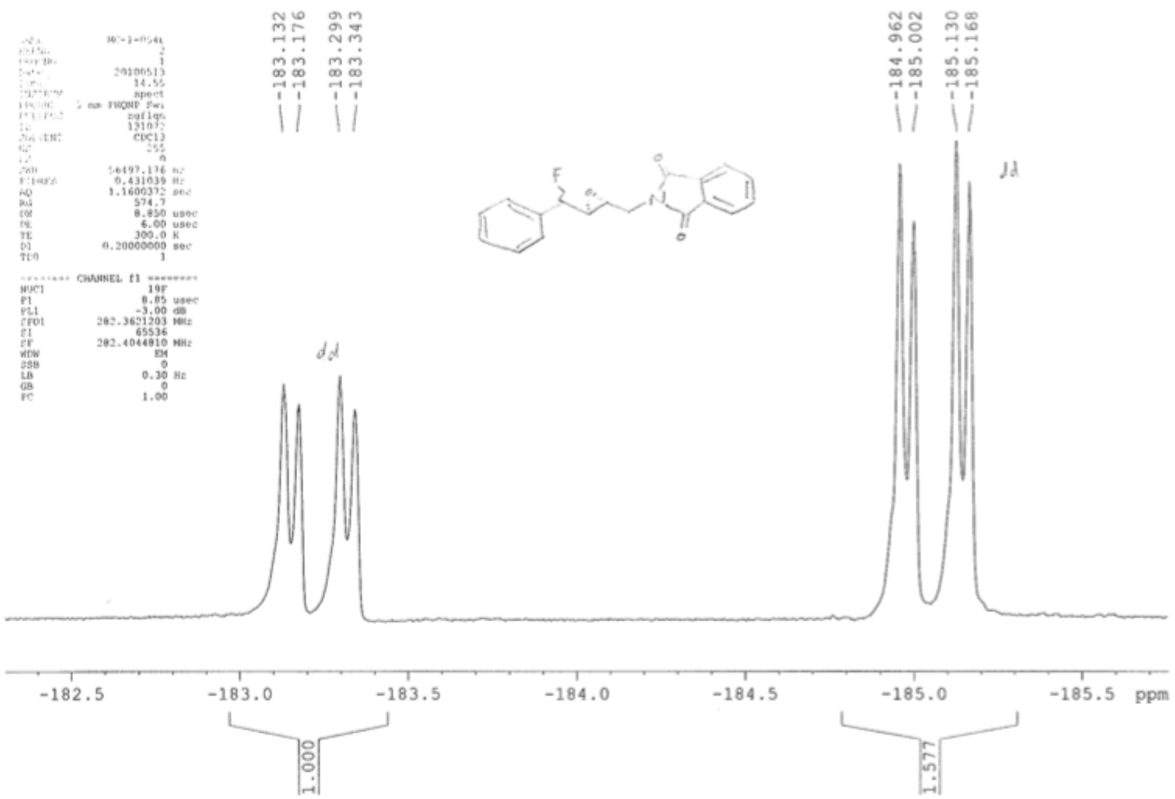

^{1}H NMR (300 MHz, $CDCl_3$) of **32 + 33**

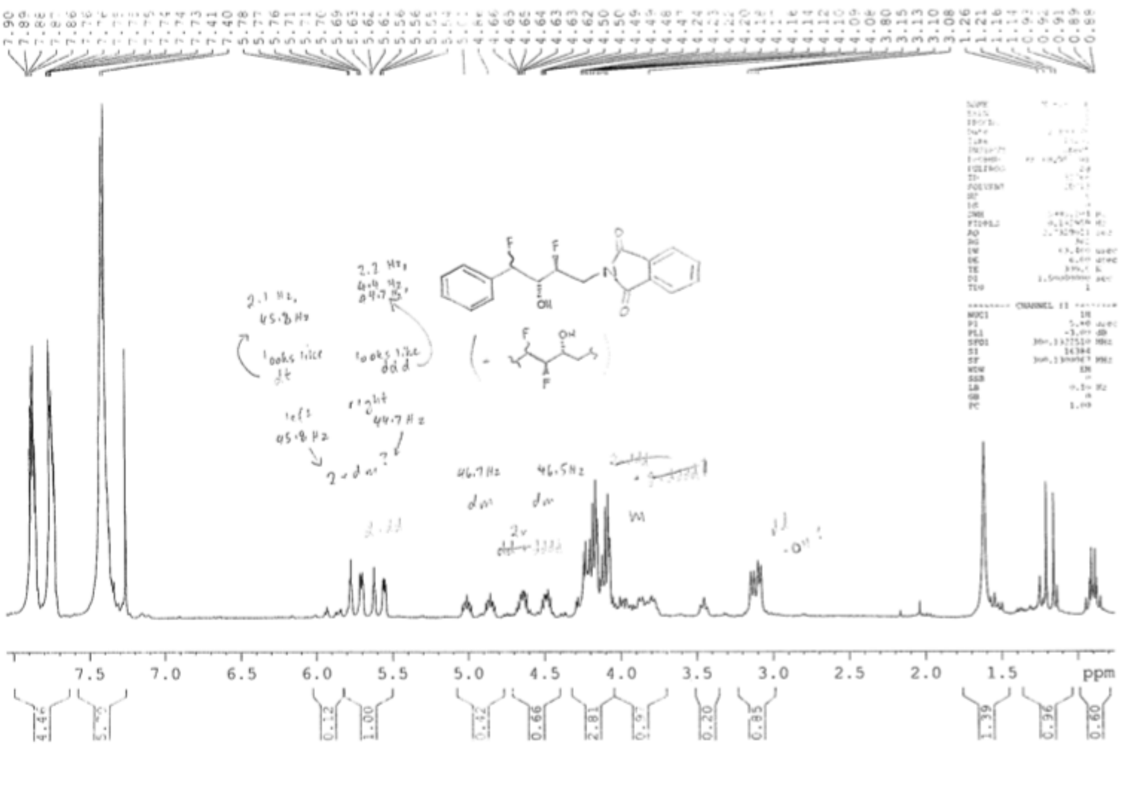

^{19}F NMR (282 MHz, $CDCl_3$) of **32 + 33**

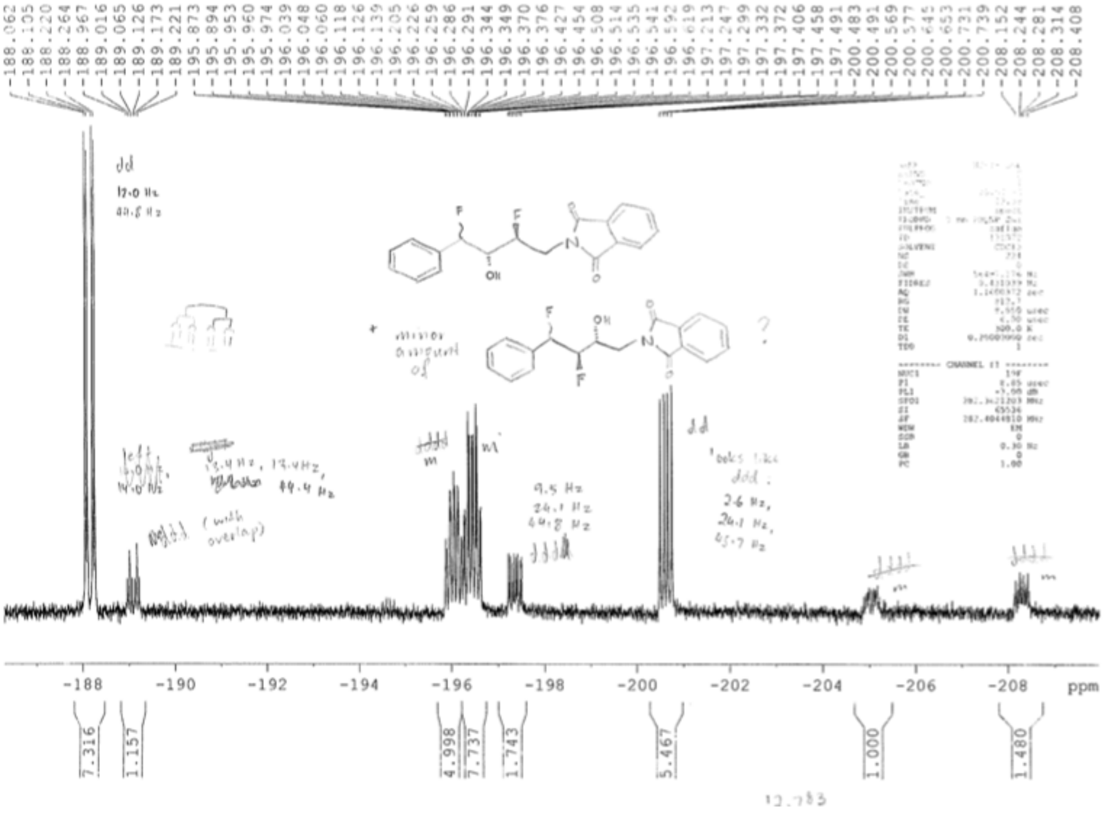

$^{19}F\{^1H\}$ NMR (282 MHz, $CDCl_3$) of **32 + 33**

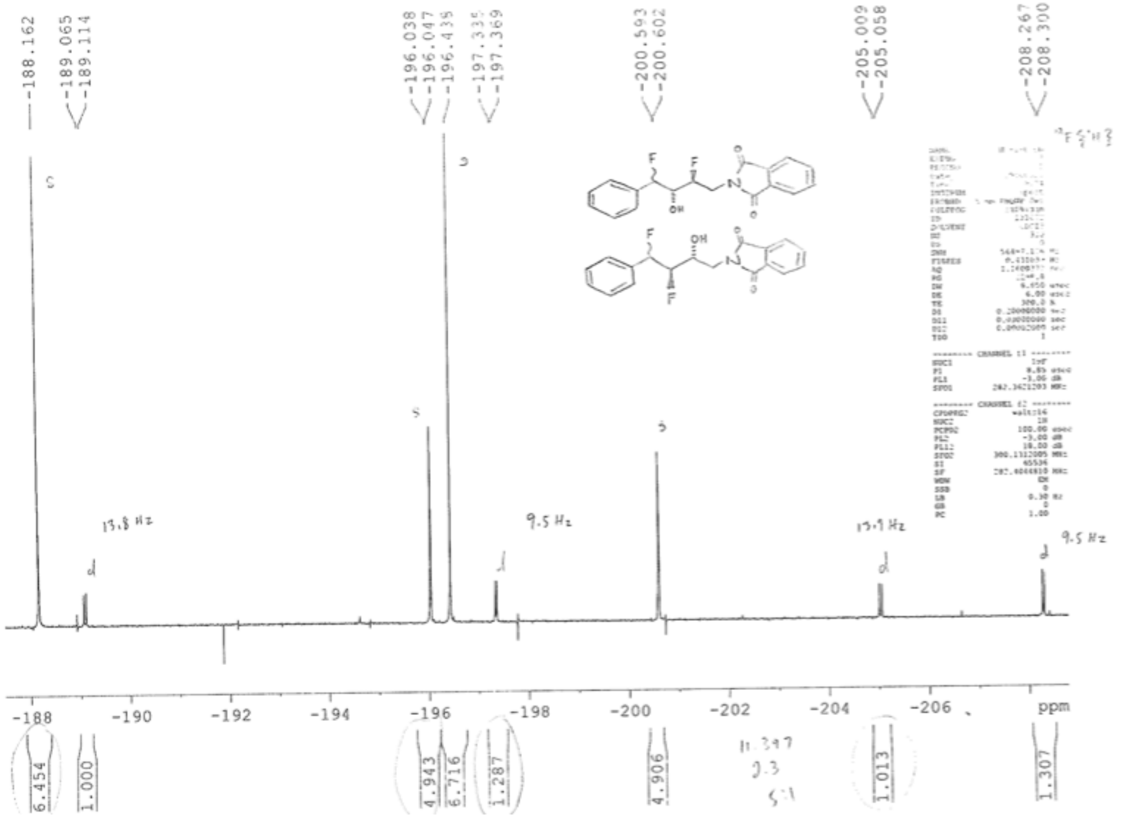

^{1}H NMR (300 MHz, $CDCl_3$) of **41**

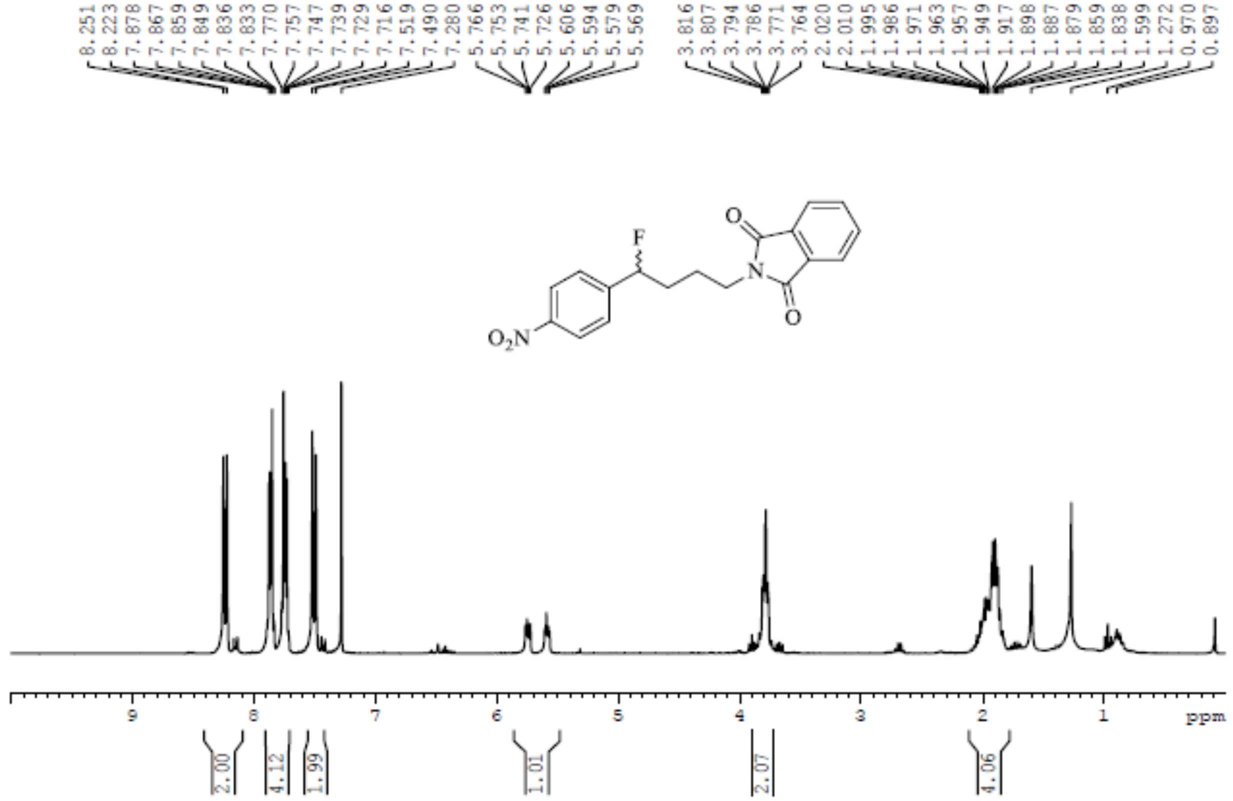

$^{13}C\{^{1}H\}$ NMR (75 MHz, $CDCl_3$) of **41**

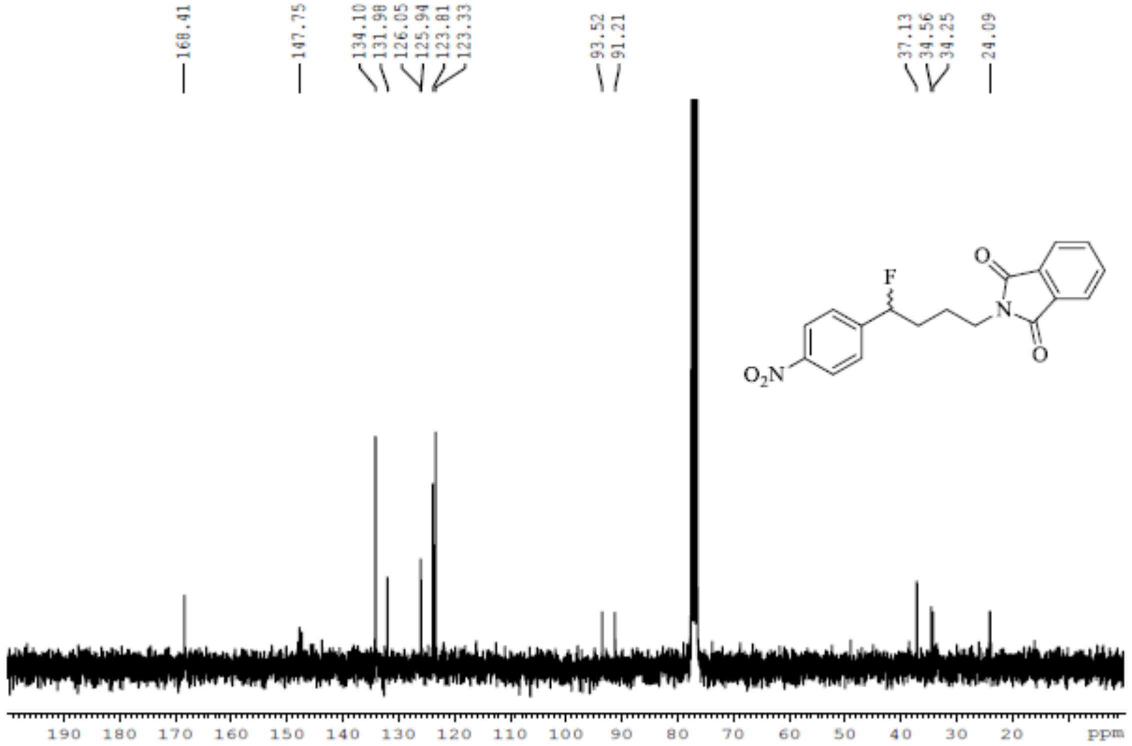

^{19}F NMR (282 MHz, $CDCl_3$) of **41**

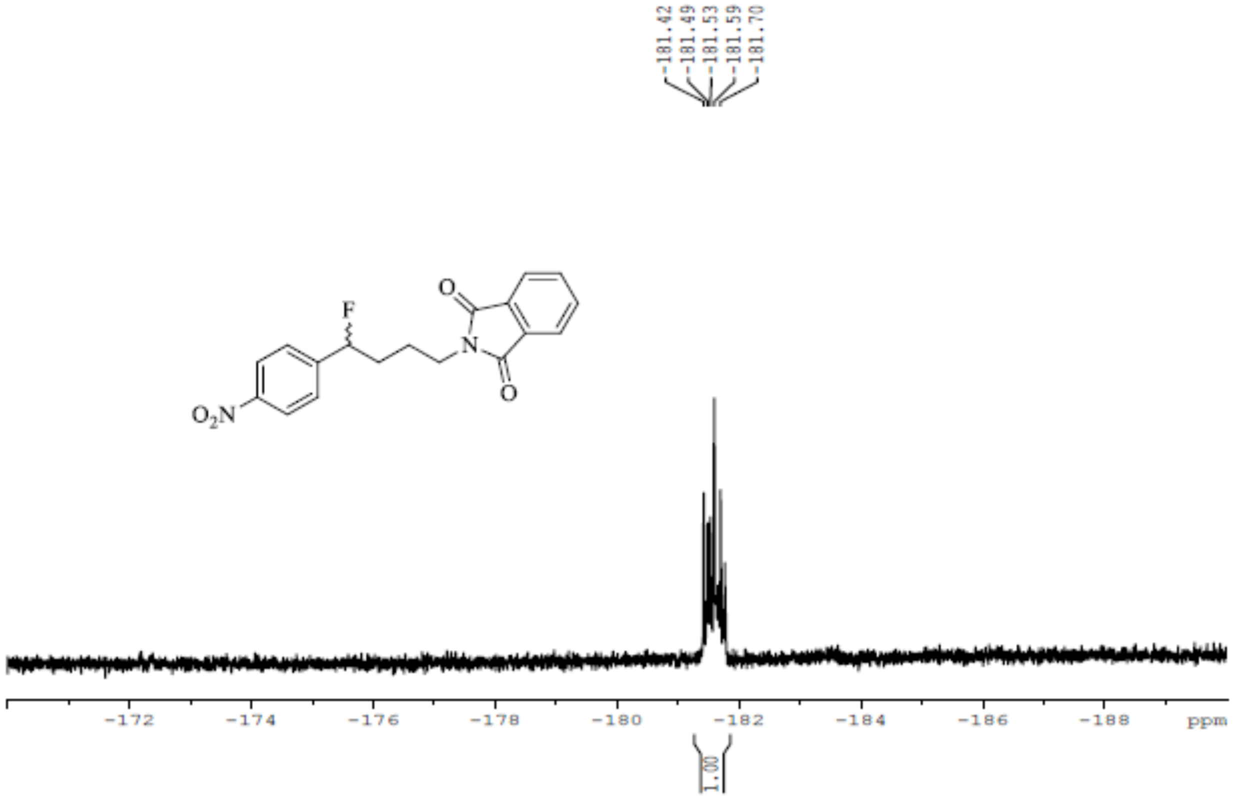

^{19}F{^{1}H} NMR (282 MHz, $CDCl_3$) of **41**

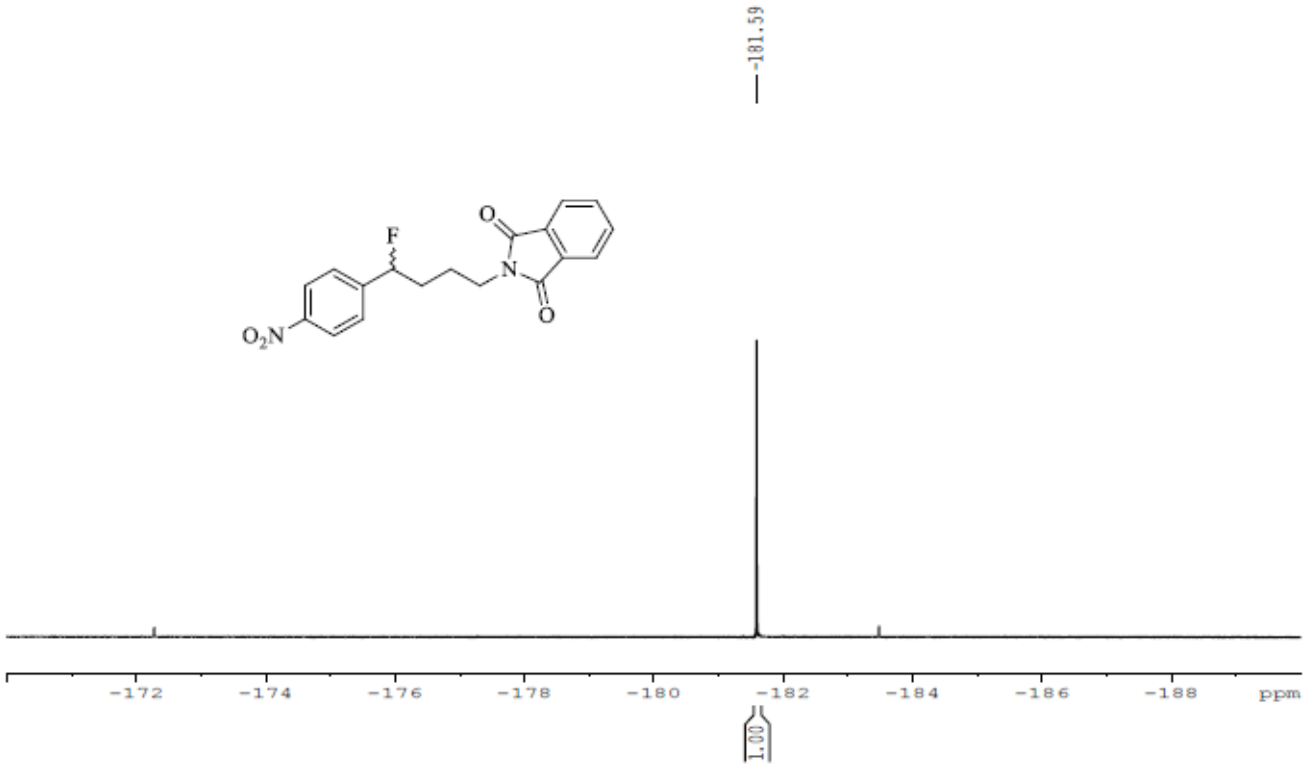

^{1}H NMR (300 MHz, $CDCl_3$) of **43**

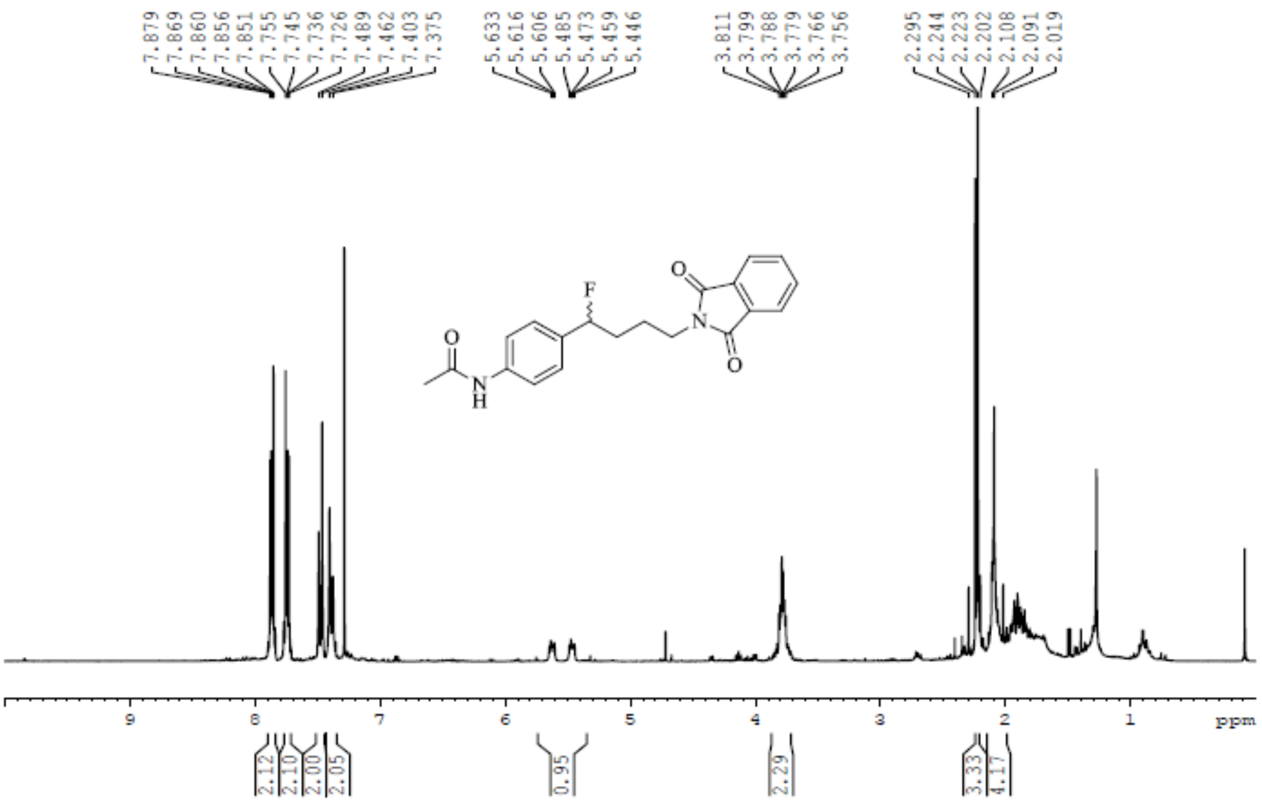

^{13}C{^{1}H} NMR (75 MHz, $CDCl_3$) of **43**

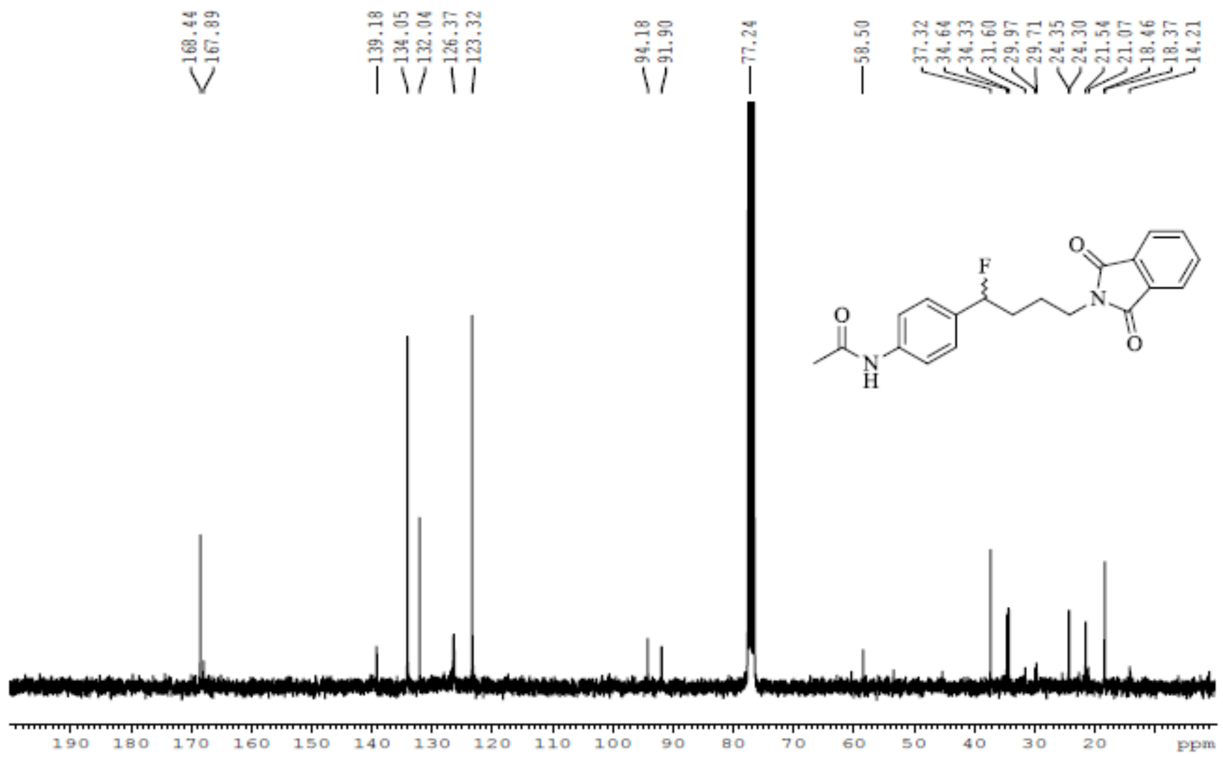

^{19}F NMR (282 MHz, $CDCl_3$) of **43**

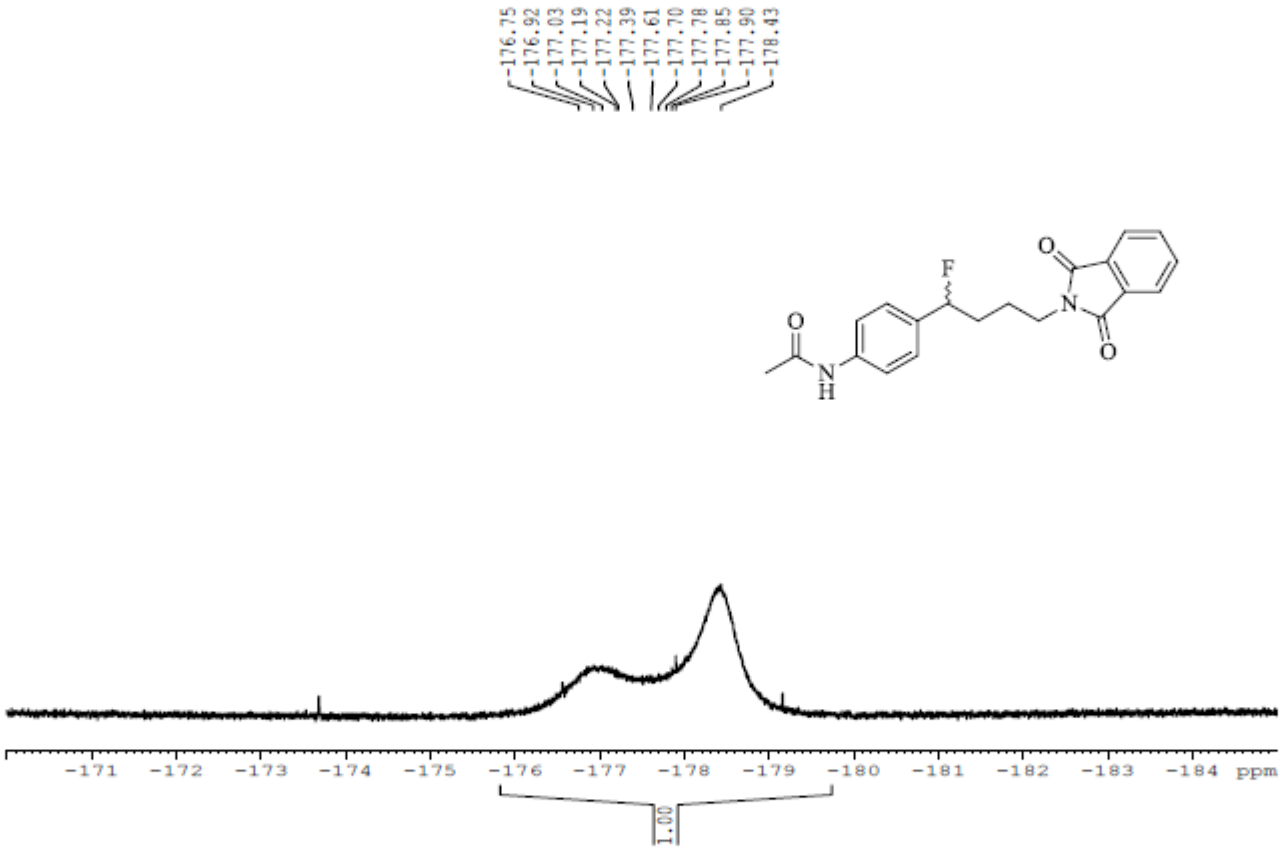

$^{19}F\{^{1}H\}$ NMR (282 MHz, $CDCl_3$) of **43**

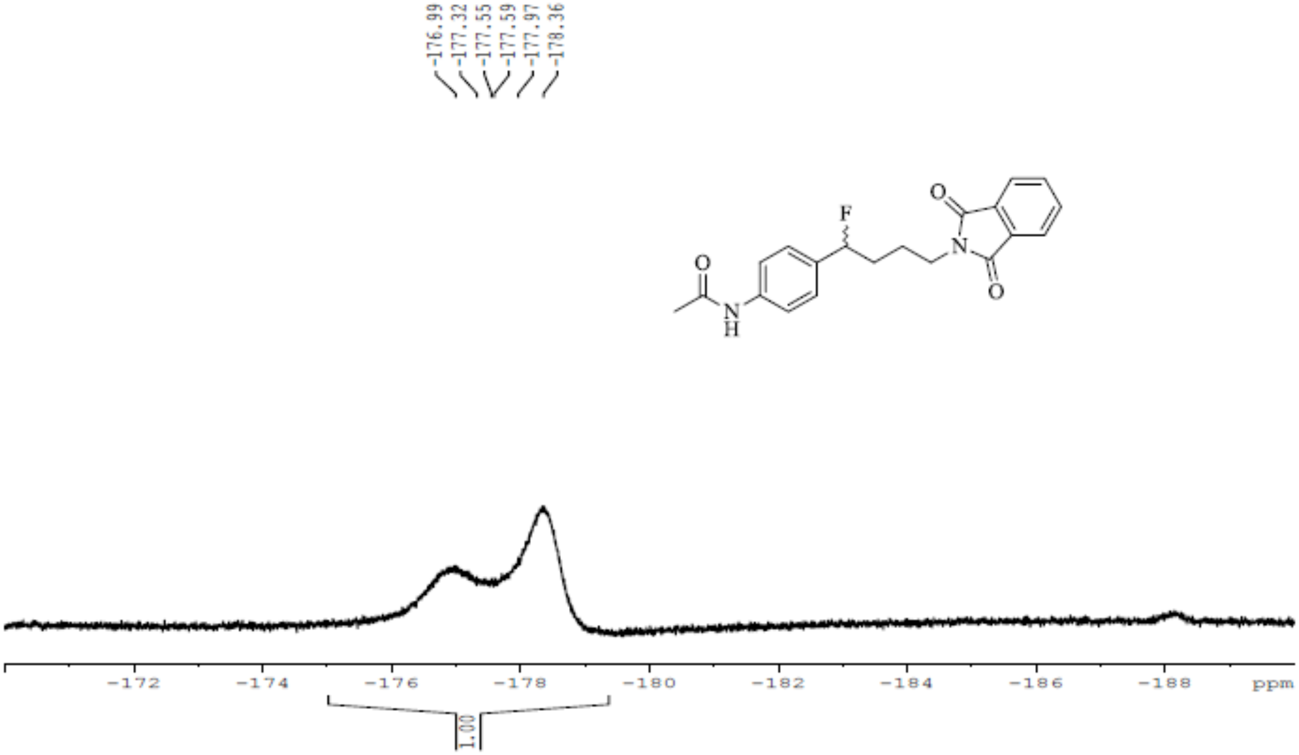

^{1}H NMR (300 MHz, $CDCl_3$) of **44**

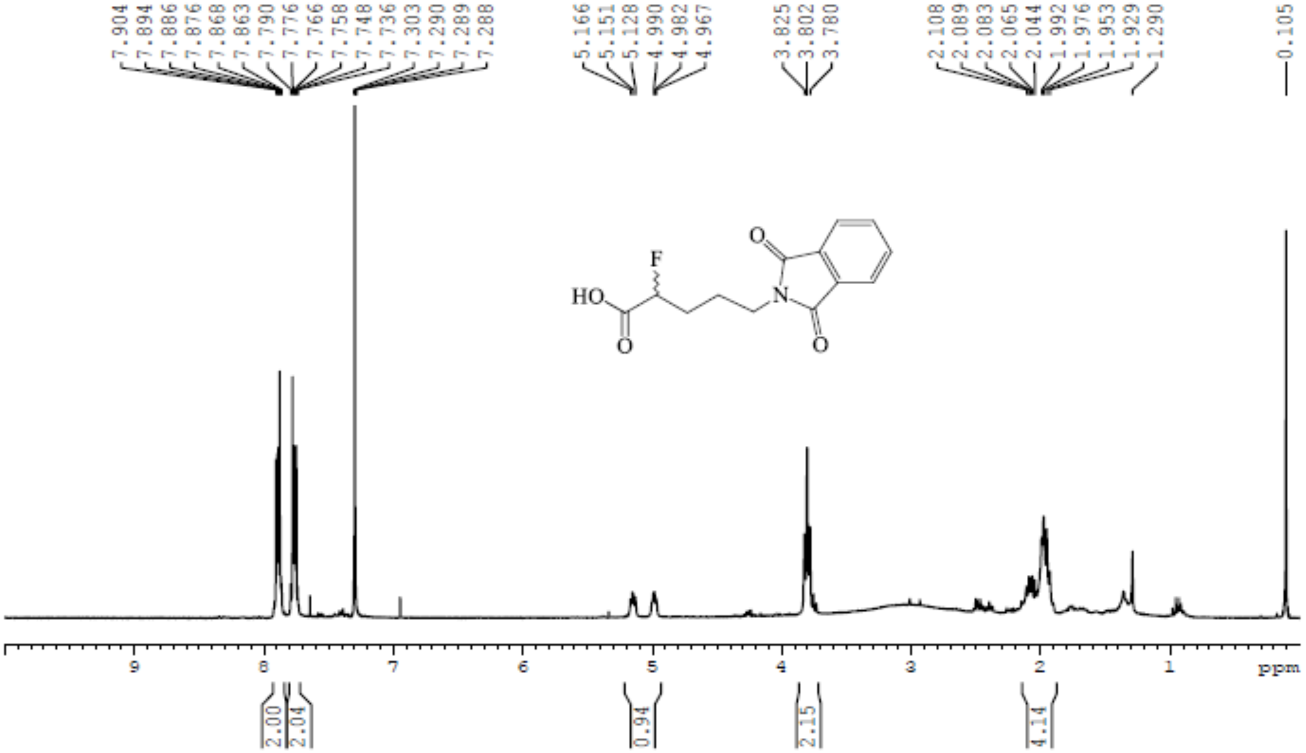

$^{13}C\{^{1}H\}$ NMR (75 MHz, $CDCl_3$) of **44**

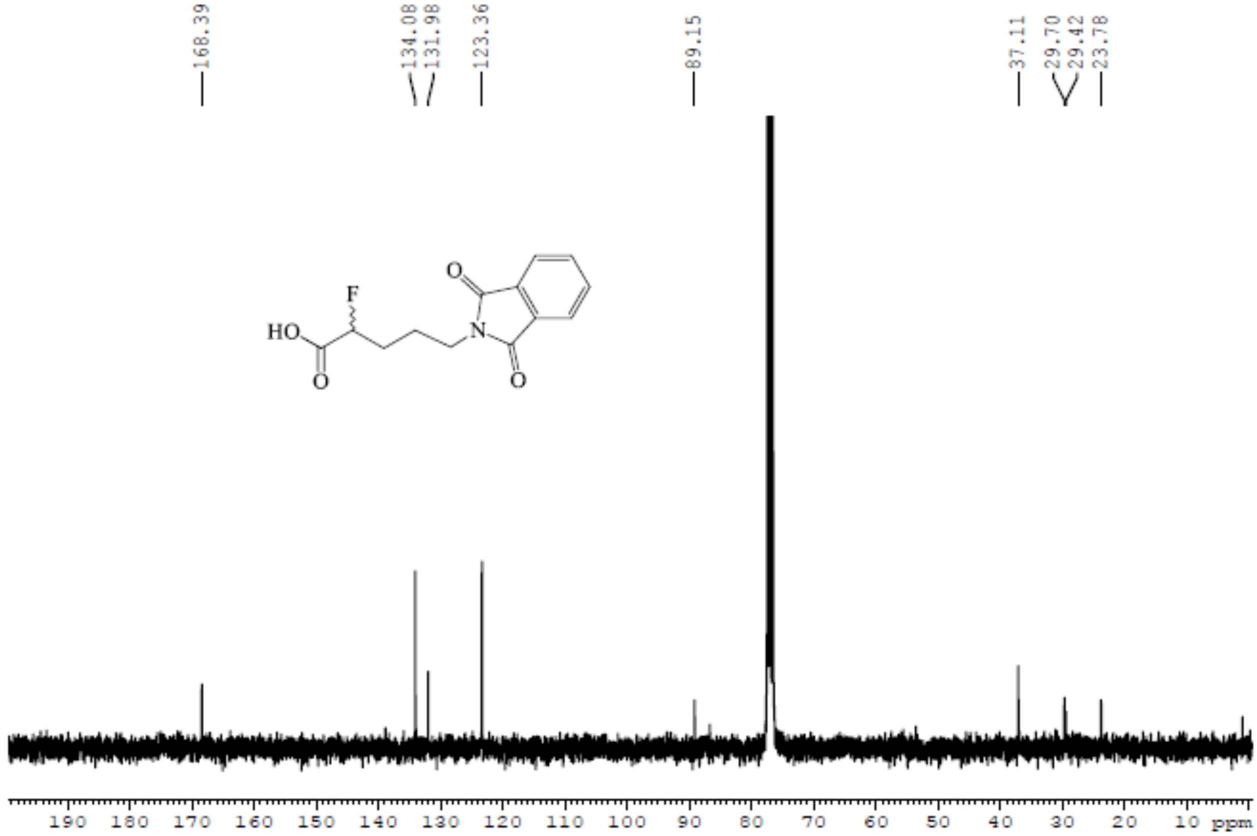

^{19}F NMR (282 MHz, $CDCl_3$) of **44**

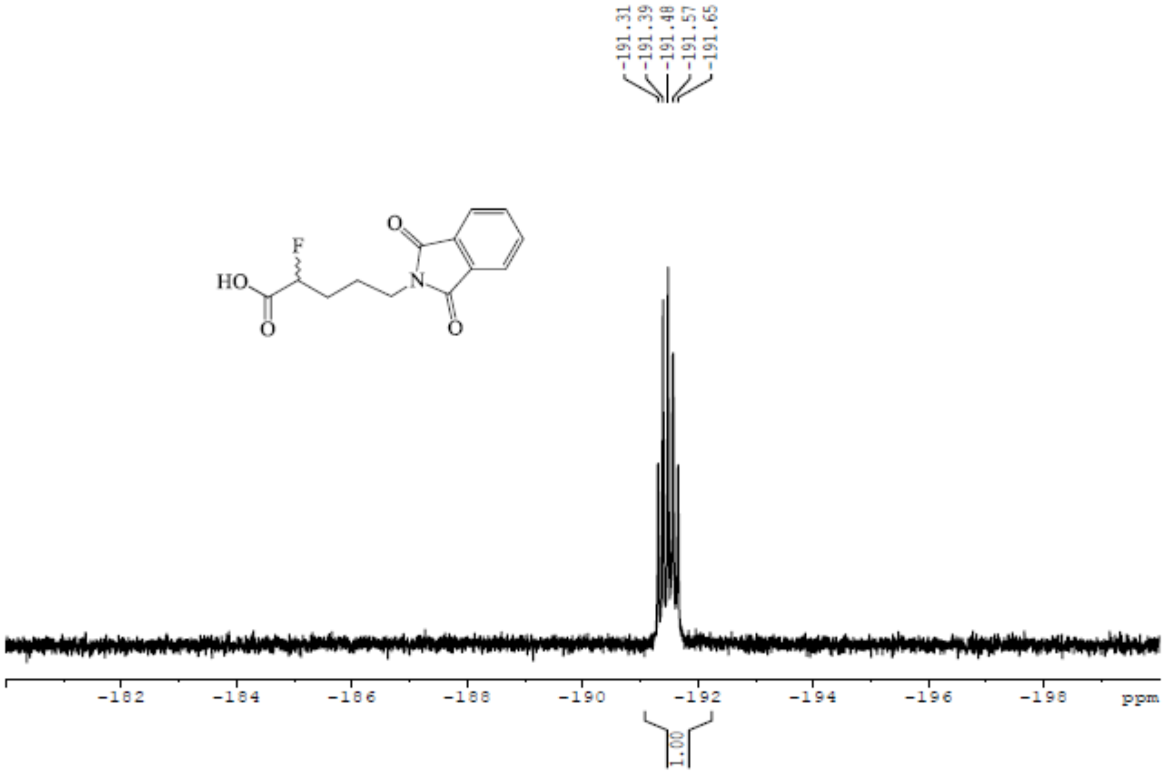

$^{19}F\{^1H\}$ NMR (282 MHz, $CDCl_3$) of **44**

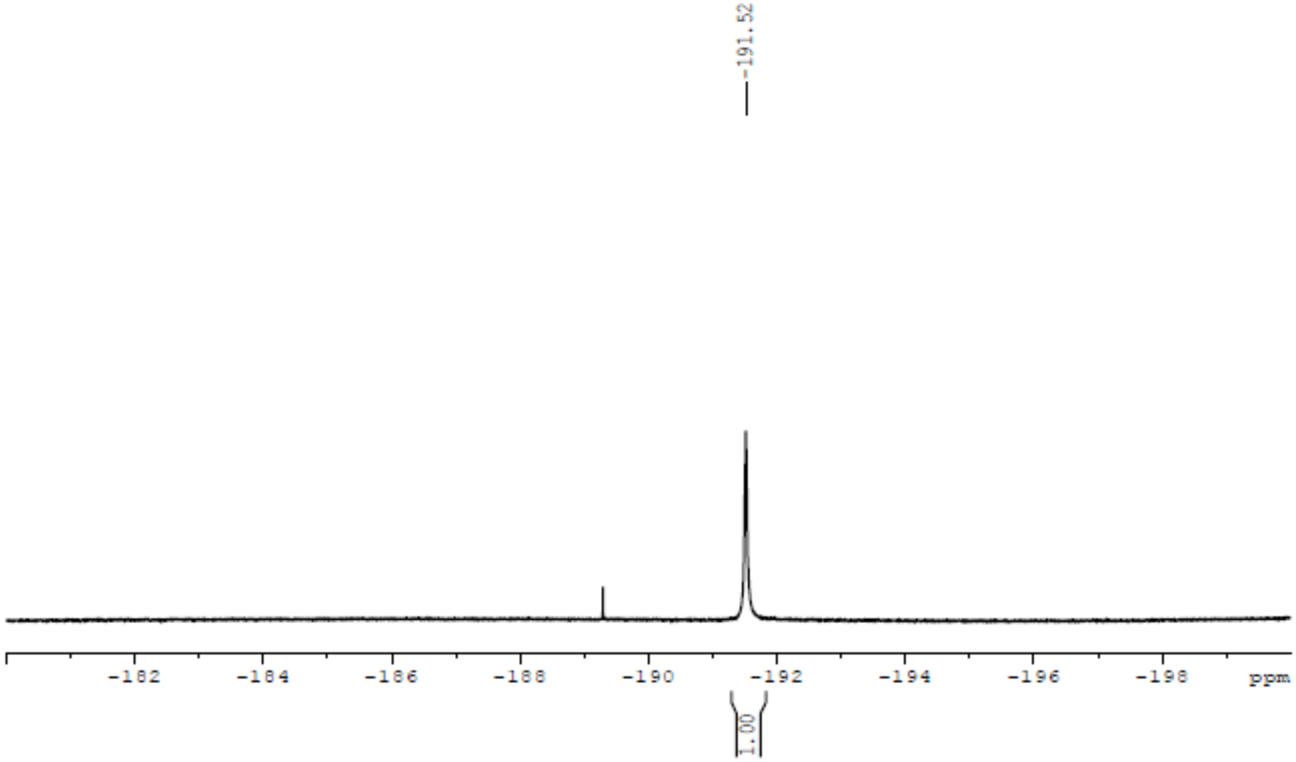

^{1}H NMR (300 MHz, $CDCl_3$) of **48**

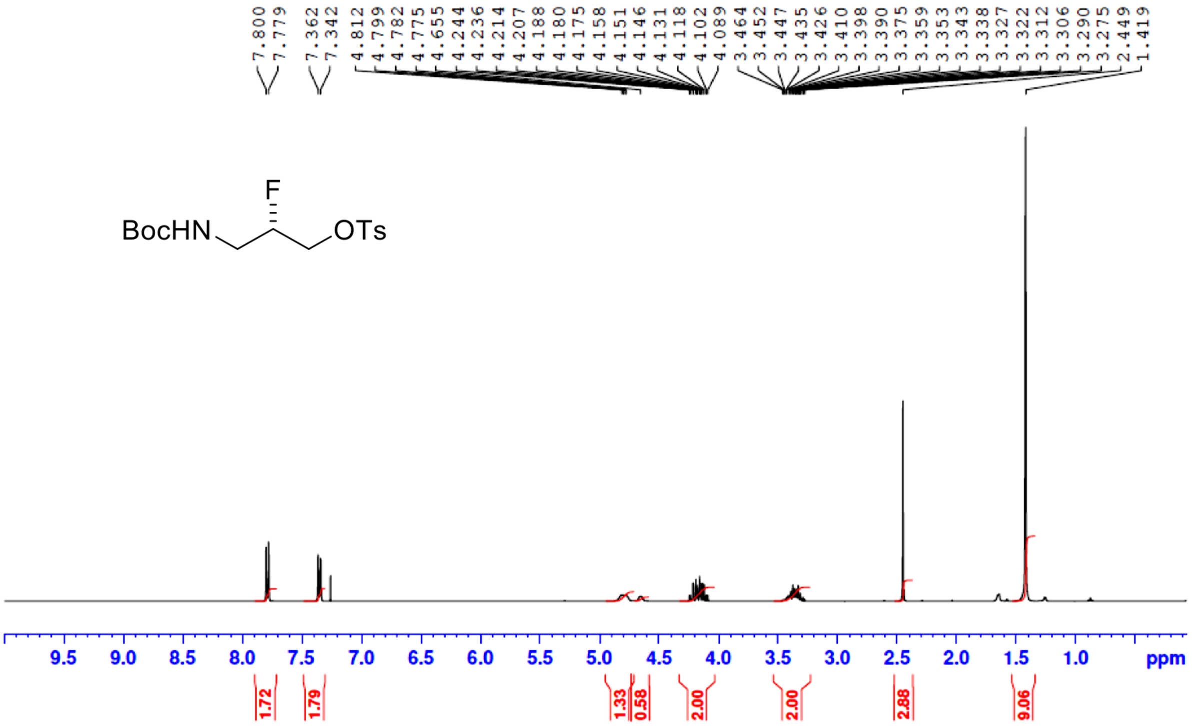

^{13}C NMR (75 MHz, $CDCl_3$) of **48**

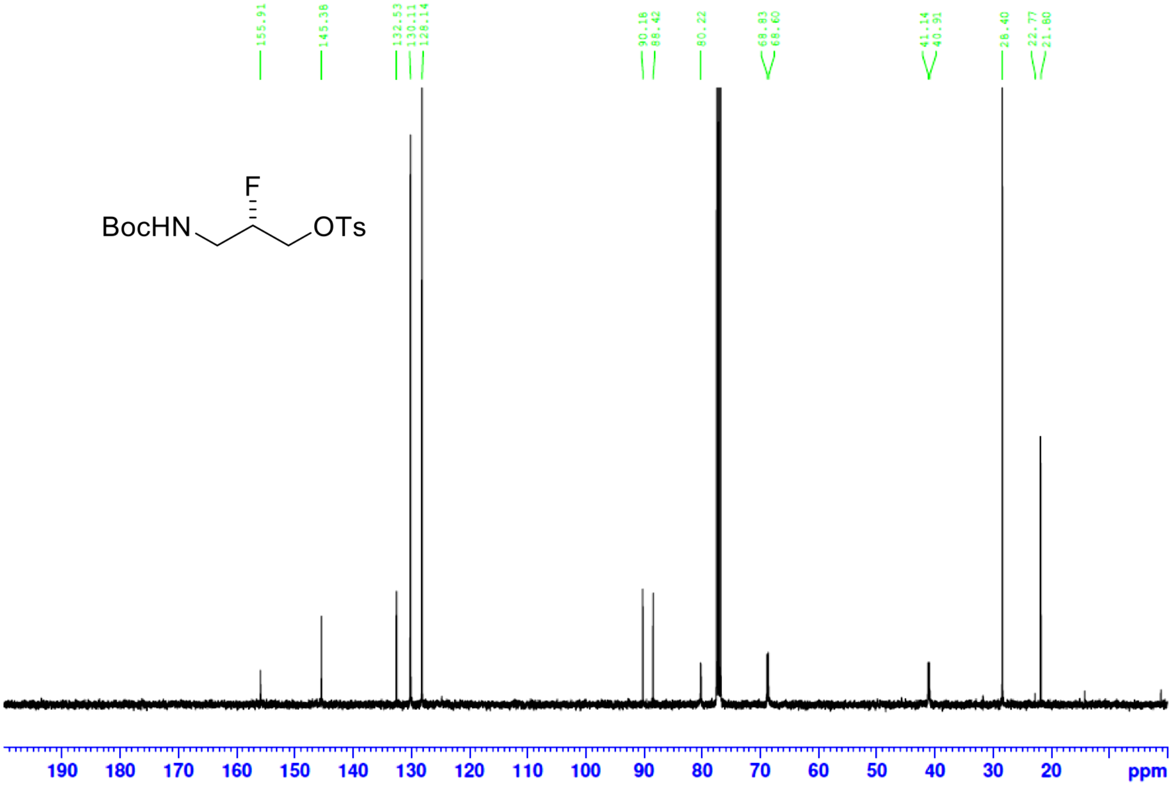

^{19}F NMR (282 MHz, $CDCl_3$) of **48**

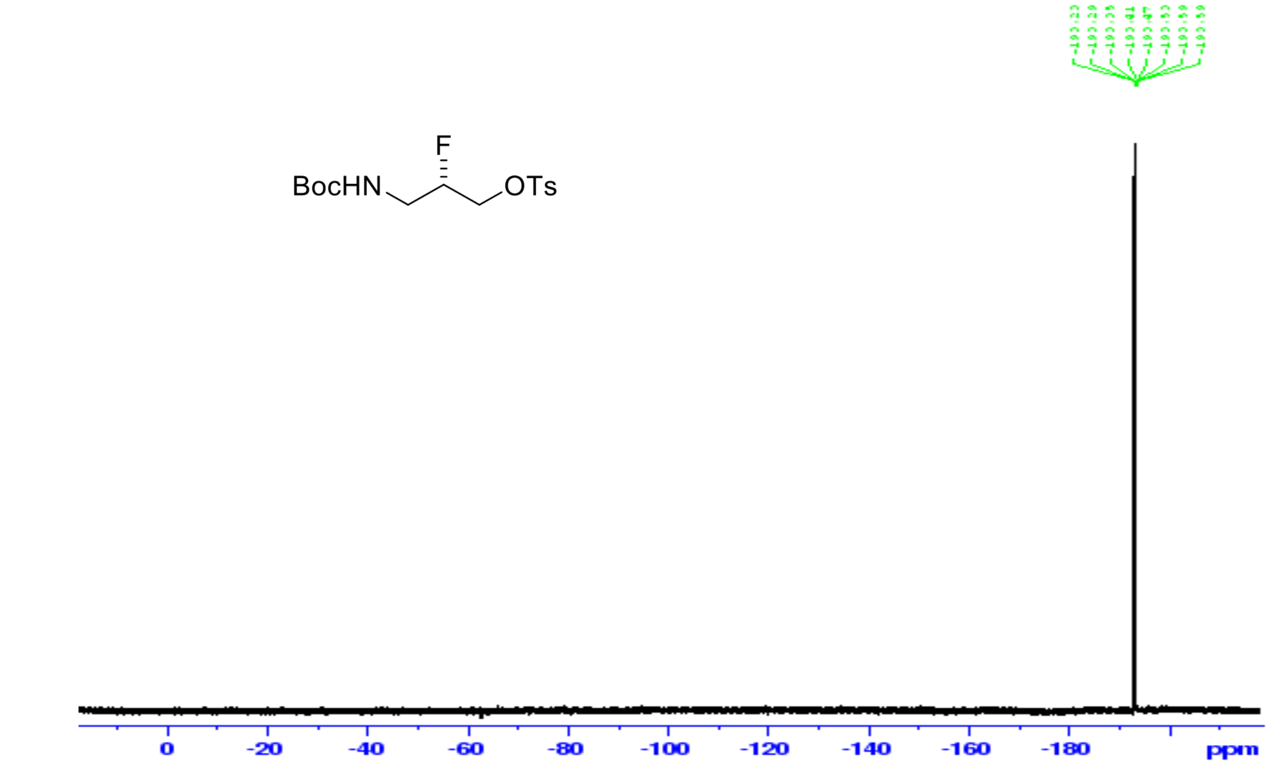

^{1}H NMR (300 MHz, $CDCl_3$) of **50**

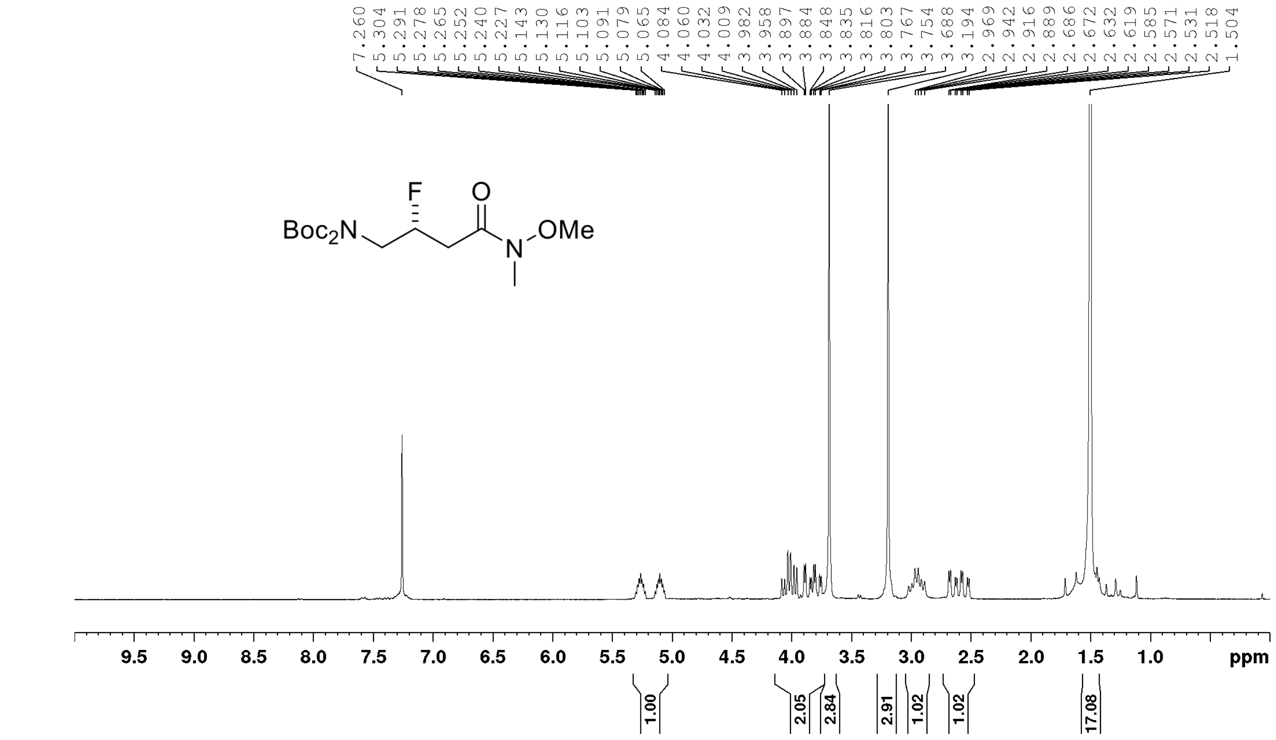

$^{13}C\{^{1}H\}$ NMR (75 MHz, $CDCl_3$) of **50**

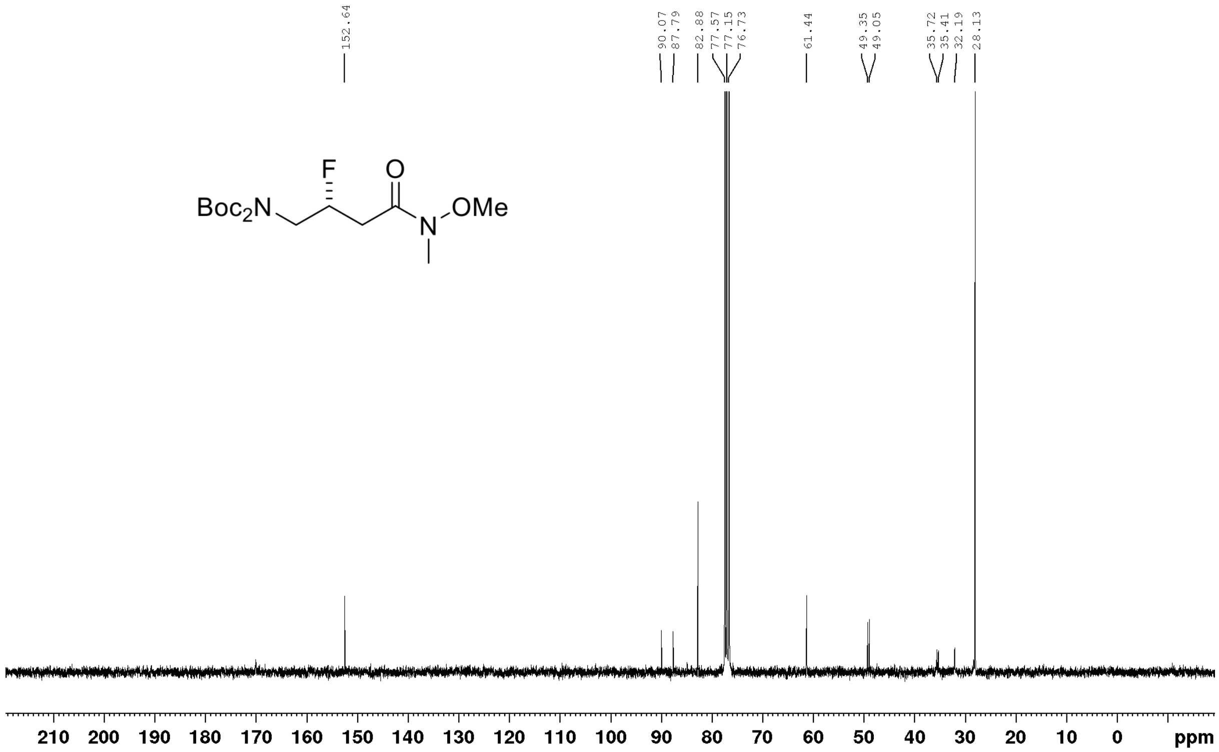

^{19}F NMR (280 MHz, $CDCl_3$) of **50**

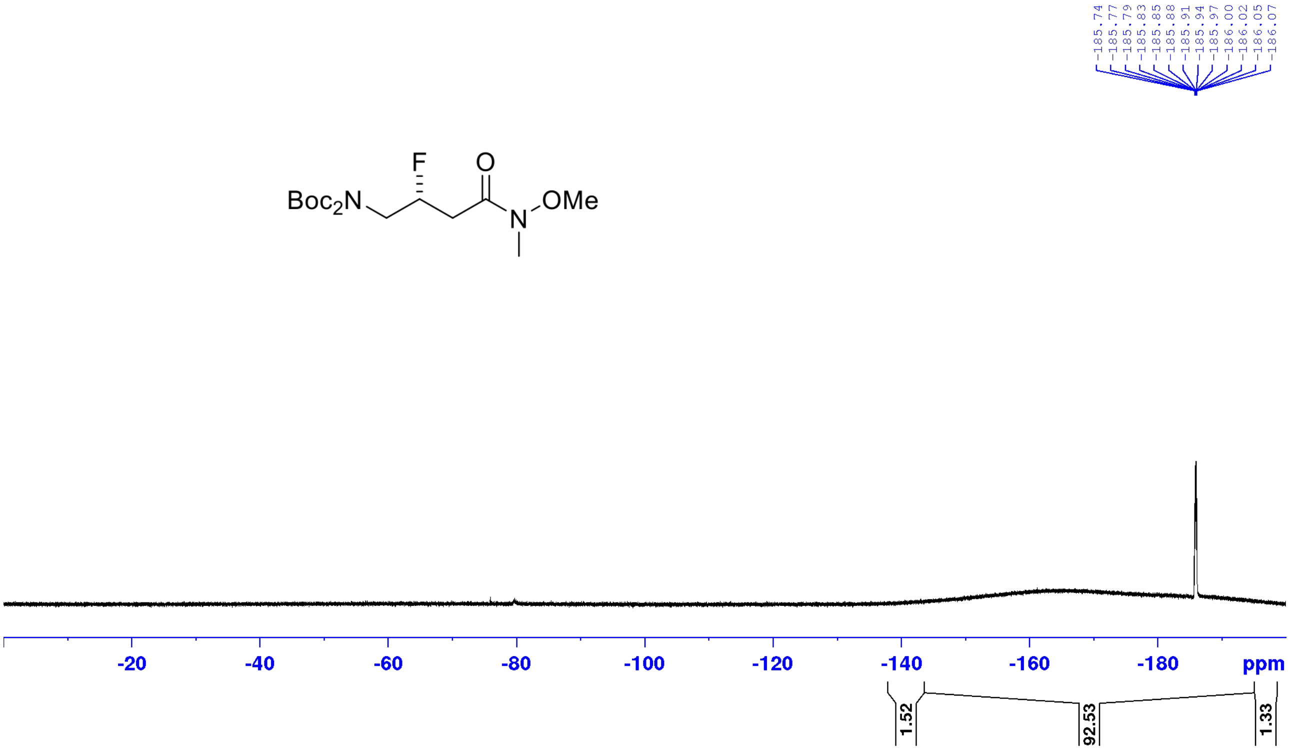

^{1}H NMR (400 MHz, $CDCl_3$) of **53**

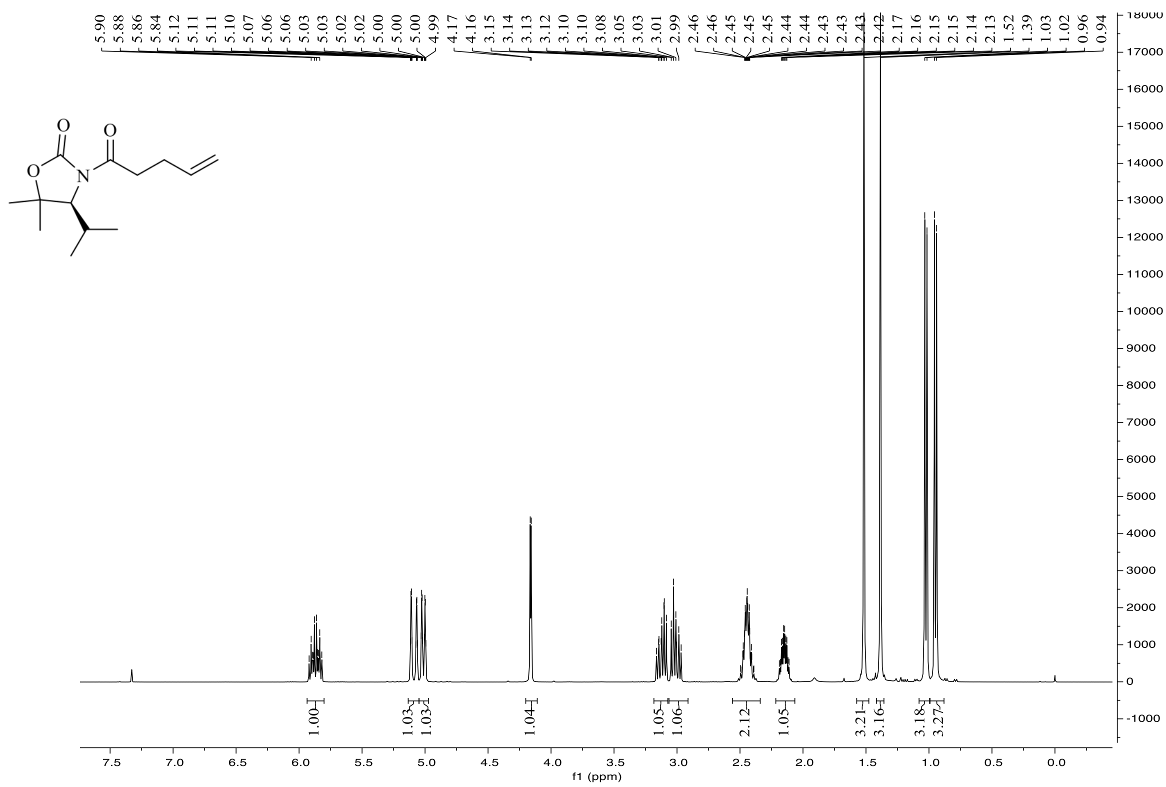

^{13}C{^{1}H} NMR (100 MHz, $CDCl_3$) of **53**

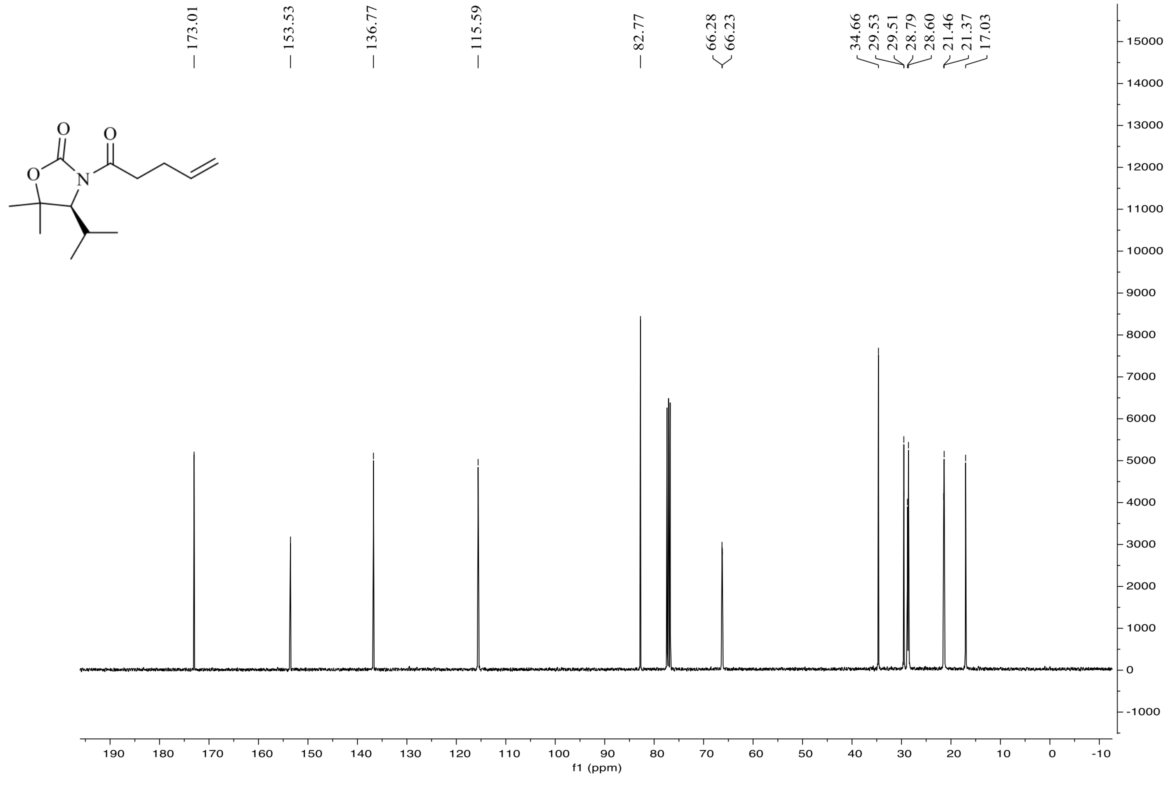

^{1}H NMR (400 MHz, $CDCl_3$) of **54**

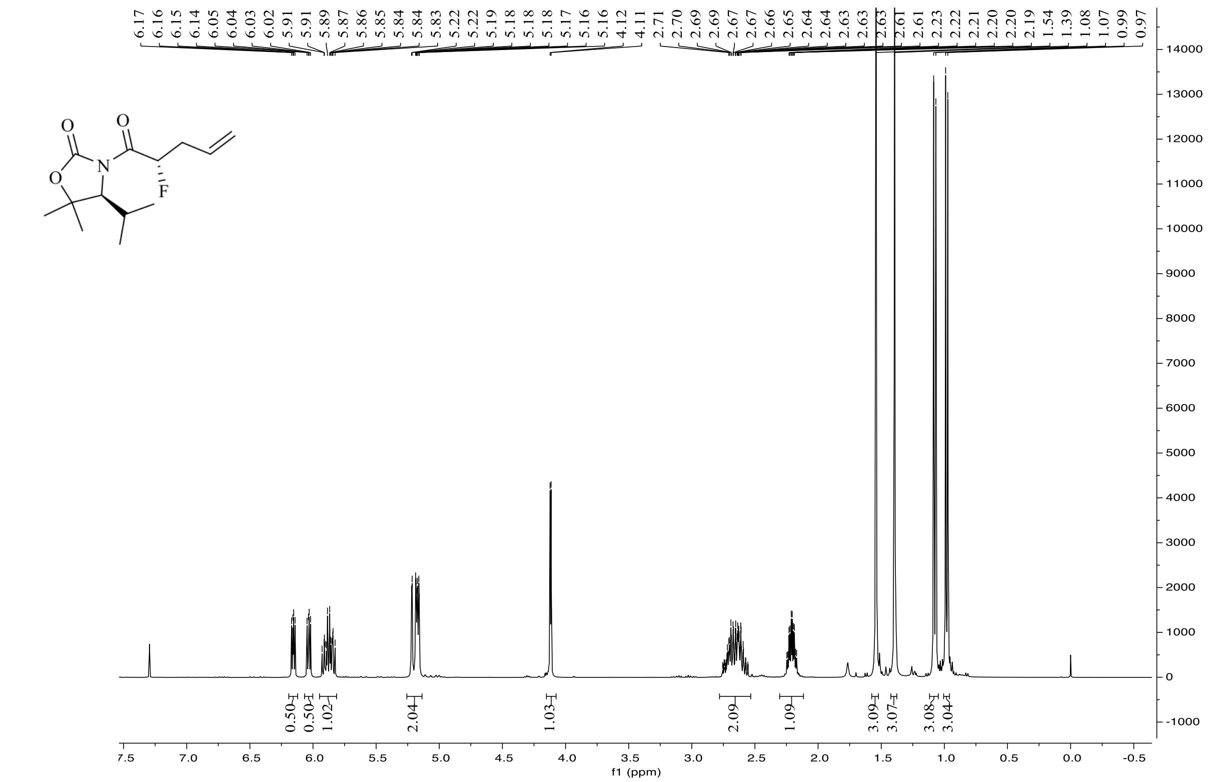

$^{13}C\{^{1}H\}$ NMR (100 MHz, $CDCl_3$) of **54**

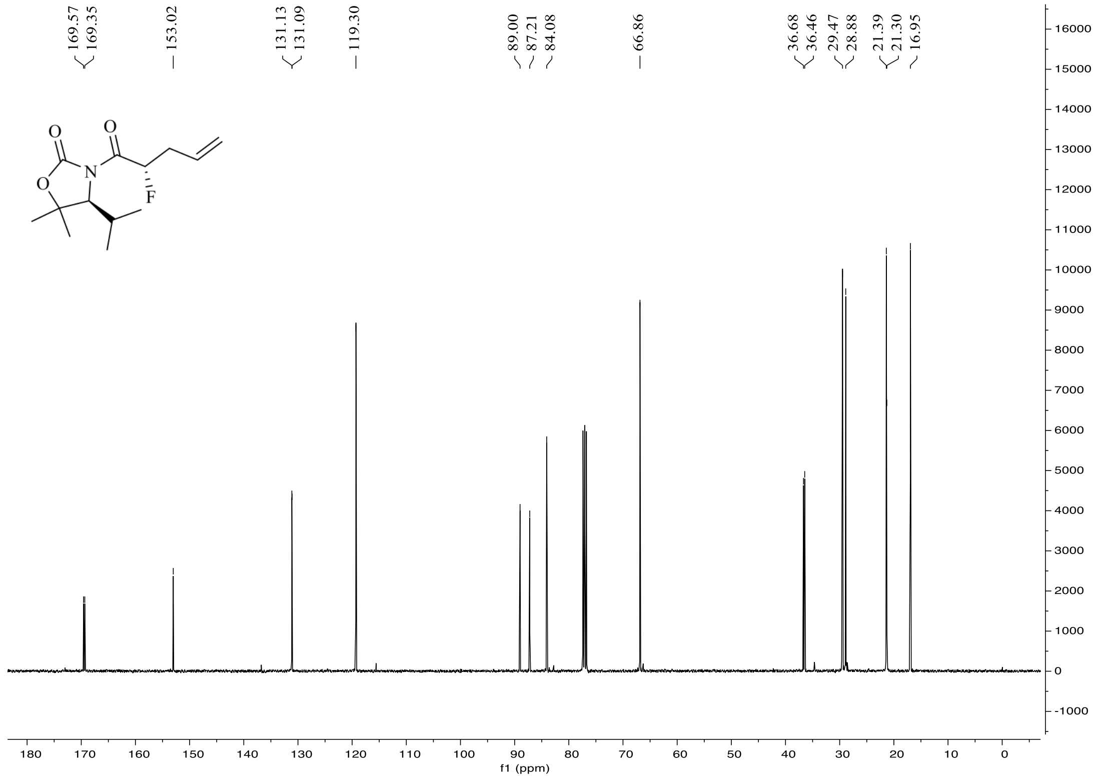

$^{19}F\{^{1}H\}$ NMR (367 MHz, $CDCl_3$) of **54**

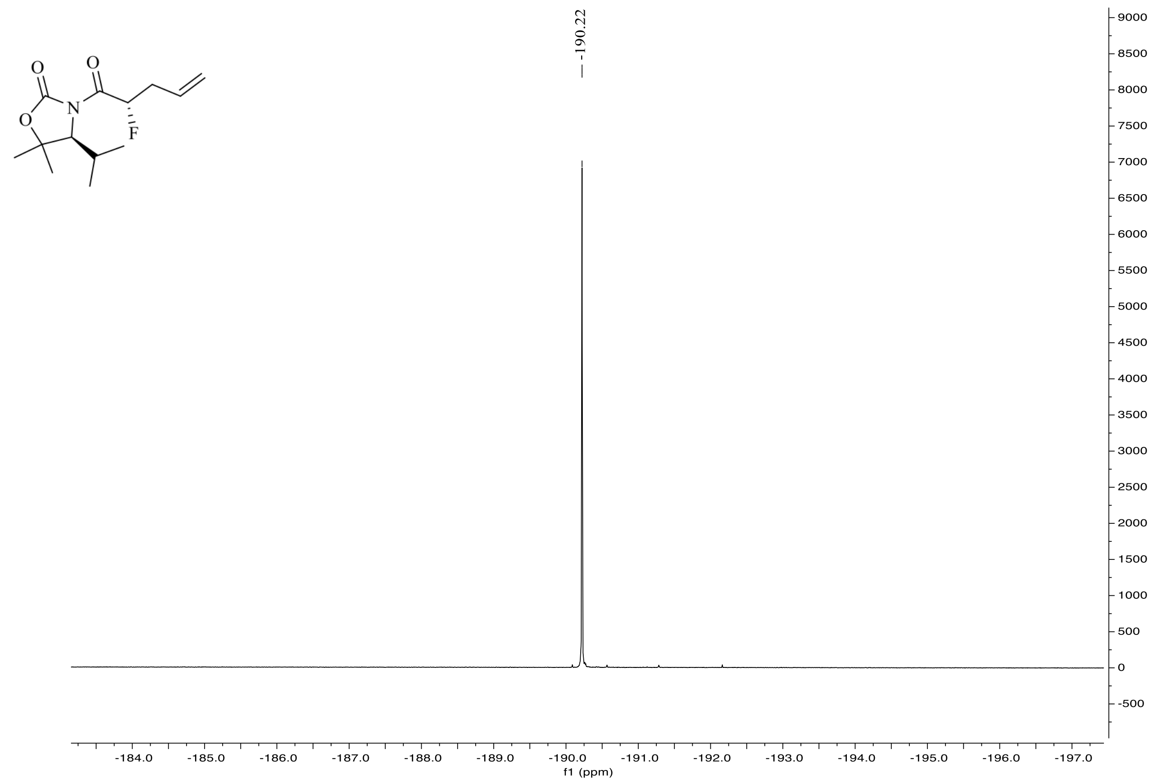

^{19}F NMR (367 MHz, $CDCl_3$) of **54**

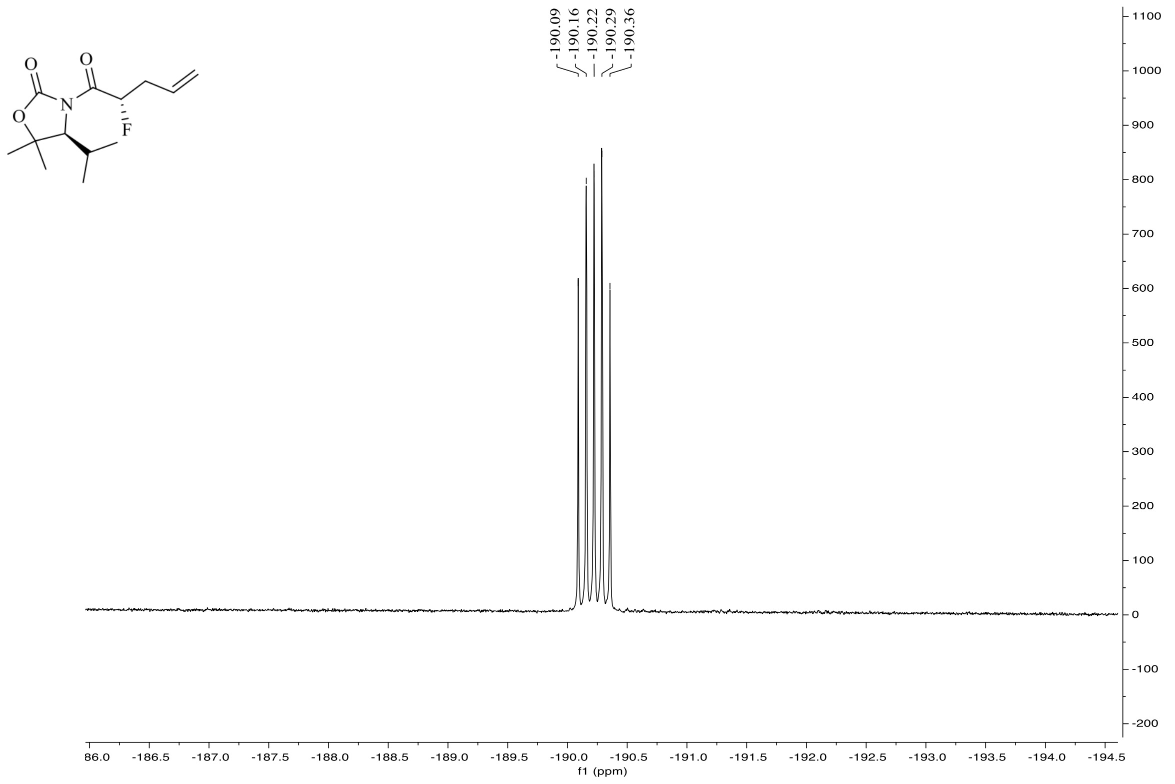

^{1}H NMR (400 MHz, $CDCl_3$) of **55**

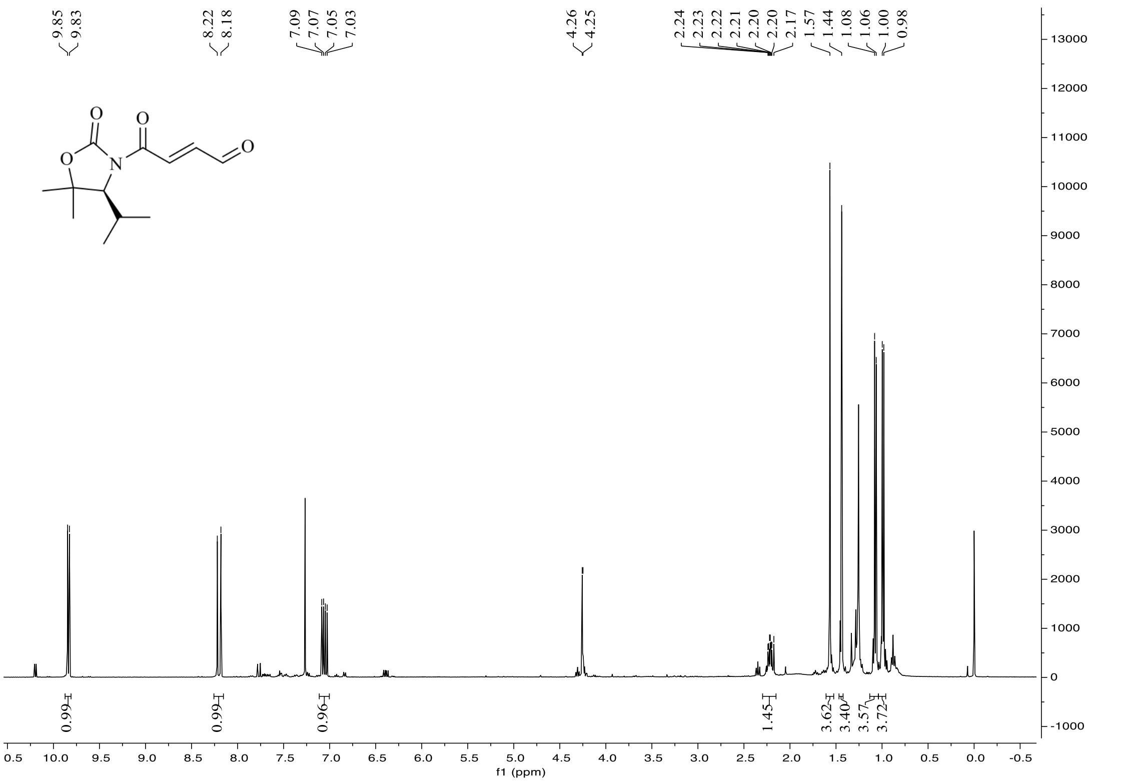

$^{13}C\{^{1}H\}$ NMR (100 MHz, $CDCl_3$) of **55**

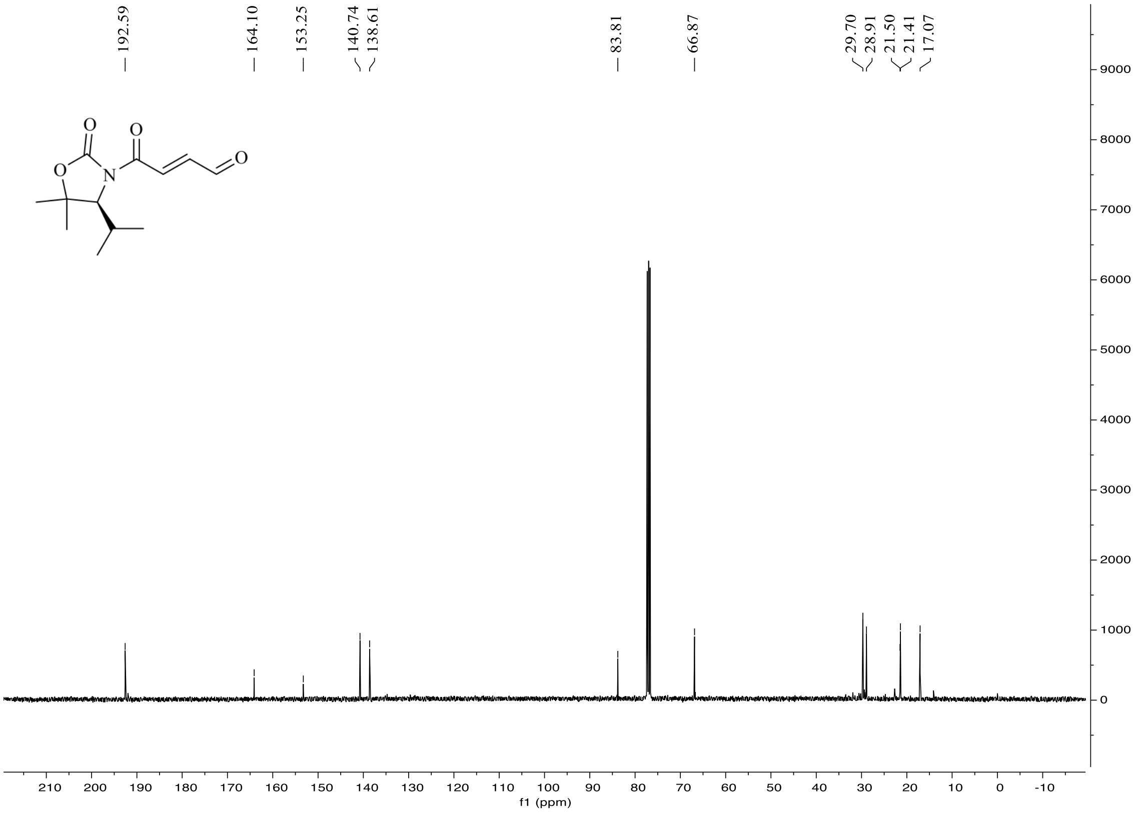

^{1}H NMR (400 MHz, $CDCl_3$) of **57**

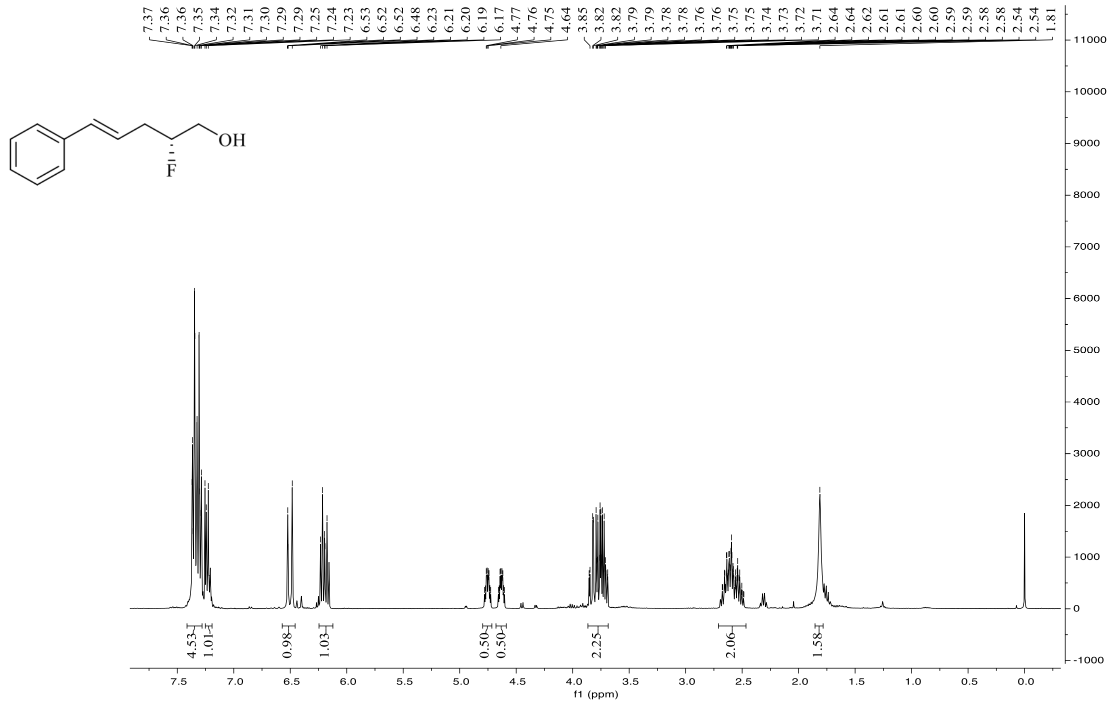

^{13}C{^{1}H} NMR (100 MHz, $CDCl_3$) of **57**

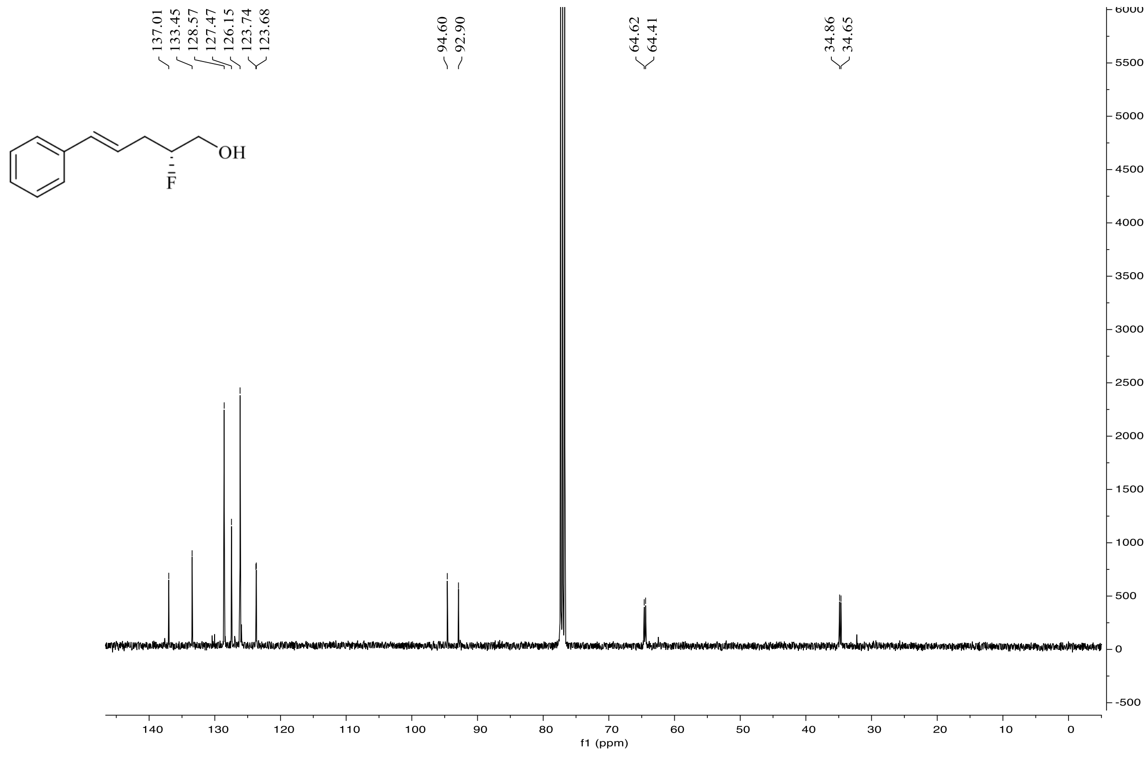

$^{19}F\{^1H\}$ NMR (367 MHz, $CDCl_3$) of **57**

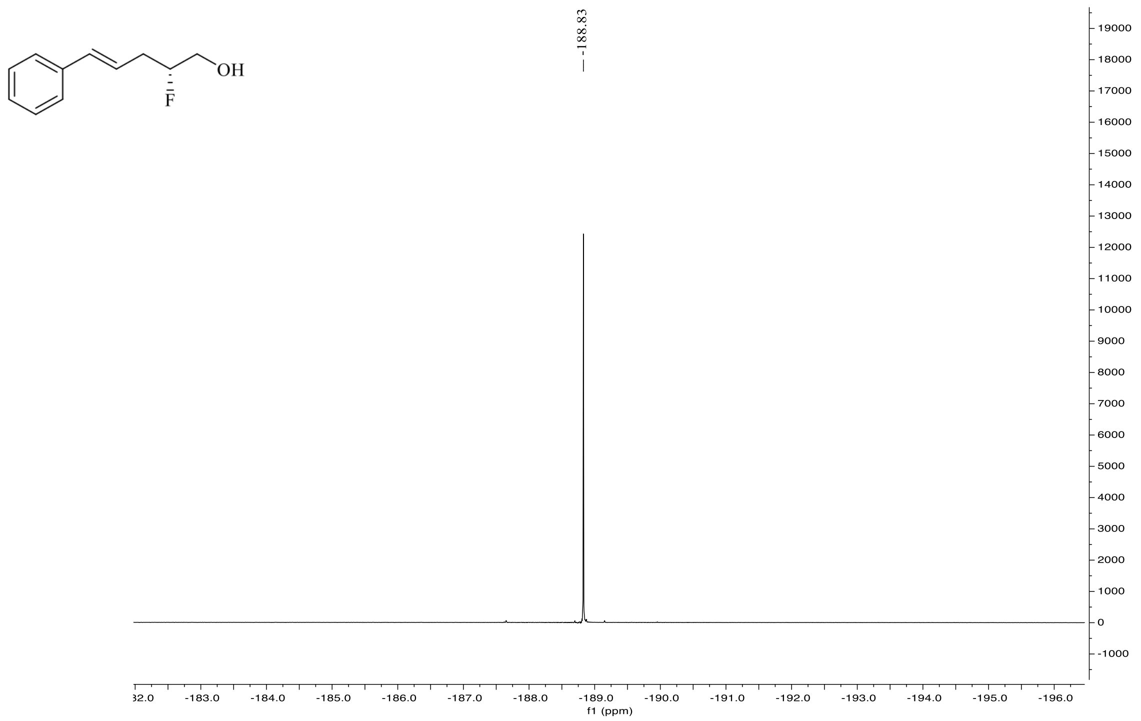

^{19}F NMR (367 MHz, $CDCl_3$) of **57**

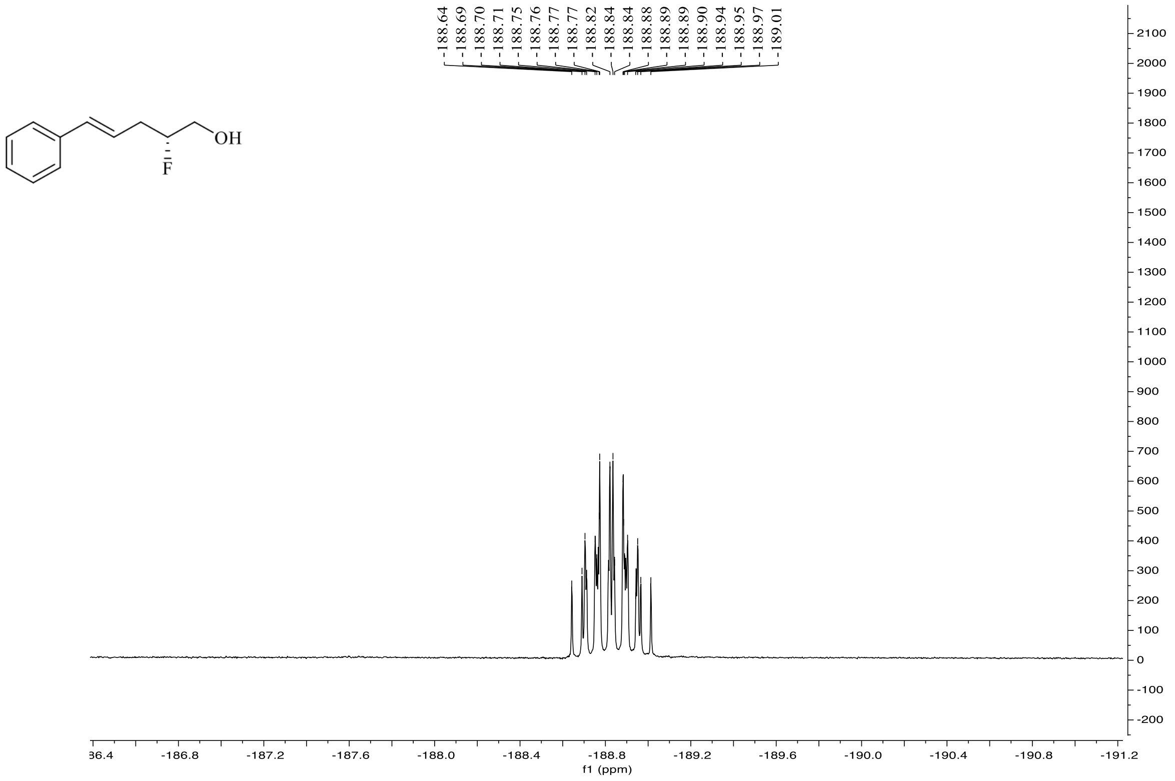

^{1}H NMR (400 MHz, $CDCl_3$) of **58**

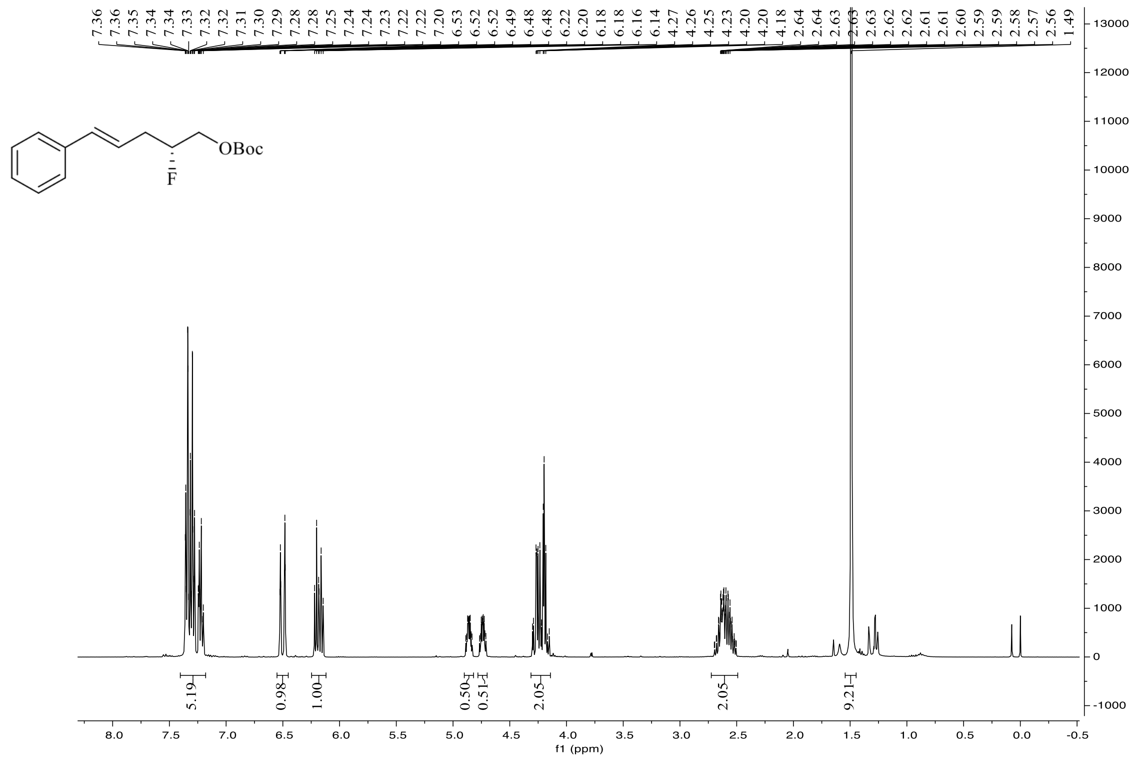

^{13}C{^{1}H} NMR (100 MHz, $CDCl_3$) of **58**

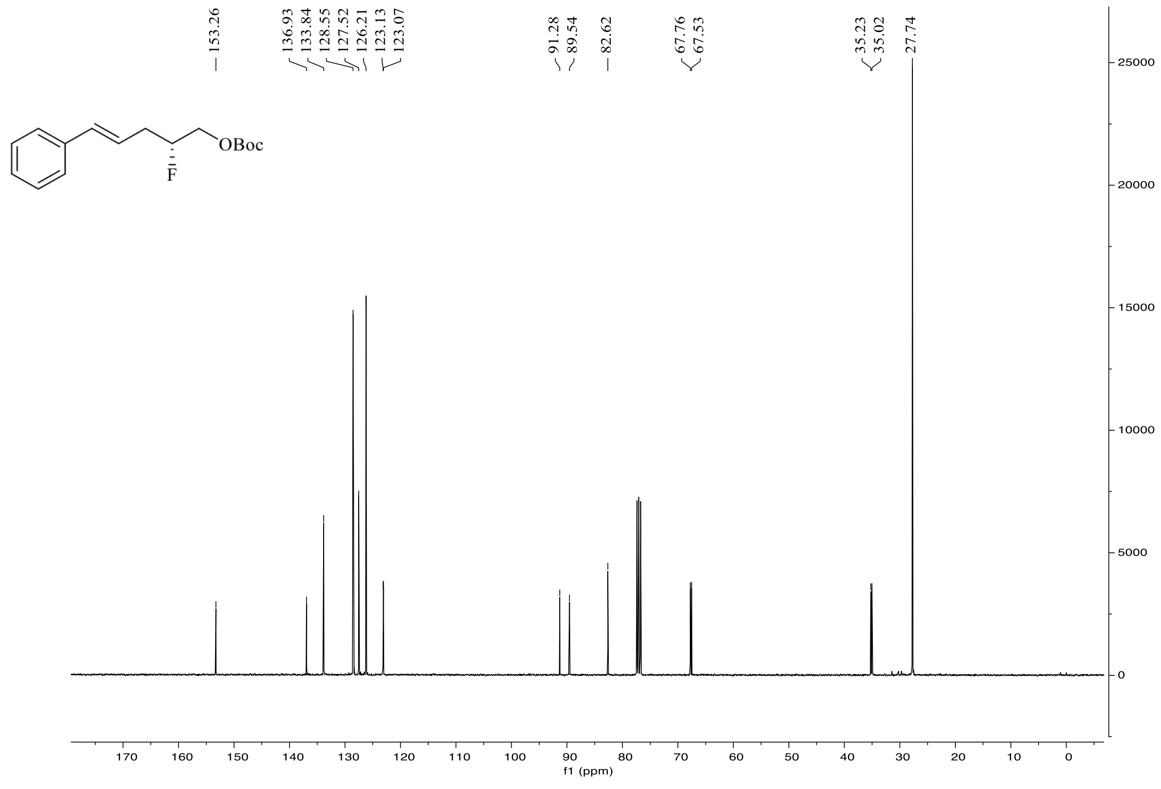

$^{19}F\{^1H\}$ NMR (367 MHz, $CDCl_3$) of **58**

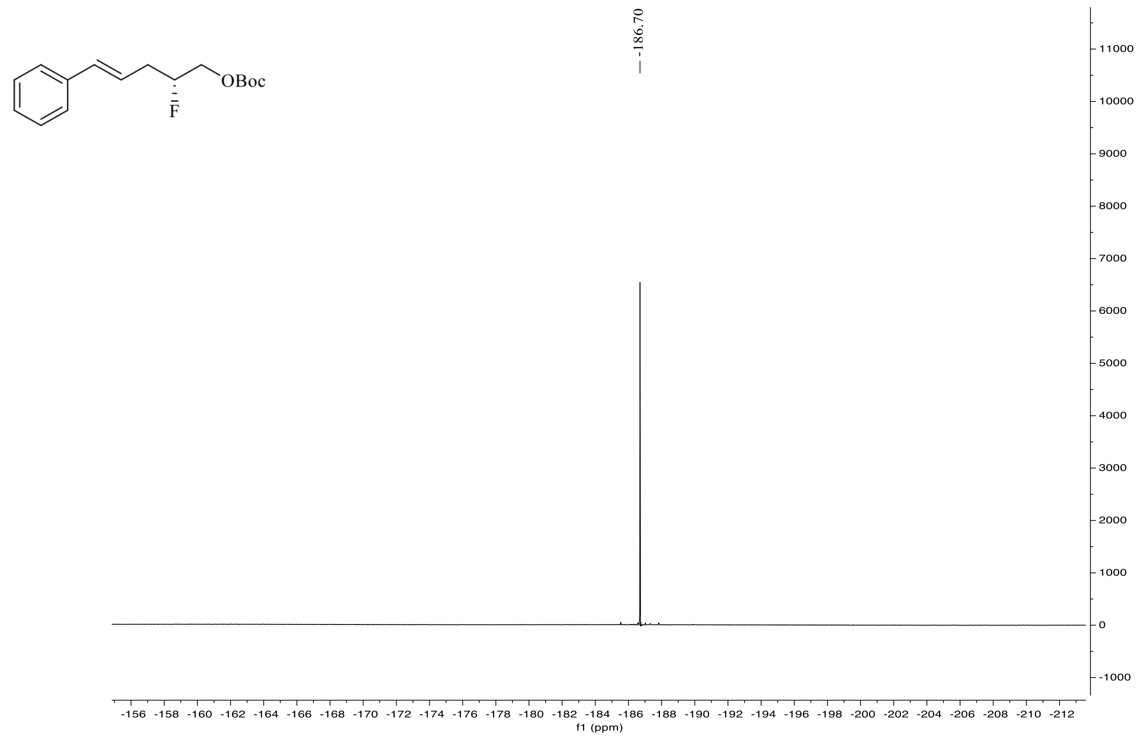

^{19}F NMR (367 MHz, $CDCl_3$) of **58**

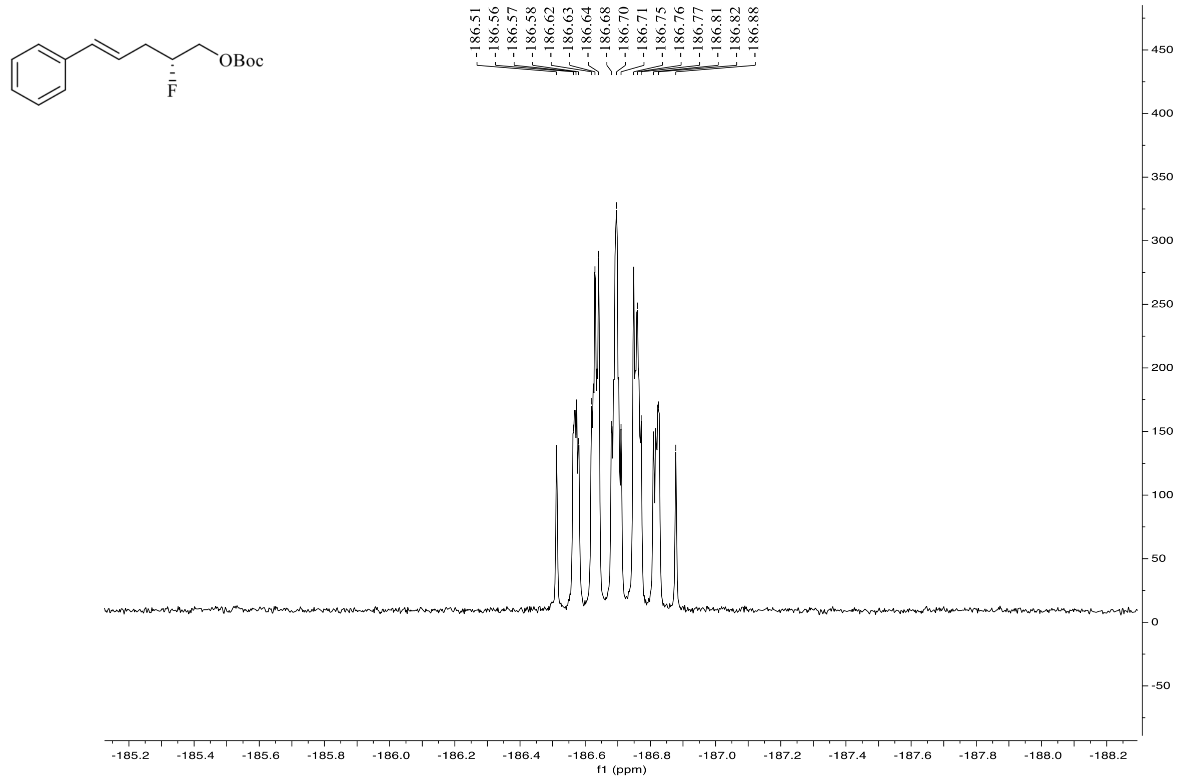

^{1}H NMR (300 MHz, $CDCl_3$) of **61**

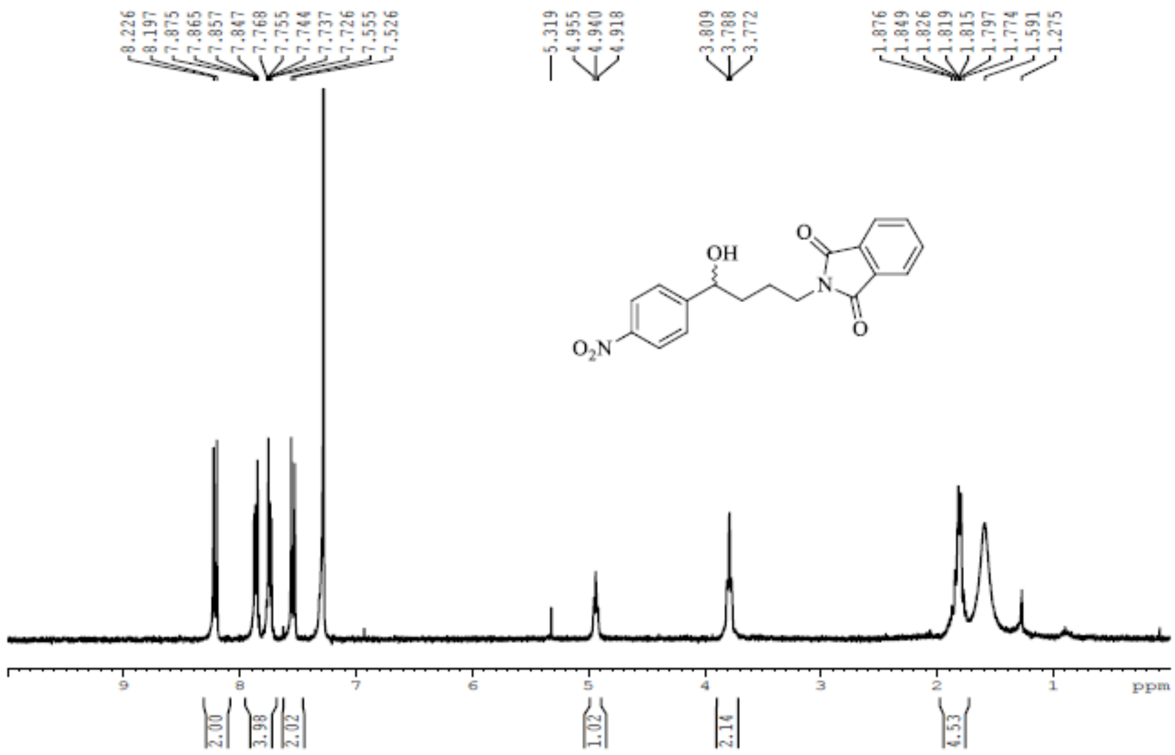

^{13}C{^{1}H} NMR (75 MHz, $CDCl_3$) of **61**

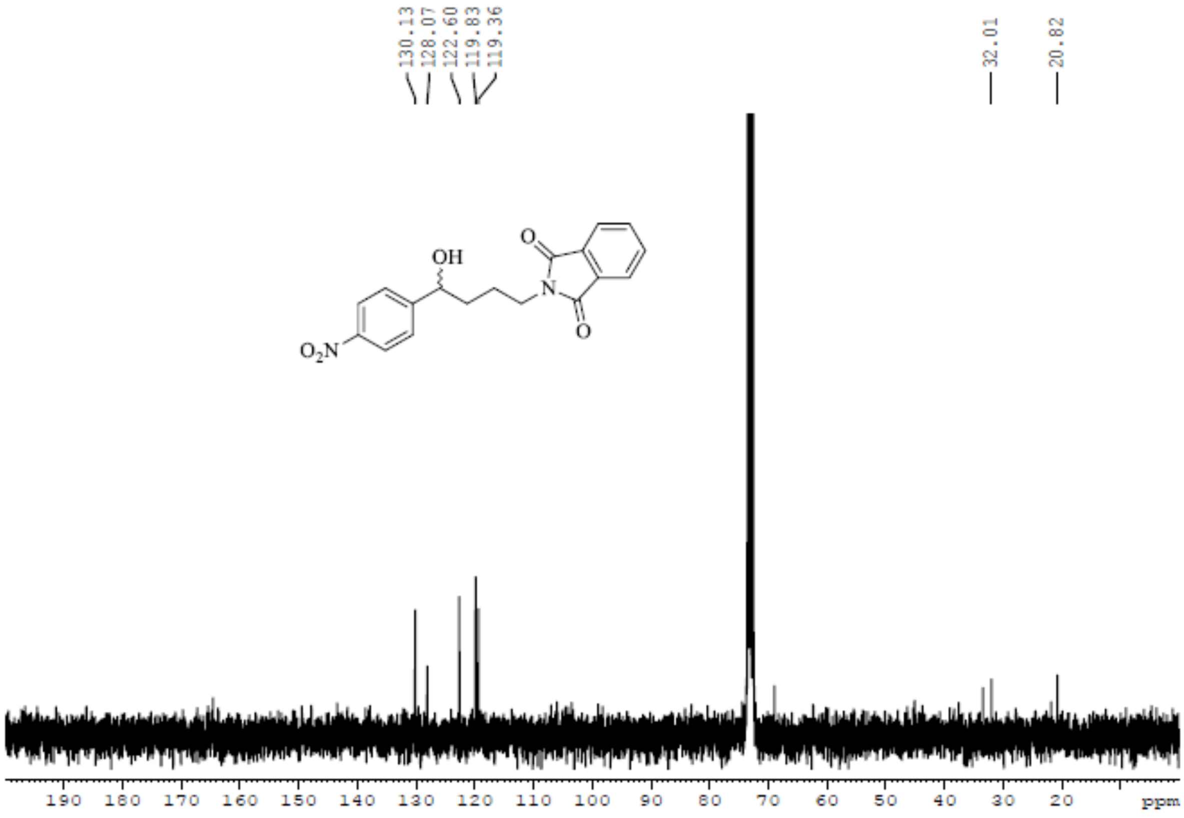

^{1}H NMR (300 MHz, $CDCl_3$) of **62**

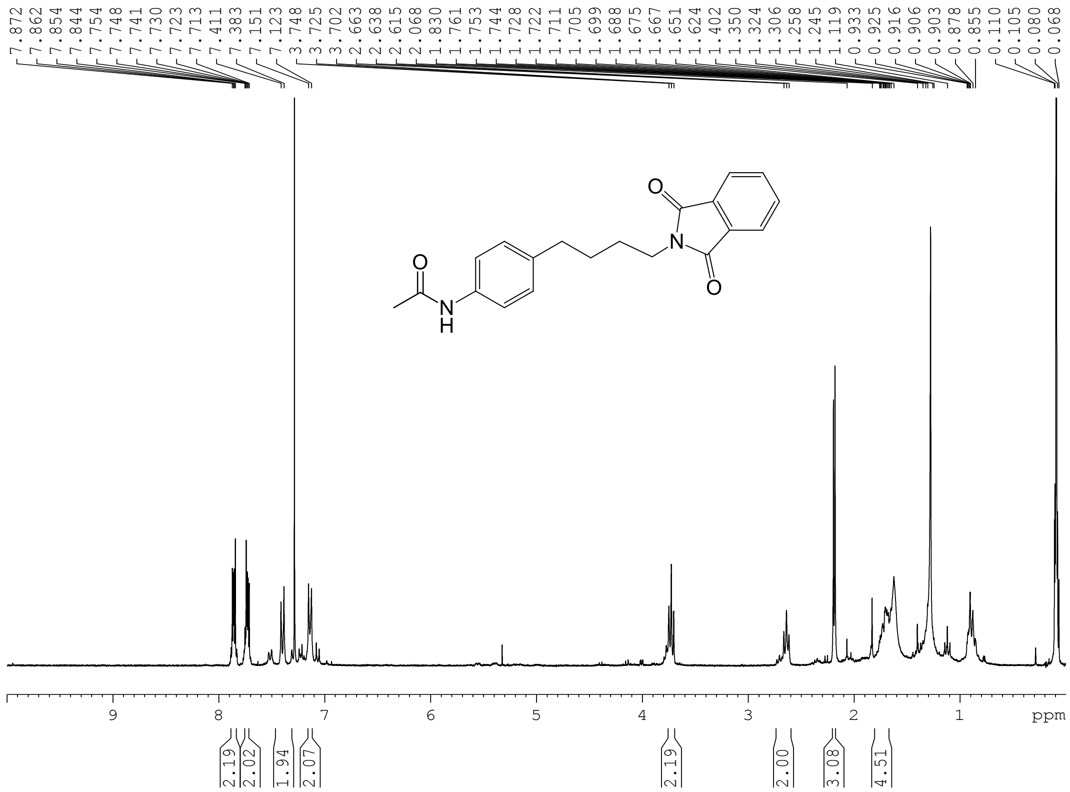

$^{13}C\{^{1}H\}$ NMR (75 MHz, $CDCl_3$) of **62**

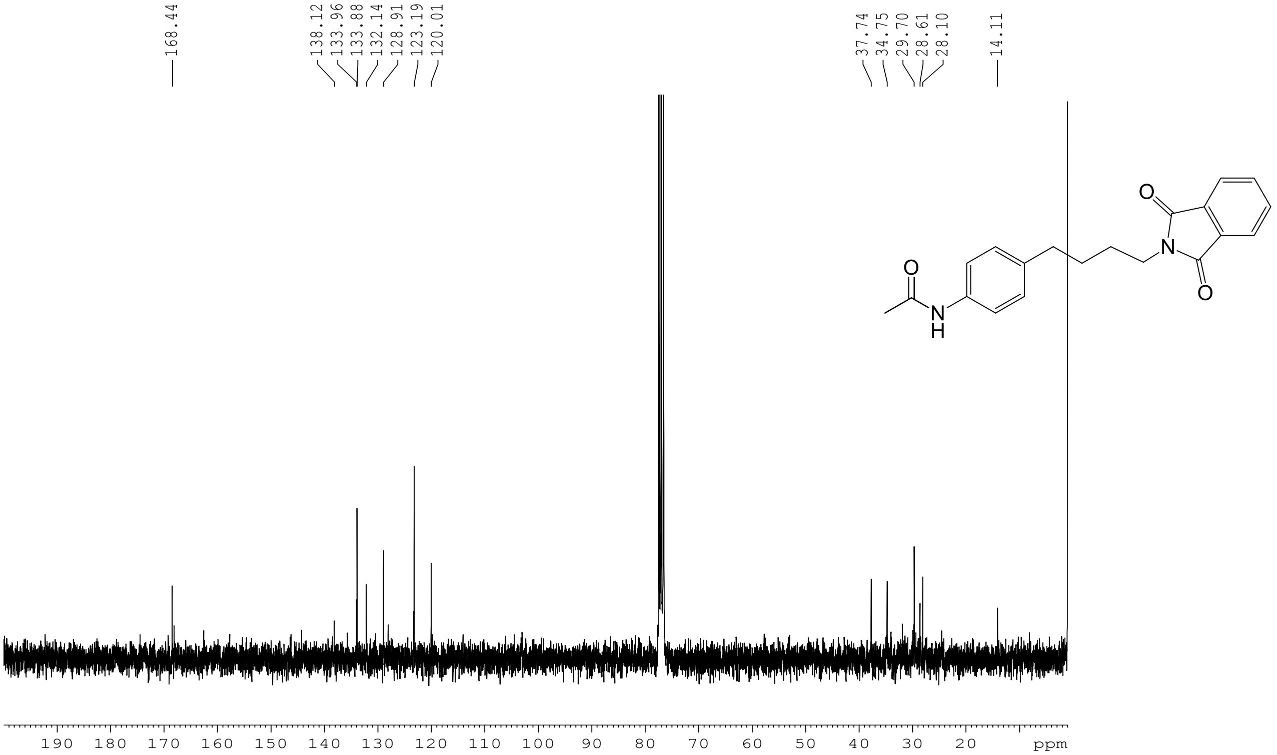

NMR simulations of 6a/b

Expansions from the simulated and experimental NMR spectra of **6a** are displayed below. From these simulations, a set of *J* values was obtained (see Table overleaf). It is acknowledged that the match between simulated and experimental NMR spectra is not perfect, which places some uncertainty on the true *J*-values.

^{1}H NMR:

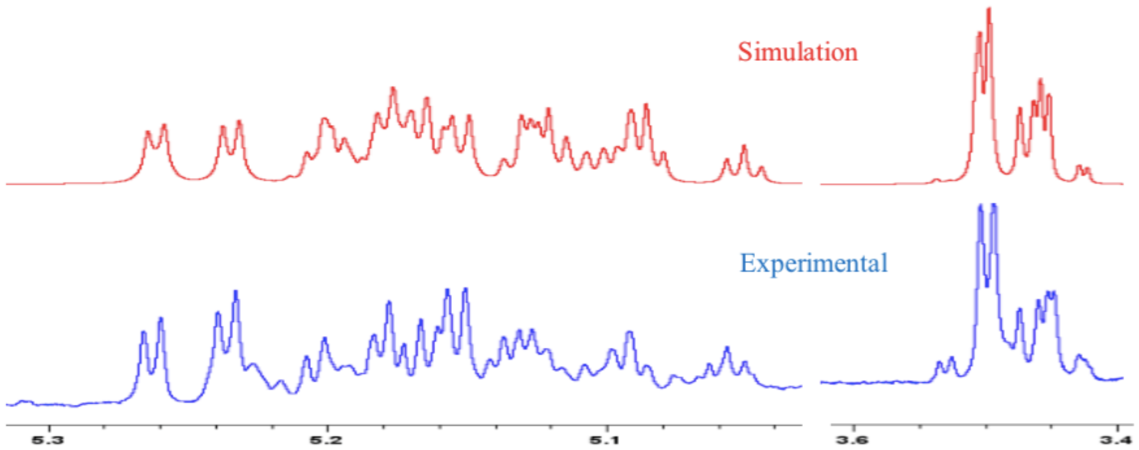

^{19}F NMR:

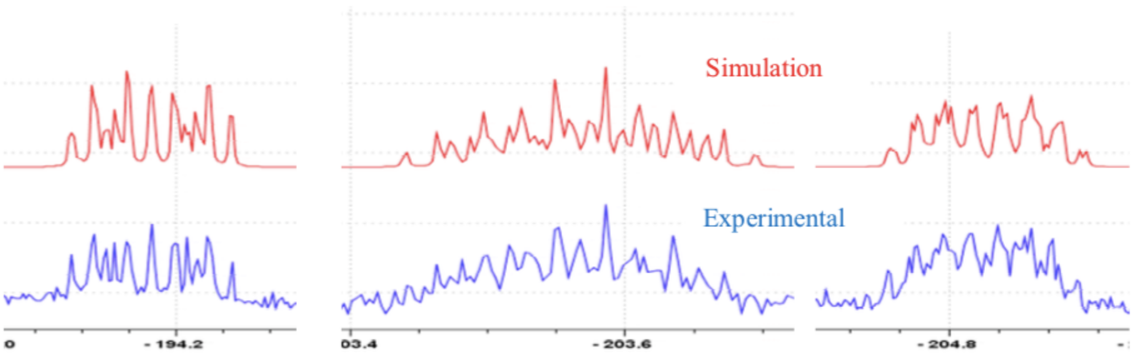

NMR coupling constants (Hz) of **6a**:

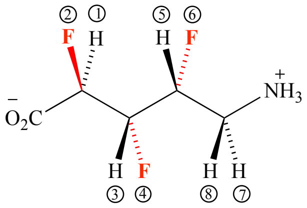

	②	③	④	⑤	⑥	⑦	⑧
①	49.35	3.66	16.08	0.00	0.00	0.00	0.00
②		21.09	11.00	3.00	10.00	0.00	0.00
③			47.00	3.80	21.00	0.00	0.00
④				15.00	11.00	1.00	1.00
⑤					45.00	7.00	3.50
⑥						18.00	30.00
⑦							17.80

Expansions from the simulated and experimental NMR spectra of **6b** are displayed below. From these simulations, a set of *J* values was obtained (see Table overleaf). It is acknowledged that the match between simulated and experimental NMR spectra is not perfect, which places some uncertainty on the true *J*-values.

^{1}H NMR:

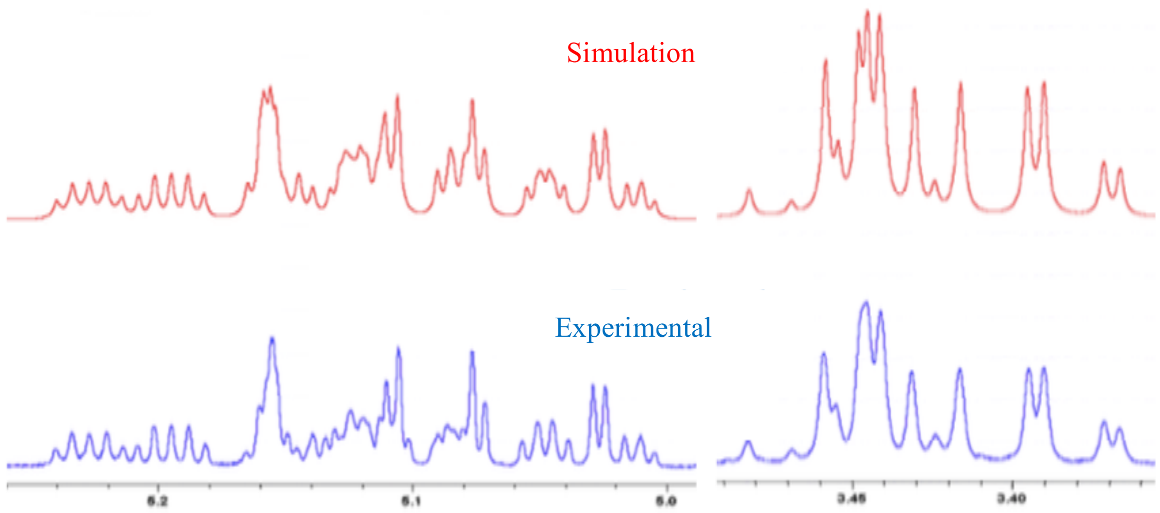

^{19}F NMR:

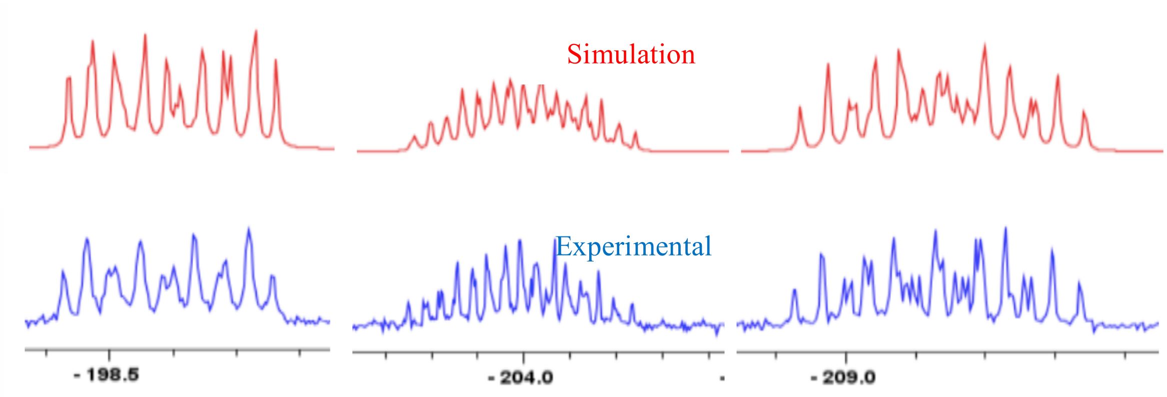

NMR coupling constants (Hz) of **6b**:

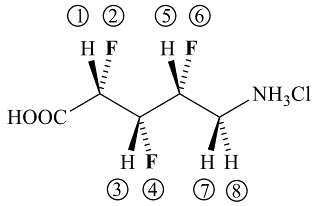

	②	③	④	⑤	⑥	⑦	⑧
①	49.00	2.95	28.80	0.00	0.00	0.00	0.00
②		23.50	12.00	0.00	12.00	0.00	0.00
③			44.50	3.70	21.00	0.00	0.00
④				19.40	12.00	0.00	0.00
⑤					45.23	2.80	9.50
⑥						33.00	15.20
⑦							14.26

References

[1] R. Cheerlavancha, A. Lawer, M. Cagnes, M. Bhadbhade, L. Hunter, *Org. Lett.* **2013**, *15*, 5562–5565.

[2] A. O. Kolodyazhnaya, O. O. Kolodyazhnaya, O. I. Kolodyazhnyi, *Russian Journal of General Chemistry* **2010**, *80*, 549–562.

[3] M. Marigo, D. Fielenbach, A. Braunton, A. Kjærsgaard, K. A. Jørgensen, *Angew. Chem. Int. Ed.* **2005**, *44*, 3703–3706.

[4] T. R. Hoye, C. S Jeffrey, Feng Shao, *Nature Protocol*s **2007**, *2*, 2451.

[5] G. Deniau, A. M. Z. Slawin, T. Lebl, F. Chorki, J. P. Issberner, T. van Mourik, J. M. Heygate, J. J. Lambert, L.-A. Etherington, K. T. Sillar, D. O'Hagan, *ChemBioChem* **2007**, *8*, 2265–2274.
